# The 13th Annual Asia Pacific Conference on Vision, 2017

**DOI:** 10.1177/2041669517728789

**Published:** 2017-10-16

The 13th annual Asia Pacific Conference on Vision was held in Tainan, Taiwan. The APCV2017 organizing committees have put together a full and exciting meeting this year with over 161 presentations, including three invited keynote speech, six invited symposia, four self-organized symposia, six oral sessions and three poster sessions. There were 189 researchers from 18 different countries who presented work on a broad range of vision science. The organizing committee thank them all for their contributions. The Abstracts are provided below. Keynote talks are presented first and then the others are listed by session.

## APCV Council

**Table:** 

David Crewther	Centre for Human Psychophysiology, Swinburne University
Xu Hong	Division of Psychology, School of Humanities and Social Science, Nanyang Technological University
Pi-Chun Huang	Department of Psychology, National Cheng Kung University
Kowa Koida	Electronics-Inspired Interdisciplinary Research Institute, Toyohashi University of Technology
Sang-Hun Lee	Brain and Cognitive Sciences, Seoul National University
Mowei Shen	Department of Psychology, Zhejiang University

## APCV2017 Local Organizing Committee

**Table:** 

Pi-Chun Huang	*Chair*, National Cheng Kung University, Taiwan
Chuan-Chin Chiao	National Tsing Hua University, Taiwan
Li Jingling	China Medical University, Taiwan
Chun-Chia Kung	National Cheng Kung University, Taiwan
Chia-Huei Tseng	Tohoku University, Japan
Chun-I Yeh	National Taiwan University, Taiwan

## Program Committee

**Table:** 

Chien-Chung Chen	National Taiwan University, Taiwan
Su-Ling Yeh	National Taiwan University, Taiwan

## Keynotes

## Adult Cortical Plasticity

**Robert Hess**

Department of Ophthalmology, McGill University, Montreal, Canada Email: robert.hess@mcgill.ca

## 

Hubel and Wiesel, Nobel Laureates in 1981, were the first to discover that columns exist in the visual cortex representing left and right eye inputs (ocular dominance columns), and they also found that there is a critical period for visual development that occurs within the first year of life. More recently, however, it has become clear that some plasticity remains into adulthood. Recent work shows that this plasticity extends to monocular contrast sensitivity as well as ocular dominance (OD) in adults, which could potentially lead to direct therapeutic benefit. Neuroplastic changes can occur as the result of perceptual training, non-invasive brain stimulation or short-term visual deprivation. Short-term visual deprivation in adults improves sensitivity of the deprived eye and reduces sensitivity of the non-deprived eye, allowing the two eyes' inputs to be rebalanced at the level of binocular integration. In this talk, I will review the evidence for adult cortical plasticity using a variety of approaches.

## Understanding Human Vision With Fast Periodic Stimulation

**Bruno Rossion**

University of Louvain, Leuven, Belgium Email: bruno.rossion@uclouvain.be

## 

When the human brain is stimulated at a rapid periodic frequency rate, it synchronizes its activity to this frequency, leading to periodic responses recorded in the EEG (Adrian & Matthews, 1934). In vision, periodic stimulation has been used essentially to investigate low-level processes and attentional effects in the primary visual cortex, under the term “Steady-State Visual Evoked Potentials” (ssVEPs; Norcia et al., 2015 for review; Regan, 1966). This approach has now been extended and refined to understand higher level visual functions, in particular the categorization of complex visual forms such as human faces, objects, and words. This talk will summarize studies carried out over the last few years illustrating the unique strengths of this fast periodic visual stimulation approach: (a) the objective (i.e., exactly at the experimentally defined frequency rate) definition of neural activity related to visual recognition; (b) the very high signal-to-noise ratio allowing to rapidly measure visual recognition processes in difficult to test populations (e.g., infants and children, patients); (c) the independence from explicit behavioral responses; and (d) the first identification of objective markers of visual integration (binding). Contrary to widespread assumption, this approach also provides precise information in the time-domain and has started to fully characterize the spatio-temporal course of visual recognition in a rapidly changing natural scene.

Adrian, E. D., & Matthews, B. H. C. (1934). The Berger rhythm: Potential changes from occipital lobes in man. *Brain*, *57*, 355–385.10.1093/brain/awp32420058345Norcia, A. M., Appelbaum, L. G., Ales, J. M., Cottereau, B. R., Rossion, B. (2015). The steady-state visual evoked potential in vision research: A review. *Journal of Vision*, *15*, 1–46.10.1167/15.6.4PMC458156626024451Regan, D (1966). Some characteristics of average steady-state and transient responses evoked by modulated light. *Electroencephalogr Clin Neurophysiol*, *20*, 238–248.10.1016/0013-4694(66)90088-54160391

## Object Recognition in Inferotemporal Cortex: From Visual Features to Semantics

**Keiji Tanaka**

RIKEN Brain Science Institute, Hirosawa, Japan Email: keiji@riken.jp

## 

The inferotemporal cortex (IT) of the monkey brain is the final stage of the ventral visual pathway, which is thought to be responsible for visual object recognition. Because our visual recognition is categorical in nature, object categories may be represented in IT and earlier stages. However, by carefully determining the stimulus selectivity of individual cells in the monkey IT, we previously found that single IT cells’ selectivity was determined by moderately complex features, defined by physical parameters. A remaining possibility is that object categories are represented by the response pattern of a population of IT cells. By recording responses of many IT cells to a fixed set of 1,084 object images, we examined this possibility. Responses of only one or two cells were tested at a time, but by repeating the recording for several months in two monkeys, we obtained responses of 674 cells to the stimulus set. By seeing the response table from the stimulus side, we can analyze the response pattern evoked by each of the stimuli over the 674 cells. We found that two stimuli belonging to the same category tended to evoke similar response patterns whereas those belonging to distant categories evoked different response patterns. When the 1,084 objects were plotted according to the dissimilarity of response patterns, objects of the same category clustered. Even the hierarchical structure of object categories appeared there. Thus, although the stimulus selectivity of individual IT cells is determined in the domain of moderately complex features, which is still physical, by having multiple cells with selectivity for various features, responses of a population of IT cells represent object categories, which is semantic.

We have also examined the nature of local clustering of cells in the monkey IT. We previously found that cells responding to similar features clustered in a columnar local region in monkey IT. Is the local clustering of cells in monkey IT determined only in the domain of features? Since animals care about object categories rather than features, the local clustering of cells may be organized toward the representation of object categories. More concretely, we ask whether there are multiple groups of cells responding to different features yet associated with the same object categories in a local IT region. To record many (∼50) cells in a local region, we have developed a technique for chronic recordings with an electrode that remains in the brain for a few weeks and is advanced day by day. Responses of recorded cells were examined with a fixed set of 850 object images (50 images each for 17 object categories). Most pairs of cells recorded from a local IT region showed similar categorical selectivity. When we examined their responses to the members of their commonly effective object category, whereas many of the pairs also had similar selectivity, others showed no similar or even complimentary selectivity. These results suggest that multiple groups of cells responding to different features yet associated with the same object categories cluster in a local IT region.

## Symposium Sessions Neural Basis of Visual Perception

## Plasticity for Stimulus Selectivity in the Visual Cortex of Adult Mice Induced by Patterned Optogenetic Stimulation

**Andrea Benucci**

Laboratory for Neural Circuits and Behavior, RIKEN Brain Science Institute, Saitama, Japan Email: andrea.benucci@riken.jp

## 

Functional plasticity in cortical networks plays an important role in a variety of learning and adaptive behaviors. However, little is known about its functional properties in vivo and in adult animals. In this study, we first established an in vivo preparation for simultaneous imaging and spatially patterned optogenetic stimulation based on a digital-micromirror-device (DMD). In awake, adult mice expressing GCaMP8 and ChrimsonR in the primary visual cortex (V1), we paired DMD stimulation of a single neuron (the driver) to a delayed stimulation (10 ms) of several tens of surrounding neurons aiming to induce driver-surround spike-timing dependent plasticity (STDP). We found that the preferred orientation of neurons in the surround changed depending on the preferred orientation of the driver, with a significant increase in the fraction of neurons tuned to the orientation orthogonal to that of the driver. Despite these plastic changes, the network as a whole could still reliably encode stimulus orientations thanks to a subgroup of non-plastic cells. Such neurons were vigorously responding to the visual stimulation, while weakly responsive cells exhibited larger degrees of plasticity. In an effort to explain the orthogonal effect, we used a standard *ring* model combined with an STDP rule. Under the assumption that the driver’s optogenetic stimulation elicited network’s activations more broadly tuned in the orientation domain than visual stimulation, the model could qualitatively explain the orthogonal effect. In conclusion, this study reveals a previously unreported functional specificity of in vivo cortical plasticity, where the activity of just one cell can induce network-level, orientation-specific changes. Furthermore, the discovery of an inverse relationship between plasticity and visual responsiveness suggests a general mechanistic principle for the implementation of an “exploration-exploitation” learning rule in adult cortical networks.

## 3D Topology of Orientation Columns in Visual Cortex Revealed by Functional Optical Coherence Tomography

**Manabu Tanifuji**

RIKEN Brain Science Institute, Saitama, Japan Email: tanifuji@riken.jp

## 

Optical intrinsic signal imaging (OISI) enabled us to visualize spatial arrangement of the orientation columns across the cortical surface and led us to discovery of the orientation singularity (pinwheel). Because of integrated information along the cortical depth axis in OISI, on the other hand, the detailed structure along depth axis has not been more than speculated. Here, we visualized the functional structure along depth axis of orientation columns in cat visual cortex with µicrometer scale spatial resolution and millimeter scale measurement range by means of functional optical coherence tomography (fOCT). fOCT resolves changes in light scattering at different depth of the tissue that reflect strength of neural activation. As expected, in iso-orientation domains, the preferred orientation did not change along depth axis and orientation preference of the vertically elongated regions shifted gradually along the axis parallel to cortical surface. To our surprise, however, the orientation singularity was not always elongated vertically. It was often twisted, went parallel to the cortical surface or joined with other singularity. Thus, the orientation singularity is not a point in space that is replicated in multiple layers as we expected previously. It is rather like string running in various trajectories across layers.

## Neuronal Processing of Cross-Finger Motion Integration in the Primary Somatosensory Cortex

**Yu-Cheng Pei**

Physical and Rehabilitation, Chang Gung Memorial Hospital, Taipei, Taiwan Email: yspeii@adm.cgmh.org.tw

## 

Dynamic object manipulation involves tactile motion perceived through multiple fingerpads. Neuronal tuning properties to direction and orientation in the primary somatosensory (S1) cortex have been characterized but the integration of tactile motion across fingers is not yet known. To this end, we used a multi-digit tactile motion stimulator with scanning balls engraved with square-wave gratings to present tactile motion to two nearby fingerpads and recorded the neuronal responses in areas 3b, 1 and 2 of anesthetized monkeys using multi-channel microelectrode arrays. Specifically, either one (one-finger condition) or both (two-finger condition) of the two fingers were presented with the motion stimuli, yielding a variety of combination of stimulus directions presented to the two nearby fingers. We found that a majority of motion-sensitive neurons have two-finger receptive fields. Comparing the activities obtained in one- and two-finger conditions in motion-sensitive neurons, 40% of neurons showed direction/orientation selectivity in the two-finger condition but no selectivity in the one-finger condition. Furthermore, the motion integration observed in the two-finger condition was mainly mediated by a nonlinear suppression of neuronal activities. These results indicate that motion integration across fingers is commonly observed in S1 neurons and can be accounted for by nonlinear suppressions of convergent inputs emanating from two fingers, a novel nonclassical receptive field property that is first reported in primate S1.

## Visual Attention Mechanisms in the Pulvinar-Cortex Circuits

**Huihui Zhou**

Shenzhen Institutes of Advanced Technology, Chinese Academy of Sciences, Beijing, China Email: hh.zhou@siat.ac.cn

## 

Visual attention is important in our daily life. For example, we are often facing overwhelming visual inputs at a given moment that easily exceed the processing power of our brain. Attention allows us to focus on a small portion of the inputs, which are important for our behaviors, while ignoring the other less important stimuli. After a long history of studies in animals, it becomes evident that visual attention mechanisms involve a large number of brain areas and complex interactions between them. The pulvinar, the largest nucleus of the primate thalamus, is reciprocally connected with almost all visual areas in cortex. Although numerous studies suggest its important role in visual attention, the mechanisms of attentive stimulus processing in the pulvinar-cortex loop are still unclear. We investigated the interaction between the pulvinar and area V4 in a spatial attention task in non-human primates. Attention enhances gamma oscillatory coupling between the two areas, and the V4 influence on pulvinar by a granger causality analysis. Furthermore, pulvinar deactivation leads to a reduction of attentional effects and sensory-evoked responses in V4. Thus, the cortical interaction with the pulvinar seems necessary for normal attention and sensory processing in visual cortex.

## Spatial Receptive Field of Color-Responsive Neurons in Macaque V1

**Chun-I Yeh**

Department of Psychology, National Taiwan University, Taipei, Taiwan Email: ciyeh@ntu.edu.tw

## 

Spatial receptive fields have been studied to understand the properties of color- and luminance-responsive neurons in the primary visual cortex V1. In macaque V1, many color-responsive neurons are highly selective for orientation and spatial frequency with drifting gratings (Johnson et al., 2001; Friedman et al., 2003). One might predict that the receptive field structures of color-responsive neurons would consist of multiple elongated on and off sub-regions (like simple cells). However, previous studies had shown mixed results: Some found simple cell-like receptive fields by using dense noise (Horwitz et al., 2007; Johnson et al., 2008), whereas others found receptive fields that were blub-like and less elongated when using sparse noise (Conway and Livingstone, 2006). Here, we measured spatial receptive fields of V1 color-responsive neurons with two different stimulus ensembles: Hartley gratings and binary sparse noise both consisted of equiluminance colors (red and green that represent different cone weights). Receptive fields were calculated by reverse correlation and fitted with the 2D Gabor function. We studied a total of 206 units in macaque V1 units and found that Hartley maps had significantly higher aspect ratios and greater numbers of subregions than sparse noise maps. There was a negative correlation between the circular variance measured with drifting gratings and the aspect ratio of the map (significant correlation was found in Hartley but not in sparse noise). Moreover, we found that band-pass color cells had significantly higher aspect ratios that low-pass color cells under all stimulus ensembles. In summary, the receptive field of color-responsive neurons may change accordingly with different stimulus ensembles. For neurons that are well tuned for orientation and spatial frequency, the tuning properties can be well predicted by their properties of receptive fields mapped with Hartley gratings.

## Neuronal Connection of Intrinsically Photosensitive Retinal Ganglion Cells: How Light Influence Physiological Functions

**Shih-Kuo Chen**

Life Science, National Taiwan University, Taipei, Taiwan Email: alenskchen@ntu.edu.tw

## 

Retinal ganglion cells (RGCs) in the retina receive input from classical photoreceptor rod and cone through bipolar cells and many lateral processing from amacrine cell. For image forming function, classic photoreceptor rods and cones are primary photon detectors located at the outer retina. However, recent studies showed that a small population of melanopsin-expressing intrinsically photosensitive retinal ganglion cells (ipRGCs) located at the inner retina is essential for many non-image forming visual functions. There are many subtypes of ipRGCs which provide environmental luminance signal for circadian photoentrainmnet, pupillary light reflex. Furthermore, ipRGCs could also influence the physiological functions of upstream order of retinal neuron such as dopaminergic amacrine cell and even the retinal development through intra-retinal axon collaterals. Therefore, ipRGC could convey luminance signal from the inner retina to outer retina to control light adaptation and simultaneously to the hypothalamus for other non-image forming function such as circadian clock modulation. Specifically, at the dark adapted stage, the amplitude of b-wave in electricretinalgram (ERG), which represents activity of cone on-bipolar cell, is relatively small. During the light adaptation process, the amplitude of b-wave increases gradually for the first 8–15 min. Elimination of ipRGCs completely blocks this elevation of b-wave during the light adaptation stage. Using D4 dopamine receptor agonist, we can rescue the impaired light adaptation in ipRGC elimination mice. Together, our results suggest that ipRGCs not only involve in non-image forming functions they also provide luminance signal retrogradely to the outer retina to modulate vision such as light adaptation.

## Binocular Depth Perception

## Reconciling Pictorial Depth With Stereopsis

**Dhanraj Vishwanath**

University of St Andrews, Scotland, UK Email: dv10@st-andrews.ac.uk

## 

Pictorial images are two-dimensional surfaces with a coherent pattern of lightness and color contrast that typically yield an illusory impression of 3D shape and space. This impression, however, is not as compelling as the impression of three-dimensionality in stereoscopic images. Similarly, the impression of depth and three-dimensionality in real scenes viewing with one eye—or even with both eyes when the objects are at a far distance—is not as compelling as that perceived in real scenes viewed from a near distance with both eyes. These less compelling instances of so-called “pictorial depth” are often believed to simply be high-level cognitive inferences of 3D shapes and distances based on visual cues, rather than constituting the perception of depth and three-dimensionality per se. Only those conditions that yield the compelling impression of depth called stereopsis—for example, due to the presence of binocular disparities or motion parallax—are conventionally thought to constitute depth perception proper. I present evidence and analysis that challenge this simple dichotomy. I argue that pictorial depth is automatic and mandatory, and that stereopsis and pictorial depth are both ways of perceiving depth and three-dimensionality but with different phenomenology and adaptive significance.

## Integration of Monocular and Binocular Cues to Depth Perception

**Christopher W. Tyler^1,2^**

^1^Smith-Kettlewell Eye Research Institute, San Francisco, USA

^2^Division of Optometry, City University of London, UK Email: cwt@ski.org

## 

To specify the depth structure of the visual scene, we need to decode the depth information from a variety of depth cues, many of which are inherently sparse across space. All depth cues are sparse wherever the scene has uniform shading. Additionally, disparity and motion are sparse wherever a correspondence cannot be established. When sparse, the net perceived depth is subject to discontinuities between the depths specified by each cue unless they are all consistently scaled in both the absolute and relative depth parameters. Thus, the cue combination map must have a rescaling mechanism to minimize the mismatches in depth scale among the sparse cues into the resultant depth map. If this cue rescaling process relies on Bayesian estimation of the reliability of each cue, the reliability estimation must have a time course somewhere in the range from neural response times to evolutionary time. The time course of depth cue rescaling was specified by matching the exponential stereoscopic depth image with the percept of a corresponding monocular depth image. The perceived depth dynamics had time constants up to several seconds that varied among cues, suggesting that the Bayesian reweighting based in reliability time course is of the order of perceptual adaptation times.

## Acknowledgments

This study was supported by AFOSR FA9550-09-1-0678.

## Binocular Correlation and Matching Computations Determine Depth Perception In a Weighted Parallel Manner

**Ichiro Fujita**

Center for Information and Neural Networks, Osaka University; Osaka, Japan Email: fujita@fbs.osaka-u.ac.jp

## 

The first step to compute binocular disparity is achieved in the primary visual cortex by a process similar to calculation of local cross-correlation between left and right retinal images. The correlation-based neural signals convey information about false disparities as well as the true disparity. Processing at later stages then eliminates these false responses in the initial disparity detectors in order to encode only disparities of the features correctly matched between the two eyes. For a simple stimulus configuration, a feed-forward nonlinear process can transform the correlation signal into the match signal. Psychophysics in human observers suggests that depth judgement is determined by a weighted sum of the correlation and match signals rather than dependent entirely on the latter. The relative weight varies with spatial and temporal parameters of the stimuli, allowing adaptive recruitment of the two computations under different visual circumstances. A full transformation from correlation-based to match-based representation occurs at the neuronal population level in cortical area V4. Neurons in area V5/MT represent disparity in a manner intermediate between the correlation and match signals. We propose that the correlation and match signals in these areas contribute to depth perception in a weighted, parallel manner.

## Pooling in V1 Parameter Space Enhances Accuracy of Binocular Matching

**Izumi Ohzawa**

Center for Information and Neural Networks, Osaka University, Osaka, Japan Email: ohzawa@fbs.osaka-u.ac.jp

## 

The key problem of stereopsis is traditionally defined as accurately finding the positional offsets of corresponding object features between left and right images. Here, we demonstrate that the problem must be considered in a four-dimensional parameter space, with respect not only to shifts in position (X, Y) but also spatial frequency (SF) and orientation (OR). The proposed model pools outputs of binocular energy units linearly over the multi-dimensional V1 parameter space (X, Y, SF, OR). Contrary to a common expectation that pooling reduces neural selectivity, our theoretical analyses and physiological experiments show that many binocular neurons achieve sharpened binocular tuning properties by pooling the output of multiple neurons with relatively broad tuning. Pooling in the space domain sharpens disparity-selective responses in the SF domain so that the responses to combinations of unmatched left–right SFs are attenuated. Conversely, pooling in the SF domain sharpens disparity selectivity in the space domain, reducing the possibility of false matches. Analogous effects are observed for the OR domain in that the spatial pooling sharpens the binocular tuning in the OR domain. Such neurons can achieve sharpened tuning for relative orientation disparity. Therefore, pooling in V1 space enhances left–right matching accuracy of disparity-selective neurons.

## New Inquiries into Vision, Attention, and Awareness

## Multisensory Influences on Visual Attention

**Marcia Grabowecky**

Department of Psychology, Northwestern University, Evanston, IL, USA Email: grabowecky@northwestern.edu

## 

Perceptual research has matured, and a great deal is now known about individual perceptual modalities, and about how attention functions within them. However, we experience the world as multisensory and integrated, and attention is most often directed to multisensory events. I will discuss some of our work over the last decade using visual search, attentional cuing, and binocular rivalry paradigms. Data from these paradigms suggest pervasive influences from auditory and haptic processing on visual perception and attention. Whereas some of these interactions depend on well-learned correspondences, such as the visual appearance of a cat and its accompanying “meow” vocalization, many of these multisensory interactions occur without our explicit knowledge of any reliable underlying relationship between visual and auditory or haptic information. Recently, intriguing evidence is accumulating to suggest that at least some of these multisensory interactions may depend on direct connections between early sensory cortical areas. I will present some evidence using electrocorticography data from patients with epilepsy to shed light on this speculation.

## Concealed by the Most Salient Structure in Visual Search

**Li Jingling**

Graduate Institute of Biomedical Sciences, China Medical University, Taichung, Taiwan Email: jlli@mail.cmu.edu.tw

## 

Visual search is usually more efficient if the searched target was salient or on a salient location. However, we found a counterintuitive phenomenon that a target is actually more difficult to find if it was placed on a salient collinear structure, which is called the collinear masking effect. We found that this collinear masking effect restricts specifically to perceptual law of good continuity; other salient structures created by feature contrast on orientation, color, or luminance dimensions induced facilitation rather than masking. Also, task requirements, predictability, and practice did not eliminate this collinear masking effect. The collinear masking effect can be observed within 40 ms presentation duration of the search display, suggesting that this effect is majorly based on stimulus-driven computation. Dichoptic presentation showed that the collinear masking effect requests of information from single eye, which implies critical involvement of the primary visual cortex. The data cumulated till now suggest that this collinear masking effect may relate to boundary formation. Our work reveals how object perception links to attentional capture, and in which conditions perceptual grouping can facilitate or impair attention selection.

## Attention at Locations Unattended

**Satoshi Shioiri**

Research Institute of Electrical Communication, Tohoku University, Tohoku, Japan Email: shioiri@riec.tohoku.ac.jp

## 

Attending on a spatial location in the visual field facilitates visual processing at the location, which is important feature for efficient visual processing. While a variety of attentional effects have been studied, basic features such as spatial modulation of attentional effect are still unclear. Particular interest is facilitation and inhibition around focus of attention. Some studies reported facilitation of area adjacent to attention focus, while others reported lateral inhibition of attention effect. We investigated spatial spreads of attentional effect using electroencephalography, analyzing steady-state visual evoked potential (SSVEP) and event-related potential (ERP). SSVEP is a technique to realize measurements of attentional effect at unattended locations with stimuli tagged by temporal frequency and suitable for estimating attentional spreads around focus of attention. We succeeded to measure spatial characteristics of visual attention using SSVEP and compared with ERP measurements. We found that SSVEP showed facilitation around the attention focus, while ERP showed lateral inhibition. We discuss the results in terms of difference in spatial spread of attention at different stages of visual processing.

## Consciousness Without Attention and Large Capacity Conscious Memory, Investigated With Metacognition

**Naotsugu Tsuchiya**

School of Psychological Sciences, Monash University, Victoria, Australia Email: Naotsugu.Tsuchiya@monash.edu

## 

Do we consciously experience only those sensory inputs that we attend to? Or, do we enjoy substantial amount of unattended information in conscious experience? The necessity of top-down attention for conscious perception has been hotly debated in consciousness research. In this talk, we present two studies in which we incorporated trial-by-trial confidence rating to assess “metacognitive accuracy” as a proxy for the degree of conscious access to sensory information. In the first study, we examined how much we remember about the non-target distractor faces in a natural-scene face-search task (Kaunitz, Rowe and Tsuchiya, 2016 PsychSci). In the second study, we examined whether face genders or color orientation of patches in the periphery can be consciously discriminated when they simultaneously perform a highly demanding task at the fixation. In both cases, we found that our memory and perception of faces under inattention are much better than what would be expected if attention is necessary for them and that subjects can access to these information consciously, proven by above chance metacognitive accuracy, consistent with a claim that consciousness without attention is possible and robust for a certain class of stimuli (Koch & Tsuchiya, 2007 Tics).

## Consciousness at a Price: The Attentional Blink Is a Cost of Awareness

**Dominique Lamy**

Department of Psychology, Tel Aviv University, Israel. Email: domi@post.tau.ac.il

## 

Our ability to process successive events occurring in close temporal proximity is limited. In particular, selecting a first event incurs a cost in perceptual processing of a second event. Here, we disentangle the roles of conscious perception of and spatial attention to the first event in eliciting this perceptual cost. We establish the existence of a “cost of awareness” (CoA) and demonstrate that this cost exhibits the core characteristics of the attentional blink (AB), including location specific lag-1 sparing and performance recovery at long lags. Furthermore, we show that conscious perception of the first event is both necessary and sufficient for the perceptual deficit underlying the CoA and the AB to occur, whereas spatial attention to the first event is neither necessary nor sufficient. We thus conclude that the attentional blink is a cost of awareness. The reformulation of the AB as a cost of awareness constrains current models of the attentional blink and highlights the potential role of factors influencing subjective conscious experience in shaping our performance and alleviating limitations of our perceptual system when we interact with successive events.

## Behavioral Influences and Temporal Dynamics of Unconscious Salience Processing

**Po-Jang (Brown) Hsieh**

Duke-NUS Medical School, Brain & Consciousness Lab, Singapore, Singapore Email: pojang.hsieh@duke-nus.edu.sg

## 

Visual popout occurs when a unique visual target (e.g., a feature singleton) is present in a set of homogeneous distractors. However, the role of visual awareness in this process remains unclear. In a series of experiments, we showed that even though subjects were not aware of a suppressed feature singleton, it still can (a) attract subjects’ attention to enhance subsequent performance on an orientation-discrimination task; (b) enables a display to reach awareness faster, and particularly increase a location’s probability of gaining awareness first; (c) influences the direction and latency of saccades. We further demonstrated in an electroencephalography experiment that the presence of a salient feature singleton elicited early event-related potential (ERP) differences during unconscious perceptual processing. Temporally, ERP differences in the frontal electrodes were evident earlier than the occipital electrodes. In addition, alpha oscillatory power was lower when a feature singleton was present. Finally, we demonstrate that the P2 component amplitude in the frontal electrode is associated with both unconscious salience processing and stimulus awareness.

## Attractiveness and Bodily Interactions at Implicit Levels - Reading Social Evaluation from Eyes and/or Bodily Interactions

## Facial Preference, Gaze/Pupil, and Interpersonal Synchrony—How are They Related?

**Shinsuke Shimojo**

California Institute of Technology, Pasadena, CA, USA Email: sshimojo@caltech.edu

## 

My talk will cross-over different domains of research, which have been studied independently— preference/attractiveness of faces, gaze/pupil dynamics, and interpersonal behavioral/neural synchrony. First, preference or attractiveness decision making has been studied with a fixed (either static or dynamic) facial stimuli (Kim et al., 2007; Park et al., 2010). Second, gaze/pupil responses with regard to preference/attractiveness have been studied again with typically fixed facial stimuli (Shimojo et al., 2003; Liao et al., APCV, 2015). This line of research emphasizes dynamic changes of gaze/pupil, yet remains mostly in the framework of stimulus-observer one-way relationship. Third, however, live, two-way interpersonal interactions were studied with interpersonal bodily/neural synchronizations as somatic, implicit bases of social interaction (Yun et al., 2012). These three domains are closely related and progressively coming closer to the real-world natural social communication. I will try to provide research seeds across them; for example, what would happen if we use dynamic pupil change stimulus (though prefixed) with measurement of the observer’s dynamic pupil response, or if we measure pupil responses of two people actually interacting with each other? This will hopefully connect to the other talks in this symposium.

## Dynamics of Attractiveness Judgments

**Katsumi Watanabe^1,2^**

^1^Waseda University, Japan

^2^University of Tokyo, Japan Email: kw@fennel.rcast.u-tokyo.ac.jp

## 

While attractiveness judgments can be done within a short period of time, it does not mean the attractiveness judgment is a simple, one-shot process. In this talk, I will present studies that examine the dynamic aspect of attractiveness judgment. One study concerns judgments of facial attractiveness as a combination of facial parts information over time and investigated how attractiveness judgment of each part of a face (i.e., eye, nose, mouth) would contribute to and be integrated into the attractiveness of the whole face. The results showed that the eyes made a consistently high contribution to whole-face attractiveness, even with an observation duration of 20 ms, whereas the contribution of other facial parts increased as the observation duration grew longer. The other study examined the sequential dependence of attractiveness judgments. Responses in a current trial are biased by the stimulus and response in the preceding trial. We found that attractiveness judgment is also biased toward the preceding judgment, and hence the sequential effect can be extended into the domain of subjective decision making. These studies highlight the dynamic aspect of attractiveness judgments.

## There Is No Hidden Beauty: Unconscious Processing of Facial Attractiveness

**Shao-Min (Sean) Hung^1^, Chih-Hsuan Nieh^2^ and Po-Jang (Brown) Hsieh^1^**

^1^Neuroscience and Behavioral Disorders Program, Duke-NUS Medical School, Singapore

^2^Department of Psychology, National University of Singapore, Singapore Email: shaomin.hung@u.duke.nus.edu

## 

Being appraised as attractive or not plays a pivotal role in one's life, influencing how one is judged, how easily one can be hired after an interview, and even how well an infant could capture his/her mother's attention. However, it remains elusive whether facial attractiveness can be extracted even in the complete absence of visual awareness. Here, we showed unconscious processing of facial attractiveness with three approaches. In Experiment 1, the time taken for faces to enter consciousness under interocular suppression was measured. The results showed that attractive faces broke through suppression and reached consciousness earlier. In Experiment 2, we further showed that attractive faces had lower visibility thresholds under suppression, again suggesting that facial attractiveness could be processed more easily to reach consciousness. Crucially, in Experiment 3, an attractive face, albeit suppressed and invisible, could orient one's attention, resulting in a decrease of accuracy on a subsequent orientation judgment task. This effect disappeared with inverted faces, suggesting that the attentional effect was not driven by local features. Taken together, for the first time, we show that facial attractiveness can be processed in the complete absence of consciousness, and an unconscious attractive face is still capable of directing our attention.

## How Does Pupillary Response Contribute to Interpersonal Preference Evaluation?

**Hsin-I Liao^1^, Ying-Chun Chen^2,3^, Makio Kashino^1^ and Shinsuke Shimojo^2^**

^1^NTT Communication Science Laboratories, Japan

^2^Divison of Biology, California Institute of Technology, USA

^3^Department of Industrial and Commercial Design, National Taiwan University of Science and Technology, Taiwan Email: liao.hsini@lab.ntt.co.jp

## 

Our previous series of studies demonstrated that people’s pupils constrict, but not dilate, when seeing attractive faces (Liao et al., APCV, 2015; TPA, 2016). The result refutes the conventional belief of the positive interpersonal loop between pupil dilation and attractiveness in that a face with dilated pupil was looked more attractive and the pupil of the observer dilated when being attracted (e.g., Hess, 1965). In the current study, we examine the issue by using line-drawing faces with their pupil size manipulated. Participants judged the attractiveness of the faces with different pupil size, while an eye-tracker camera recorded the participants’ own pupillary responses. The pupillary response results replicated our previous finding showing that participants’ pupils constricted stronger when they gave a higher score for the attractiveness judgment. Most importantly, contradictory to Hess’ finding, the behavior result indicated that the line-drawing faces with smaller pupil size appear more attractive. The overall results suggest that there is still positive interpersonal loop between the observer and the actor, but the loop is in the direction of pupil constriction, instead of dilation.

## Decoding Preference Decision Making From Footsteps and Eyes

**Makio Kashino^1,2,3^**

^1^NTT Communication Science Laboratories, Japan

^2^Tokyo Institute of Technology, Japan

^3^JST CREST, Japan Email: kashino.makio@lab.ntt.co.jp

## 

Growing evidence shows that human behavior, decision making, emotion, and communication depend critically on implicit brain functions, that is, automatic, involuntary neural processes even the person herself/himself is not aware of. We have been developing diverse methods to decode implicit brain functions from various data measured from body surface, for example, involuntary body movements, eye dynamics, autonomic nerve activities, and hormone secretion. Here I introduce two topics: interpersonal body synchronization and eye dynamics. First, we developed a computational method to quantify the degree of body synchronization among multiple people and demonstrated that the unconscious synchronization of footsteps between two people who met for the first time and were walking side by side for several minutes enhances positive impressions with each other. Interpersonal impression is, in an aspect, a dynamic phenomenon that emerges through the interaction mediated by bodies. Second, we developed a computational model of eye dynamics based on diverse features, including natural frequency and damping factor of microsaccades, and dilation and constriction of pupils. The model predicts observers’ preference for faces and music. Decoding information obtained from body surface, instead of the brain, provides practical and essential methods to uncover implicit brain functions underlying cognition and to develop user-friendly man-machine interfaces.

## Stopping The Rise of Myopia in Asia

## The Epidemic of Myopia: Prospects for Prevention

**Ian Morgan**

Research School of Biology, Australian National University, Canberra, Australia Email: Ian.Morgan@anu.edu.au

## 

In the developed countries of East and Southeast Asia, myopia has now reached epidemic proportions. In young adults, the prevalence of myopia is 80–90%, while that of high myopia is 10–20%. The increased prevalence of high myopia seems to result from the increasingly early onset of myopia and the faster rates at which myopia worsens during childhood years that have occurred during the emergence of the epidemic. A major risk factor for myopia is education, perhaps mediated by the amount of near-work children perform. In addition, reduced time spent outdoors by children also promotes the development of myopia. The mechanism for the first effect may involve regulation of eye growth by defocus signals generated in the retina, while the latter effect appears due to the regulation of retinal dopamine release by light outdoors. Increased time outdoors in schools has been shown to reduce new cases of myopia. In addition, it is possible to clinically control the rate of worsening of myopia by using atropine eye drops or orthokeratology. Systemic application of these control measures is expected to reduce the prevalence of myopia and high myopia, thus reducing the risk of vision loss due to pathological changes associated with high myopia.

## Light-Induced Dopamine Release Prevents the Development of Experimental Myopia

**Regan Ashby**

Health Research Institute, Faculty of Education, Science, Technology and Mathematics, University of Canberra, Canberra, Australia Email: regan.ashby@canberra.edu.au

## 

Recently, epidemiological studies have shown a clear negative correlation between time spent outdoors and the development of myopia in children, with a number of successful clinical trials already published. It has been postulated that such a protective effect might be mediated by the light-stimulated release of dopamine from the retina, which has been supported by findings from animal studies. Specifically, rearing animals under high illumination levels, relative to that normally seen indoors, significantly retards the development of deprivation myopia in chicks, rhesus monkeys, tree shrews and mice. In chicks, this protective effect is abolished by the administration of the D2 receptor antagonist Spiperone, indicating light dopamine release and D2 dopamine receptors as critical in the pathway. Importantly, in chicks, high light not only prevents the onset of deprivation myopia but it also halts further progression in already myopic eyes. One of the critical implications of these findings is that manipulation of the dopaminergic system can be used to prevent the development of myopia.

## A Novel Wearable Device to Quantify Myopia-Related Behavior Pattern: Analysis of the Data

**Weizhong Lan**

Aier Institute of Optometry and Vision Science, Aier School of Ophthalmology, Aier Eye Hospital Group, Hunan, China Email: 13725458845@139.com

## 

The prevalence of myopia has been increasing steeply in the last decades. However, the underlying pathogenesis is still unclear. Although excessive near-work and lack of outdoor exposure have been generally accepted as myopia-related behaviors, the quantitative correlation between them is inconsistent. One of the major reasons is that conventional approaches (e.g., questionnaire and diary) could only provide overall period-based data (e.g., at most daily), which are not without memory bias. In this presentation, I am going to introduce a novel wearable device that can measure myopia-related behaviors objectively and dynamically. Other than a precise evaluation of the total dose, the device can therefore provide the temporal pattern of these behaviors as well, which was recently found to be a critical independent factor influencing the biological effect. In addition, we have developed an algorithm to describe the myopia-related behaviors, using large data analysis. In a pilot study consisting of 43 students, it is shown that distinct visual behavior patterns exist among students even in the same classroom. It is believed that the technique could offer new insight into the myopia epidemic.

## Successful Attempts to Prevent Myopia in Taiwan

**Pei-Chang Wu**

Department of Ophthalmology, Kaohsiung Chang Gung Memorial Hospital, Kaohsiung, Taiwan Email: wpc@adm.cgmh.org.tw

## 

The Taiwan Student Vision Care Program (TSVCP) promoted by the Ministry of Education has been in effect for three decades in Taiwan. During that time myopia prevalence steadily increased to a high level and therefore research into myopia prevention was given high priority as an important goal of the program. However, in the absence of an evidence-based protective factor, the rise of myopia continued despite much effort to curb it. When it was discovered that the time spent outdoors was an important factor for myopia protection, it was promptly implemented in TSVCP, leading to a breakthrough during the last four years. A nationwide visual acuity screening of elementary school pupils showed that the previously declining vision score (based on uncorrected visual acuity of 20/25 or less) was stopped and reversed. This result strongly supports the “myopia prevention by increased outdoor activities” strategy within TSVCP.

## Contemporary Questions in the Psychophysics of Spatial Vision

## Divisive Inhibition as a Solution to the Correspondence Problem

**Chien-Chung Chen**

Department of Psychology, National Taiwan University, Taiwan Email: c3chen@ntu.edu.tw

## 

To perceive an object in a scene, the visual system needs to integrate local image elements together for a global percept. However, in any sufficiently complex scene, there are multiple possible ways to organize local elements. Hence, it is a challenge for the visual system to find the right correspondence among local elements. For instance, to perceive symmetry, the visual system needs to find correspondence between image elements across a symmetry axis. However, if the location and orientation of the symmetry axis are unknown, the midpoint between any pairs of image elements is a candidate for a symmetry axis. We measured symmetry detection under various contexts and different amount of axis-orientation uncertainty. Our result was best described by a multiple channel model in which each channel tunes to a specific axis orientation. The response of each channel is the number of corresponding elements consistent with the tuned symmetry axis divided by an inhibition signal from other channels. Similar computation principle also found in Glass pattern perception and Ebbinghaus size illusion. Thus, divisive inhibition, which was originally proposed to explain phenomena in the contrast domain, is ubiquitous in perceptual grouping. It serves to suppress unwanted groupings and ensure the emergence of the right ones.

## Early Spatial Vision: A View Through Two Eyes

**Mark A. Georgeson**

School of Life & Health Sciences, Aston University, Birmingham, UK Email: m.a.georgeson@aston.ac.uk

## 

Simple features such as edges are the building blocks of spatial vision. How are visual features and their properties (location, blur, contrast) derived from spatial filter responses in early vision? And how are these visual signals combined across the two eyes? Our psychophysical evidence from blur-matching experiments supports a multi-scale model where edges are found at spatial peaks of response of odd-symmetric receptive fields (gradient operators), and edge blur corresponds to the spatial scale of the most active operator. This model correctly predicts perceived blur for many different luminance profiles, and explains some surprising effects: Blurred edges look sharper at low contrast and at shorter lengths. Binocular combination of early signals certainly involves binocular summation and interocular suppression, but our understanding remains incomplete. Linear summation, for example, predicts that fused edges should look more blurred and lower contrast with increasing disparity, but in experiments edge blur and contrast appear constant across all disparities, whether fused or diplopic. In an effort to unify a variety of effects, I shall describe recent developments in modelling, where binocular summation is highly nonlinear and is shaped by the relative contrasts in the two eyes and where monocular signals may make a direct contribution to perception.

## Curiosities in Spatial Vision

**Tim S. Meese**

School of Life & Health Sciences, Aston University, Birmingham, UK Email: t.s.meese@aston.ac.uk

## 

Spatial vision is one of the great success stories in the computational modelling of psychophysical data in human vision. However, along the way, and following the contributions of many other researchers, I have found several curiosities that remain to be properly understood. These include: (a) the presence of distinct bumps in the handles of dipper functions; (b) a change of dipper handle slopes from 0.6 to 1.00 (i.e., Weber’s Law) when the spatial frequency is low and the temporal frequency is high and (c) curious lawful features of contrast-matching functions which include the implication that spatial filtering is absent (or equivalent to a delta/Dirac pulse) in suprathreshold conditions. I shall discuss these (largely, unpublished) findings and offer some discussion of the possible underlying processes and their implications.

## The Psychophysical Function for Contrast

**Joshua A. Solomon**

School of Health Sciences, City University, London, UK Email: j.a.solomon@city.ac.uk

## 

Accurate derivation of the psychophysical (a.k.a. transducer) function from just-noticeable differences requires accurate knowledge of the relationship between the mean and variance of apparent intensities. Alternatively, the psychophysical function can be inferred from estimates of the average between easily discriminable intensities. Such estimates are unlikely to be biased by the aforementioned variance, but they are notoriously variable and may stem from decisional processes that are more cognitive than sensory. To circumvent cognitive pollution, I used small, densely packed stimuli of varying contrast. Estimates of average intensity became increasingly variable as size and spacing increased, but average estimates of average intensity were always closer to the veridical mean power (i.e., contrast^2^) than they were to the mean contrast or the mean contrast^4^.

## Understanding Individual Differences in Eye Movement Patterns

## Culture Reveals a Flexible System for Face Processing

**Roberto Caldara**

Eye and Brain Mapping Laboratory, Department of Psychology, University of Fribourg, Fribourg, Switzerland Email: roberto.caldara@unifr.ch

## 

The human face transmits a wealth of signals that readily provide crucial information for social interactions, such as facial identity and emotional expression. Nonetheless, a fundamental question remains debated: Is face processing governed by universal perceptual processes? Historically, it has long been presumed that this is the case. However, over the past decade, our work has questioned this widely held assumption. We have investigated the eye movements of Western and Eastern observers across various face processing tasks to determine the effect of culture on perceptual processing. Commonalities aside, we found that Westerners distribute local fixations across the eye and mouth regions, whereas Easterners preferentially deploy central—global—fixations during face recognition. Moreover, during the recognition of facial expressions of emotion, Westerners sample relatively more the mouth to discriminate across expressions, while Easterners the eye region. These observations demonstrate that the face system relies on different strategies to achieve a range of socially-relevant face processing tasks with comparable levels of efficiency. Overall, these cultural perceptual biases challenge the view of universal processes dedicated to face processing, favoring instead the existence of idiosyncratic flexible strategies. The way humans perceive the world and process faces is determined by experience and environmental factors.

## Subclusters of Autistic Traits: Links With Looking at the Eyes, and Face Identity Recognition Ability

**Romina Palermo^1^, Joshua Davis^2^, Marc Zirnsak^3^, Tirin Moore^3^, Richard O’Kearney^2^, Deborah Apthorp^2^ and Elinor McKone^4^**

^1^ARC Centre of Excellence in Cognition and its Disorders, School of Psychology, University of Western Australia, Perth, WA, Australia

^2^Research School of Psychology, The Australian National University, Canberra, ACT, Australia

^3^Department of Neurobiology, and Howard Hughes Medical Institute Stanford University School of Medicine, Stanford University School of Medicine, Stanford, CA, USA

^4^Research School of Psychology, ARC Centre of Excellence in Cognition and its Disorders, The Australian National University, Canberra, ACT, Australia Email: romina.palermo@uwa.edu.au

## 

Autistic traits, as measured with the autism quotient (AQ) (Baron-Cohen et al., 2001), vary across the general population and can be split into subclusters— social aspects (AQ-Social) and non-social aspects (AQ-Attention). These subclusters of autistic traits may have opposite effects on the amount of looking at the eyes of faces, which may differentially affect face recognition ability. We used eye tracking to measure looking time to the eyes of faces, and used regression and mediation to link these with AQ subclusters and face recognition ability. The social and non-social aspects were differentially associated with looking at eyes of faces: AQ-Social was linked with a tendency to reduced looking at eyes, whereas AQ-Attention was associated with increased looking at eyes. Moreover, higher AQ-Attention was then indirectly related to improved face recognition, mediated by increased number of fixations to the eyes during face learning. In contrast, higher levels of AQ-Social were related with poorer recognition of faces. This study highlights the value of distinguishing between different subclusters of traits when attempting to understand the complex links between autistic traits and person perception in the general population and suggests that clinical studies might similarly benefit from considering symptom subclusters.

## Understanding Eye Movement Patterns in Face Recognition Using Hidden Markov Models

**Janet Hsiao**

Department of Psychology, University of Hong Kong, Hong Kong Email: jhsiao@hku.hk

## 

Recent research has reported substantial individual differences in eye movement patterns in visual tasks. Here, we present a hidden Markov model (HMM)-based approach for eye movement data analysis that takes individual differences into account. In this approach, each individual’s eye movements are modeled with an HMM, including both person-specific regions of interests (ROIs) and transitions among the ROIs. Individual HMMs can be clustered to discover common patterns, and similarities between individual patterns can be quantitatively assessed. Through this approach, we discovered two common patterns for viewing faces: holistic (looking mostly at the face center) and analytic (looking mostly at the two eyes). Most participants used holistic patterns for face learning and analytic patterns for face recognition. Participants who used the same or different patterns during learning and recognition did not differ in recognition performance, in contrast to the scan path theory. Interestingly, analytic patterns were associated with better face recognition performance and higher activation in brain regions important for top-down control of visual attention, whereas holistic patterns were associated with ageing and lower cognitive status in older adults. This result suggests the possibility of using eye movements as an easily deployable screening assessment for cognitive decline or deficits.

## Classifying Eye Gaze Patterns and Inferring Individual Preferences Using Hidden Markov Models

**Antoni B. Chan^1^ and Antoine Coutrot^2^**

^1^Department of Computer Science, City University of Hong Kong, Hong Kong

^2^Institute of Behavioural Neuroscience, University College London, UK Email: abchan@cityu.edu.hk

## 

Eye movements can be used to infer characteristics of the observers and what is being observed. However, most of the literature relies on limited gaze descriptors unable to capture the wealth of information contained in these highly dynamic signals. In this talk, we present two methods for analyzing eye gaze patterns using hidden Markov models (HMMs). The first method is for classifying individual's scanpaths. An individual's dynamic gaze behavior is modeled with an HMM. HMM parameters are used to train classifiers to capture systematic gaze patterns diagnostic of the task, the observer's gender, or the presence of soundtrack while watching videos. The second method, called switching HMM (SHMM), extends the HMM to model changes in gaze patterns due to switches in high-level behavior. The SHMM is applied to eye gaze patterns from a preference decision-making task, where it discovers two high-level behaviors: exploration and decision-making. Through clustering individual's characteristic SHMMs, we automatically discovered two groups of participants with different decision-making behavior. The SHMMs were also able to infer participants’ preference choice on each trial with high accuracy. Our approaches make it possible to reveal individual differences in task behavior and discover individual preferences from eye movement data.

## A New World in Primate Vision Research: The Marmoset as A Model Animal

## The Role of Feedback in Early Visual Processing

**Alessandra Angelucci**

University of Utah, UT, USA Email: alessandra.angelucci@hsc.utah.edu

## 

In the primate cortex, information travels along feedforward connections and is in turn modulated by feedback connections from higher to lower order areas. Feedback has been implicated in many important functions for vision; yet, it remains poorly understood. We have used viral and optogenetic approaches to investigate the anatomy and function of feedback connections between the marmoset monkey V1 and V2. We find evidence for the existence of multiple anatomically and functionally distinct feedback channels. Moreover, our results point to a fundamental role of feedback in early visual processing, regulating the visual system sensitivity to image features, by controlling response gain, its ability to localize them in space, by controlling RF size, and increasing their coding efficiency, by increasing surround suppression.

## Complex Visual Processing in Subcortical Visual Pathways

**Natalie Zeater**

University of Sydney, Australia Email: natalie.zeater@sydney.edu.au

## 

It is traditionally thought that complex visual processes (including binocular responses, orientation selectivity and direction selectivity) only begin to emerge at the level of the visual cortex. Here, we describe cells recorded in the marmoset lateral geniculate nucleus and other subcortical nuclei showing cortical-like visual responses.

## Two-Photon Ca Imaging in the Marmoset Cortex

**Tetsuo Yamamori**

RIKEN Brain Science Institute, Japan Email: tetsuo.yamamori@riken.jp

## 

This presentation will cover the RIKEN-developed approach for two-photon Ca imaging in the marmoset cortex using a tTA/Tre amplification system with AAV vectors. It will include recent data in the marmoset visual cortex using two-photon Ca imaging.

## Motion Sensitivity of MT Cells After V1 Lesions

**Maureen Hagan**

Monash University, Melbourne, Australia Email: Maureen.Hagan@monash.edu

## 

Damage to the primary visual cortex (V1) results in a scotoma in the corresponding parts of the visual field. However, both humans and monkeys retain some unconscious visual faculties, or “blindsight”, within the scotoma, presumably via pathways that bypass V1. The motion-sensitive, middle temporal (MT) area is thought to mediate blindsight, as MT neurons respond to stimuli inside the scotoma following V1 lesions. Using moving, random dot stimuli, we found that fewer MT cells were direction selective after V1 lesions compared to control subjects. However, a significant proportion of non-direction cells were tuned to the speed of moving stimuli and titrated their firing rates to the coherence of motion, similar to direction selective cells. This suggests that while direction selectivity is impaired after V1 lesions, motion processing is preserved, even in non-direction selective cells. The decreased proportion of directionally selective cells may explain the compromised global motion perception in these same patients. However, the preserved motion sensitivity in some cells suggests that these cells may be recruited through training to recover global motion discrimination inside the scotoma.

## Motion Estimation in the Common Marmoset

**Jake Yates**

University of Rochester, NY, USA Email: jyates7@ur.rochester.edu

## 

The middle temporal (MT) area of the primate brain plays a causal role in the perception of motion. Studies of MT in the macaque have advanced our understanding of neural population coding and perceptual decision making; however, due to its location inside a sulcus, MT in the macaque is inaccessible to large-scale array recordings and imaging techniques. The common marmoset is a New World primate that shares similar organization of MT, but due to its smooth cortex, it offers unparalleled access to study large populations of neurons. Here, I will describe behavioral performance of two marmoset monkeys performing a continuous motion estimation task. Combined with large-scale recording techniques, this behavioral paradigm offers a new means for studying the neural population code that underlies motion perception.

## Artificial Vision

## Challenges in the Arrival of Prosthetic Vision

**Gregg Suaning**

Graduate School of Biomedical Engineering, University of South Wales, Australia Email: g.suaning@unsw.edu.au

## 

The blind are capturing their first glimpses of what the future may bring in the restoration of sight through prosthetic vision. Clear benefits have been demonstrated, but there remains far to go before so-called 'bionic eyes' can restore more than rudimentary vision. This presentation will discuss the challenges the field faces, and some proposed solutions for overcoming these challenges. Strategies include new stimulation paradigms and device configurations to selectively target specific neuronal elements that may lead to more meaningful perceptions.

## A Smart Electrode for Retinal Stimulator With the Large Number of Stimulus Electrodes

**Jun Ohta**

Graduate School of Materials Science, Nara Institute of Science Technology, Japan Email: ohta@ms.naist.jp

## 

This talk presents a smart electrode for a retinal stimulator with the large number of stimulus electrodes. The smart electrode consists of a stimulus electrode combined with a complementary metal-oxide-semiconductor (CMOS) microchip on a flexible substrate. Since each microchip can turn on and off its associated electrode for stimulation through external control circuitry, only small number of interconnection wires are required. In this presentation, the concept, fabrication process, and experimental results in vitro and in vivo are shown.

## The Design of Subretinal Implant Chip With Shared IrO_2_ Electrodes and Adaptive Background Current Cancellation Techniques

**Chung-Yu Wu**

Biomedical Electronics Translational Research Center, Department of Electronics Engineering, National Chiao Tung University, Taiwan Email: peterwu@mail.nctu.edu.tw

## 

The circuit and system innovation of a photovoltaic cell-powered image sensing and current stimulation chip in 180-nm CMOS Image Sensor (CIS) technology for subretinal implant is presented. In the chip, the infrared (IR) light is incident on photovoltaic cells and pixels, whereas visible light is mainly incident on pixels. The active pixel sensor circuit with the adaptive background current cancellation technique is proposed to cancel the background current generated by the IR on the pixel photodiode. Thus, the current generated by the visible light can be integrated transformed to biphasic stimulation currents. A charge pump circuit is designed to increase power supply voltage and thus injection charges. Under the divisional power supply scheme operation that pixels are turned on individually, the shared electrode scheme is proposed to increase the electrode size. An experimental chip has been designed and fabricated in 180-nm CIS CMOS technology. The chip size is 3 mm × 3 mm. Post-processes are adopted to deposit IrO_2_ on top of the on-chip electrodes. Perylene-c passivation is also used to make the implantable chip biocompatible. Finally, future development on subretinal prosthetic systems is discussed.

## A High-Density and Flexible Imaging Sensor Retinal Prosthesis

**L.-S. Fan^1,2^, Z.-T. Lai^3^, J. Huang^1^, C. Y. Liu^1^, Y.T. Cheng^1^, Y. J. Lai^1^, L. Chiang^1^, H. Chan^1^, J. Hong^1^, Y. C. Chen^1^, Z. Y. Hsiao^1^, J. Huang1, F. Wu^1^, K. Yew^1^, A. Bauquet^1^, M. Sheu^1^, H. Chen^4^, S. Chen^1^, F. S. Hsu^1^, L. J. Lee^4^, C. G. Cheng^1^, C. H. Yang^3,5^, T. C. Chen^3^ and C. M. Yang^3,5^**

^1^Iridium Medical Technology Corporation, Hsinchu, Taiwan

^2^Institute of NEMS, National Tsing-Hua University, Hsinchu, Taiwan

^3^Institute of Electronics, National Tsing-Hua University, Hsinchu, Taiwan

^4^Department of Ophthalmology, National Taiwan University Hospital, Taipei City, Taiwan

^5^School of Medicine, National Taiwan University, Taipei, Taiwan Email: lsfan@mx.nthu.edu.tw

## 

We report a 4,000-pixel high-acuity retinal prosthesis. The retinal prosthesis includes a flexible chip implemented using a 180-nm mixed-signal CMOS Image Sensor technology with a pixel array sensing image and generating bi-phasic electrical stimulations to enable a high visual acuity. This image-sensing retinal prosthesis uses SIROF electrodes 10 µm in size, and the CMOS retinal chip is made into a contact lens shape conforming to the surface of a human eyeball for a better stimulation resolution and a lower stimulation threshold, and it is passivated for long-term biocompatibility. We use in vivo, ex vivo, and in vitro experiments to evaluate the surgery procedure, biocompatibility, thermal and mechanical reliability, and the potential efficacy of the retinal prosthesis. Surgery procedure using mini pigs as the animal model has been verified, and preliminary experiments show the biocompatibility (ISO 10993) of the system. The maximum temperature rise has been verified to be within spec. and an accelerated test of the mechanical reliability of the retinal chip equivalent to operating the retinal prosthesis for ∼10 years under the extreme mechanical stress condition has shown no sign of mechanical failure. In vivo and ex vivo pattern-reversal EEP experiments are used to assess the potential visual acuity of the implanted high-density retinal prosthesis in the subretinal space. The averaged EEP signal amplitude falls within the background signal between the stripe width of 60 m and 30 m. The corresponding visual acuity is 20/250. The same subretinal-patterned stimulations are used in the ex vivo experiments using whole-cell patch clamping to measure the RGC action potential. In the ex vivo experiments, patch clamped RGCs can detect, with correlated spiking, the reversal events at a stripe width of 26 m, indicating a potential stripe resolution of up to 20/120 for this retinal prosthesis.

## Visual Science and Its Outreach to General Public

## Developing Media Workshops for Understanding Human Mind

**Junji Watanabe^1^ and Masami Ikeda^2^**

^1^NTT Communication Science Labs, NTT Corporation, Japan

^2^Department of Psychology, Ochanomizu University, Japan Email: watanabe.junji@lab.ntt.co.jp

## 

Outreach in psychology is aimed to promote the overall well-being of individuals and the common humanity by acquiring a better understanding of the human mind. In the outreach activities, it is crucial to create teaching materials that are directly linked to students' individual experiences. To achieve it, “The Japanese Psychonomic Society Committee for developing teaching materials for high school students” used computer graphics technology. We developed visualization systems called “Face Homunculus Viewer” and “Accidental Resemblance Generator” for providing an opportunity for students to gain a deeper understanding of the relationship between brains and minds. Additionally, we conducted media workshops on human touch perception using FHV and face recognition using AGR. Our contributions are the development of interactive systems for visualizing differences in perception and recognition within each individual and between individuals, and promote the engagement of students in scientific understanding. We believe that this indicates a new direction in science outreach in which computer graphics can be applied.

## Find Out Your Own Face: Data Collection Through Media Workshops

**Shigeo Yoshida**

Graduate School of Information Science and Technology, The University of Tokyo, Tokyo, Japan Email: shigeodayo@me.com

## 

As a member of The Japanese Psychonomic Society Committee for developing teaching materials, we have developed a hands-on workshop “Find Out Your Own Face” with the theme of face memory.

We created an image processing algorithm that can convert one's face into someone else by changing the size and the position of one's facial features (eyebrows, eyes, nose, mouth, and facial contour) in photos. In the workshop, workshop participants answer that which one is the original face of a person (workshop lecturer, other participant, or oneself) among various face photos generated by the image processing algorithm. This workshop aims to promote workshop participants to understand the importance of the positional relationship of facial features about facial memory and the ambiguity of human memory through their experience. Moreover, we have investigated whether human senses coincide with the degrees of converting one's face into someone else, which were presumed in advance, based on the responses of various workshop participants including young and old. In this talk, I will describe the activities of past workshops, outline of the image processing algorithm, the data collected through the workshops, and how to collect them.

## Escape Room Meets Scientific Education: A New Way for Public Scientific Outreach

**ChiaHuei Tseng^1^, Hsin-Ni Ho^2^ and Junji Watanabe^2^**

^1^Research Institute of Electrical Communication, Tohoku University, Japan

^2^NTT Communication Science Labs, NTT Corporation, Japan Email: CH_Tseng@alumni.uci.edu

## 

Escape Room is live-action team-based games where players discover clues, solve puzzles, and accomplish tasks in order to accomplish a specific goal in a limited amount of time. It is an emerging and powerful outreach approach as it requires teamwork, communication, and delegation as well as critical thinking, attention to detail, and lateral thinking. In this talk, we will talk about our attempt to combine gamification and education to introduce Vision and Shitsukan research to the public, as well as the process of developing the escape room game that is run during the APCV conference. This escape room game is in a setting that a mysterious Tanabata festival will be held this year in the holy city Tainan. The players have to solve puzzles related to tactile sensitivity, tactile material recognition, and visual face recognition in order to reawaken the magic power of the Tanabata festival. Through this interactive adventure, we mean to bring players a fun and memorable scientific experience.

## Talk Sessions Attention and Consciousness

## From Cloud to Ground: Designing Accessible Exhibition for Science Communication

**Jui-Chuan Chen and Hsin-Drow Huang**

National Museum of Natural Science, Taichung, Taiwan

## 

Effective, informative, and accessible exhibitions in science museums can promote interactions between visitors and exhibitions and enhance the impression on the visitors, especially when they are designed for interpreting abstract concepts and phenomena hard to observe. In National Museum of Natural Science, we employed accessible exhibits of vision-oriented designs to interpreting biological concepts and phenomena from organismal to molecular scales. The hands-on and mind-on experience created through vision and other sensations reinforce understanding the information we wish to convey. However, since visitors come in with different cultural experiences, their responses and cognitive curiosity towards the exhibitions may vary. A model of experience preference originated from the Smithsonian, named as IPOP, offers four dimensions to approach the visitors’ preferences during the development of exhibition. For the past seven years in NMNS, we explored a variety of approaches for different visitors to meet their expectation. Collected data confirmed that the visitors are responsive and inspired when exhibitions: (a) are designed with ideas (I) for conceptual content or abstract thinking, (b) satisfy people (P) in emotional connections, (c) are enriched with objects (O) of visual language and aesthetics, and (d) provide physical (P) experiences and somatic sensations.

## Paying Attention to Time Is Faster Than Paying Attention to Space

**Yaffa Yeshurun and Shira Tkacz-Domb**

University of Haifa, Haifa, Israel Email: yeshurun@research.haifa.ac.il

## 

This study examined the time course of voluntarily allocation of attention to a specific point in time. We employed the constant foreperiod and the temporal orienting paradigms. With both paradigms, the task was to identify a letter presented for a brief duration (16 ms), preceded by a warning signal. The warning signal was either auditory or visual, and it was either informative—indicating the most likely foreperiod (the interval between the warning signal and the target) or not. Critically, to avoid effects of exogenous temporal attention, both types of warning signal did not include an intensity change. Additionally, unlike previous studies, we included a wide range of foreperiods (25–2,400 ms). In comparison to a non-informative warning signal, identification accuracy was significantly higher when the warning signal was informative. Importantly, such effects of temporal attention were found with both very long and very short foreperiods, suggesting optimal voluntary allocation of attention within 150 ms and up to 2.5 s. Given that letter identification was not speeded, we can conclude that temporal attention improved perceptual processing and that endogenous temporal attention is extremely fast—twice as fast as endogenous spatial attention.

## Keywords

temporal attention, temporal expectations, warning signal

## Acknowledgments

This study was supported by the Israel Science Foundation Grant 1081/13 to YY.

## Effect of Aging on the Collinear Masking Effect in Visual Search

**Li Jingling, Sung-Nan Lai and Yen-Ting Liu**

China Medical University, Liaoning Sheng, China Email: jlli@mail.cmu.edu.tw

## 

In visual search, salient objects usually capture attention. However, our previous studies revealed an opposite phenomenon called the collinear masking effect, which is delayed responses to a target overlapping with a salient collinear object. Aging declines the ability of contour integration, which should decrease the collinear masking effect. Nevertheless, aging increases inhibition mechanism of attention which should enlarge the collinear masking effect. The goal of this study is to test whether the collinear masking effect was weaker or stronger with age. Twenty-eight participants, including 12 old adults (74.1 years) and 16 young adults (23.8 years), were recruited. The search display was composed of nine by nine white horizontal bars, excepting one column vertical (collinear). The target was a tilted line on one of the bars, which overlapped with the collinear column at chance. The participants discriminated the tilted of the target. The RT differences between overlapping and non-overlapping conditions weighted by average RT is the index of collinear masking effect. The results showed that old adults generated 27.63% of collinear masking effect, which is significantly larger than that of young adults (5.46%). Thus, our data support the argument that aging increases inhibition mechanism of attention.

## Keywords

effect of aging, collinear masking effect, visual search, inhibition mechanism of attention

## Acknowledgments

This study was supported by MOST103-2628-H-039-001-MY3.

## Temporal Selection Revisited: What Processes are Disrupted by the Attentional Blink?

**Alon Zivony**

Tel Aviv University, Tel Aviv, Israel Email: Alonzivony@gmail.com

## 

The attentional blink (AB) refers to impaired identification of a target when it follows a previous target within 500 ms. Some theories posit that the AB disrupts attentional control, namely, the process of matching incoming information to the current attentional set. Others propose that it delays the encoding of fully processed information into working memory, and thus posit that semantic processing is intact during the blink. We show that neither theory provides a complete account of the AB. First, we show that a distractor sharing the target’s defining feature captures attention to the same extent whether it appears within or outside the blink, thereby demonstrating that control over the attentional set is unimpaired by the blink. Second, we show that the AB impairs reports of a target’s color to a lesser extent than reports of its identity, although the target’s defining feature is the same in the two conditions. We conclude that (a) the AB disrupts attentional engagement, a process that enables the rapid extraction of complex visual information and (b) the triggering of an attentional episode and attentional engagement are dissociated during the blink, which demonstrates that these are separate processes.

## Keywords

attentional blink, attentional selection, temporal attention, attentional capture, attentional engagement

## Acknowledgments

This study was supported by the Israel Science Foundation (ISF) grant no. 1475/12 to Dominique Lamy.

## Can Gaze Cues Induce Inhibition of Return Under Different Task Demands?

**Syuan-Rong Chen and Li Jingling**

China Medical University, Liaoning Sheng, China Email: jlli@mail.cmu.edu.tw

## 

Usually, our attention shifts toward the locations previously cued. However, if the cue was presented longer than 300 ms, a reverse effect would be observed, which is called the inhibition of return (IOR). In using gaze direction as a cue, the IOR would be delayed to 2,400 ms. Previous studies showed that a more difficult task would delay periphery cued IOR. This study aimed to test whether gaze-induced IOR also altered with task demands. Ninety participants, 30 for each task demand (discrimination, localization, and detection) were recruited. The cue to target onset asynchrony was 1,600 ms to 3,200 ms. The discrimination task was to determine the triangular direction (upward or downward). The localization task was to determine probe position (left or right to the fixation). The detection task asked participants to determine whether a probe appeared. The results showed that IOR was significant at 2,400 ms SOA in all three tasks, and additionally significant at 2,800 ms SOA in the discrimination task. Our data show that gaze cues also generate IOR depending on task demands and delayed IOR when the task was more difficult.

## Keywords

inhibition of return, gaze cue, social attention, task demand, SOA

## Acknowledgments

This study was supported by MOST103-2628-H-039-001-MY3.

## Steady-State EEG Response Correlates of Cross-Modally Facilitated Transitions During Binocular Rivalry

**Naotsugu Tsuchiya^1^, Matthew Davidson^1^, David Alais^2^ and Jeroen van Boxtel^1^**

^1^Monash University, Australia

^2^University of Sydney, Australia Email: naotsugu.tsuchiya@monash.edu

## 

Dissimilar images presented at the same retinal locations to the two eyes induce binocular rivalry. Previously, Lunghi et al. (JNsci 2014) demonstrated cross-modal effects during binocular rivalry between two stimuli with different flicker rates. Here, we aim to investigate the neural correlates of these cross-modal effects using frequency tagging in the EEG. We induced binocular rivalry between two gratings with 4.5 Hz- and 20 Hz-flicker rates while recording 64-channel EEG. Subjects (*N* = 34) either (a) focused on reporting of binocular rivalry only or (b) reported rivalry as a primary task while performing a secondary cross-modal attention task, in which subjects assessed the congruency between auditory/tactile stimuli with their ongoing rivalry percept. In terms of behavior, we found strong cross-modal effects only when subjects paid attention to the cross-modal stimuli and only for low-frequency stimuli. The strength of the cross-modal effect was positively correlated with performance on the cross-modal attention task. In terms of EEG, we found strong effects of attention to auditory/tactile stimuli over fronto–temporo–parietal electrodes. Our preliminary analysis showed correlation between the EEG response and the behavioral cross-modal effects between occipital and centro–temporal areas. Our paradigm offers a promising avenue to explore the neural correlates of cross-modal perception.

## Keywords

binocular rivalry, EEG, cross-modal, attention, flicker

## Acknowledgments

This study was supported by Australian Research Council.

## Continuous Flash Suppression Is Strongly Tuned for Low Temporal Frequencies and High Spatial Frequencies

**David Alais^1^, Shui'er Han^1^ and Claudia Lunghi^2,3^**

^1^School of Psychology, University of Sydney, Australia

^2^Department of Translational Research on Now Technologies in Medicine and Surgery, University of Pisa, Italy

^3^Institute of Neuroscience, CNR, Pisa, Italy Email: david.alais@sydney.edu.au

## 

Continuous flash suppression (CFS) uses rapidly flickering Mondrian patterns in one eye to suppress a target in the other. CFS is widely used to study unconscious visual processes, yet its temporal tuning is unknown. We used spatio-temporally filtered dynamic noise patterns to produce narrow-band maskers and probed the temporal, spatial and orientation tuning of CFS. Surprisingly, CFS suppression peaks very prominently at ∼1 Hz, well below typical CFS flicker rates of 10 Hz, and suppression is greater for high spatial frequencies. Orientation filtering revealed CFS suppression is strongly orientation tuned at low temporal frequencies, but much less so at high frequencies. CFS suppression also increased with masker contrast and size. The observed selectivity for low temporal and high spatial frequencies, and a rising monotonic contrast function, suggest parvocellular/ventral mechanisms underlie CFS suppression, similar to the binocular rivalry, and thus unifies two phenomena sometimes thought to require different explanations. A better understanding of the tuning parameters of CFS suppression will help optimise this popular technique for removing target images from conscious awareness.

## Keywords

continuous flash suppression, awareness, visual suppression

## Acknowledgments

This study was supported by Australian Research Council DP150101731.

## Hacking Into Sleep to Enhance Visuospatial Memory

**Ken Paller**

Northwestern University, Evanston, IL, USA Email: kap@northwestern.edu

## 

Many types of learning take hold gradually and require practice. Yet, when we replay and strengthen memories, we may not realize we are practicing. Memory reactivation during sleep may contribute to the consolidation of memories that produces enduring long-term storage. This reactivation can be investigated via a procedure known as targeted memory reactivation (TMR), in which sensory stimulation is used to modify neural activity while avoiding arousal from sleep. We have found that sounds heard in association with visuospatial learning can be presented again during slow-wave sleep to promote the reactivation of memories. Post-sleep memory testing showed that memory could be systematically and selectively improved. Similar results were obtained with several other types of memory. In some of these experiments, measures of sleep physiology were associated with memory improvement. Brain rhythms can also be entrained during sleep to help understand relevant neurophysiological mechanisms. This research is thus beginning to elucidate critical contributions of sleep to memory consolidation. Furthermore, these methods offer new opportunities for reinforcing learning to enhance clinical outcomes in conjunction with therapies engaged during waking.

## Keywords

visuospatial memory, visual-auditory associations, learning, sleep

## Acknowledgments

This study was funded by NSF (USA).

## Neural Mechanisms

## Increased ipRGC Stimulation Enhances Spatial Contrast Sensitivity at Low Spatial Frequencies in Peripheral Vision

**Sung-en Chien^1^, Akiko Matsumoto^2^, Wakayo Yamashita^2^, Sei-ichi Tsujimura^2^ and Su-Ling Yeh^3,4,5^**

^1^Department of Psychology, National Taiwan University, Taipei, Taiwan

^2^Faculty of Science and Engineering, Kagoshima University, Kagoshima, Japan

^3^Department of Psychology, National Taiwan University, Taipei, Taiwan

^4^Graduate Institute of Brain and Mind Sciences, National Taiwan University, Taipei, Taiwan

^5^Neurobiology and Cognitive Science Center, National Taiwan University, Taipei, Taiwan Email: suling@ntu.edu.tw

## 

A recently discovered third receptor type, intrinsically photosensitive retinal ganglion cell (ipRGC), has attracted much attention because it affects circadian rhythm and sleep via signaling environmental light level. Nevertheless, few studies have examined whether ipRGCs also affect spatial vision. Here, we investigate whether ipRGC stimulation in background affects human contrast sensitivity function (CSF), which varies with background luminance. A Gabor was presented at either left or right side of fixation. Participants were asked to perform a position-judgment task with the two-alternative forced-choice procedure. We measured CSFs at low spatial frequencies in periphery by adopting the silent substitution method to keep background color and luminance silent while manipulating ipRGC stimulation. Three conditions were tested: control, ipRGC-high, and light-flux-high condition. In ipRGC-high condition, only ipRGC stimulation was increased compared to control condition. In light-flux-high condition, both ipRGC and cone stimulations were increased. The results showed that increased ipRGC stimulation enhanced spatial contrast sensitivity, while increased cone stimulation decreased it. Furthermore, increased ipRGC stimulation enhanced a spatial tuning in sensitivity according to changes in shape of the receptive field. Our findings indicate that, contrary to previous findings that ipRGCs contribute mainly to non-visual functions, ipRGCs also contributed to fundamental properties of spatial vision.

## Keywords

spatial vision, intrinsically photosensitive retinal ganglion cell, contrast sensitivity function, peripheral vision

## Acknowledgments

This study was funded by Taiwan Ministry of Science and Technology (MOST 104-2410-H-002-061-MY3 to Su-Ling Yeh and MOST 105-2811-H-002-029 to Sung-en Chien).

## Difference in Brain Activities for Unique and Cardinal Hues Investigated by fMRI

**Ichiro Kuriki, Wakiko Maemura, Kazumichi Matsumiya and Satoshi Shioiri**

Tohoku University, Tohoku, Japan Email: ikuriki@riec.tohoku.ac.jp

## 

Unique hues are important as landmarks of the color appearance, while cone opponent mechanisms are physiologically important for coding colors in lower level of visual system. Considering the analogy with orientation selective neurons, more significant directions (e.g., horizontal and vertical) may be represented differently from others. We compared cortical responses to cardinal and unique hues under isoluminance using functional MRI. Subjects performed either hue-identification or letter memory (two-back) task in a run. Since identification of unique hues possibly employs feedbacks from higher order cortices, larger differences between color and letter tasks are expected with unique hues than with cardinal hues. The result of GLM analysis on whole brain showed no significant difference between unique- and cardinal-hue responses, while comparison between letter- and color-task responses showed significant difference in frontal and occipital cortex. To examine differences in spatial pattern of brain activity between color and letter tasks, a representation similarity analysis using correlation coefficient was applied to V1 and V4 voxels. As a result, unique-hue responses showed significantly larger differences in both visual areas. This result implies that cortical representation of unique hues is affected more by feedback from higher order cortex than cardinal hues.

## Keywords

unique hue, color mechanisms, fMRI, cardinal colors, two-back task

## Acknowledgments

This study was supported by This study was supported by JSPS KAKENHI 15H03460.

## Monocular Orientation-Deprivation in Nature Viewing Strengthens the Deprived Eye

**Yonghua Wang^1^, Jia Qu^1^, Jiawei Zhou^1^ and Robert Hess^2^**

^1^Wenzhou Medical University, China

^2^McGill University, Canada Email: zhoujw@mail.eye.ac.cn

## 

Depriving one specific orientation in two eyes’ viewings, for either a short period of 4 hr or a long period up to four days, has been shown to temporally enhance visual perception to the deprived orientation resulted from the regulation of visual adaptation. Here, we show new evidence that if we conduct the orientation-deprivation monocularly for a short period of 2.5 hr in human adults, the deprived eye rather than the deprived orientation is strengthened in binocular viewing, and this effect could last at least 30 min. Such process is not orientation-selective and cannot be account for by adaptation. Thus, the present results suggest different form of the orientation deprivation-induced visual plasticity in human adults.

## Keywords

visual plasticity, orientation-deprivation, ocular dominance

## Acknowledgments

This study was supported by NSFC 81500754 and QTJ16005 to JZ and the CIHR grants MOP-53346, CCI-125686 and MT-10818 and an ERA-NET NEURON (JTC 2015) to RFH.

## Orientation Tuning in V1 Is Contrast Invariant on Short, but not Long, Timescales

**Masoud Ghodrati, Elizabeth Zavitz, Marcello Rosa and Nicholas Price**

Monash University, Victoria, Australia Email: nicholas.price@monash.edu

## 

Orientation tuning in primary visual cortex (V1) neurons is contrast-invariant; while increasing contrast drives higher firing rates, orientation tuning bandwidths do not change. However, previous studies have not examined the time course of contrast adaptation, and whether stable orientation tuning is maintained as firing rates continually change. We recorded extracellular V1 activity in anaesthetised marmosets using two orientation reverse correlation paradigms, in which gratings with random orientation were updated every 16 ms. In the “slow adaptation” paradigm, four contrasts were tested, but contrast was fixed for 30 min. As shown previously, orientation tuning was contrast invariant. However, our ability to predict orientation from the neuronal responses using a linear decoder increased with contrast.

In the “rapid adaptation” paradigm, luminance and contrast were changed every 5 s. Spiking rates increased immediately after contrast-increments, and then decayed exponentially. Our ability to decode orientation was highest when contrast was high, but surprisingly, within a period of constant contrast, spiking rate changed with no concomitant change in decoding performance. This dramatically demonstrates that adaptation within a few milliseconds allows neurons to encode orientation nearly independently contrast and firing rate; but on longer timescales, orientation encoding is contrast dependent and correlated with mean spiking rate.

## Keywords

V1, contrast, adaptation, orientation

## Acknowledgments

This study was funded by NHMRC, ARC.

## Receptive Field Mapping In the Dorsolateral Frontal Cortex of Marmosets (Callithrix Jacchus)

**Azadeh Feizpour, Declan Rowley, Tristan Chaplin, Piotr Majka, Leo Lui, Nicholas Price, Hsin-Hao Yu and Marcello Rosa**

Physiology Department, School of Biomedical Sciences, Monash University, Victoria, Australia Email: azadeh.feizpour@monash.edu

## 

The marmoset (*Callithrix jacchus*) is emerging as a model for studies in visual neuroscience, but little is known about the frontal visual areas in this species. To date, the location of the marmoset frontal eye field (FEF) has only been suggested based on histology (part of cytoarchitectural areas 8aV and 45), anatomical connections with extrastriate cortex, and limited surface microstimulation. Here, we report on the results of experiments in which high-contrast flashed and moving stimuli were presented to three marmosets anaesthetized under opioid/N_2_O anaesthesia, following implantation of 96-channel ‘Utah’ arrays on the surface of the dorsolateral frontal cortex. The locations of the arrays were correlated with putative cytoarchitectural areas based on post-mortem MRI scan and registration to a marmoset brain template (Majka et al. J Comp Neurol 524: 2161-2181). We found that neurons with clear visual receptive fields were only found in areas 8aV, 8C and 6DR. The latency of the responses was compatible with the findings in macaque FEF. Our data did not show any orientation and direction selectivity in FEF neurons. Our findings provide a firm basis for further studies on the function of these visually responsive regions in the context of attention and visual cognition.

## Keywords

marmoset monkey, Callithrix jacchus, receptive field mapping, frontal eye field, response latency

## Visuotopy and Feature Selectivity of Neurons in the Extrastriate Dorsomedial Area of the Marmoset Monkey

**Hsin-Hao Yu, Declan Rowley, Elizabeth Zavitz, Nicholas Price and Marcello Rosa**

Monash University, Victoria, Australia Email: hhyu00@gmail.com

## 

The organization of the “third-tier" areas (Brodmann's area 19) in the primate visual cortex has been an issue of controversy. While the traditional view is that in small New World monkeys, adjoining the rostral border of dorsal V2 is the dorsomedial (DM) area, representing both the upper and the lower quadrants of the visual field (Allman & Kaas, 1971), some researchers proposed a macaque-like area V3 representing only the lower quadrant. We densely mapped the visuotopy in this cortical region of the marmoset monkey, using 10-by-10 multi-electrode arrays and were able to precisely duplicate the DM-map suggested by Rosa & Schmid (1995). Furthermore, we used white noise analysis to quantitatively model DM neurons' response characteristics and showed that their responses could be approximated by small numbers of filters resembling Gabor functions, similar to the orientation-selective complex cells in V1. Finally, penetrations throughout the medial segment of DM showed that although most DM neurons in the dorsal surface of DM are not direction selective, direction selectivity increases with eccentricity in DM. The results are consistent with the idea that DM is an intermediate-level area mediating functions related to both the dorsal and the ventral pathways.

## Keywords

electrophysiology, primate, extrastriate visual cortex, brain mapping

## Acknowledgments

This study was funded by Australian Research Council (ARC), National Health and Medical Research Council.

## Visual Responses of Primate Orbitofrontal Neurons Contribute to Preference Judgment

**Shintaro Funahashi**

Kyoto University, Kyoto, Japan Email: funahashi.shintaro.35e@st.kyoto-u.ac.jp

## 

Human neuroimaging studies have shown that the magnitude of orbitofrontal responses to neutral visual stimuli correlates with the strength of the preference for these stimuli obtained in behavioral studies. In the present study, we examined whether or not the magnitude of orbitofrontal single-neuron responses to neutral visual stimuli correlates with the strength of the preference for these stimuli observed in the behavioral study. First, we determined the strength of the preference (rank order) for 50 neutral visual stimuli behaviorally using two monkeys, and then examined correlations between the magnitude of orbitofrontal response to each of these stimuli and the behaviorally determined preference rank order of the stimulus. Among 188 neurons recorded, 65 exhibited responses to visual stimuli and exhibited stimulus selectivity. One-third of these neurons exhibited either positive or negative correlations between the magnitude of visual responses and preference rank orders of visual stimuli. These results suggest that orbitofrontal neurons participate in the judgment of the preference for neutral visual stimuli. Since the orbitofrontal cortex is known to participate in the estimation of the value of the stimuli as a reward, preference judgment for neutral stimuli may correspond to this mechanism.

## Keywords

monkey, orbitofrontal cortex, visual response, preference, single-neuron activity

## Acknowledgments

Grants-in-Aid for Scientific Research was obtained (25135721, 25240021) from MEXT Japan.

## Space, Time and Motion

## Stimulus Structure Impacts Population Codes for Motion Within and Between Visual Areas V1 and MT

**Elizabeth Zavitz, Maureen A. Hagan, Marcello G. P. Rosa, Hsin-Hao Yu, Leo L. Lui and Nicholas S. C. Price**

Monash University, Victoria, Australia Email: elizabeth.zavitz@monash.edu

## 

The middle temporal area (MT) computes motion direction based on the inputs it receives from direction-selective neurons in primary visual cortex (V1). While V1 neurons signal the direction of ‘local’ moving edges, MT neurons compute a ‘global’ direction that is consistent for many types of spatial patterns. We do not know how information from populations of V1 neurons is combined to produce the selectivity observed in MT. To examine how visual information is successively represented by V1 and MT, we used separate multi-electrode arrays in each area to measure neural responses simultaneously from dozens of neurons in anaesthetised marmosets. We recorded neural activity while presenting motion with different kinds of visual patterns: fields of dots, sine waves and square waves. All of these patterns evoke strong motion signals in MT, but recruit distinct V1 populations. To examine how the V1 representation related to the representation produced in MT, we measured tuning of individual neurons, receptive field overlap and the depth of the MT neurons. We then related these quantities to noise correlations in the populations within and between areas for different visual patterns. These results will tell us more about how information is transformed between brain areas to produce perception.

## Keywords

motion, MT, marmoset, V1, image structure

## Acknowledgments

This study was funded by NHMRC Australia and the Australian Research Council.

## The Spinner Illusion and the Effect of Harmonic Components

**Hiroshi Ashida^1^, Alan Ho^2^, Akiyoshi Kitaoka^3^ and Stuart Anstis^4^**

^1^Kyoto University, Japan

^2^Ambrose University, Canada

^3^Ritsumeikan University, Japan

^4^University of California, San Diego, CA, USA Email: ashida@psy.bun.kyoto-u.ac.jp

## 

The spinner illusion is an academic form of ‘The Coyote Illusion’ (Ho & Anstis, 2013 Best Illusion of the Year contest), demonstrating that moving stimuli appear faster with higher spatial frequencies when the physical speed is constant (Ashida, Ho, Kitaoka, and Anstis, 2017, i-Perception). One problem is that the illusion persists up to 16 elements per revolution with the original ring of spots while the effect almost saturates at around eight cycles per revolution (c/rev) with sinusoidal gratings. The reason for this discrepancy could be that the spot stimulus has sharp edges that introduce harmonic components of higher spatial and temporal frequencies. We therefore measured the spinner illusion in radial gratings of 8 c/rev versus 16 c/rev, with either sine-wave (no harmonics) or square-wave (having odd spatial harmonics) modulation of luminance. We found that the square-wave stimuli yielded a larger effect of speed overestimation for 16 c/rev than the sine-wave stimuli. This difference could be mainly attributed to the observation that 8-c/rev square-wave stimulus was perceived slower than the 8-c/rec sine-wave stimulus. These results, which are qualitatively consistent with Brooks, Morris, and Thompson (2011, J. Vis.), demonstrate a crucial role of harmonic components in speed perception.

## Keywords

visual illusion, speed perception, spatial frequency

## Acknowledgments

JSPS Grant-in-Aid for Scientific Research (KAKENHI) #26285165 for HA, #15H01984 for HA and AK, a grant from the UCSD Department of Psychology for SA and a professional development grant from the Ambrose University for AH.

## Illusory Motion at the Photoreceptor Level: Insights From a Computational Model of Visual Transduction Dynamics

**Gert van Tonder^1^ and Hiroshi Ashida^2^**

^1^Indenpendent Researcher

^2^Kyoto University, Japan Email: ashida@psy.bun.kyoto-u.ac.jp

## 

Visual transduction at photoreceptor level in the retina is characterized by temporal dynamics that effectively implies spatio-temporal filtering at the front-end of vision, particularly enhanced for moving visual stimuli. A range of patterns that induce apparent motion, including the 'rotating snakes' and 'Ouchi' illusions, are known to elicit strong effects under at least minimal eye movements and with a constellation of visual features that act as motion 'triggers'. Here, we revisit the Ouchi illusion to show how a computational model of visual transduction in a generic cone responds to different motion trigger features. Motion blur patterns generated by the model suggests that moving orientation, contrast and spatial frequency gradients result in motion blur that could potentially cause a subsequent edge detection model to induce a 'false' relative spatial displacement between elements across a feature gradient—corresponding to the experienced apparent motion experienced by a human subject. As a windfall, the model also generates output intensity maps that partially correspond to transitory contrast—or scintillating 'illusory contrast'—patterns experienced by human subjects viewing stimulus patterns, such as the Ouchi pattern.

## Keywords

illusory motion, apparent motion, visual transduction, Ouchi Illusion, motion blur

## Acknowledgments

This study was funded by Kyoto University, Faculty of Letters.

## Impaired Sensitivity in Recognizing Biological Motions and Goal-Intentions in Patients With Parkinson’s Disease and Dementia

**Mary Wen-Reng Ho^1^, Shu-Fei Yang^2^, Chun-Man Chen^1^, Chon-Haw Tsai^3^, Hsien-Yuan Lane^4,5^ and Sarina Hui-Lin Chien^6,7^**

^1^Graduate Institute of Biomedical Sciences, China Medical University, Taichung, Taiwan

^2^Graduate Institute of Neural & Cognitive Sciences, China Medical University, Taichung, Taiwan

^3^Department of Neurology, China Medical University Hospital, Taichung, Taiwan

^4^Graduate Institute of Biomedical Science, China Medical University, Taichung, Taiwan

^5^Graduate Institute of Clinical Medical Science, China Medical University, Taichung, Taiwan

^6^Graduate Institute of Biomedical Science, China Medical University, Taichung, Taiwan

^7^Graduate Institute of Neural & Cognitive Sciences, China Medical University, Taichung, Taiwan Email: sarinachien@mail.cmu.edu.tw

## 

Parkinson’s disease with dementia (PDD) is a progressive degenerative brain disease leading to severe deficits in motor and mental capabilities. Perceiving and interpreting body movements comes easily for humans, even when depicted by point-light-display (PLD). Our main question is, if patients with PDD are incapable of executing movement, are they also unable to comprehend movements? We tested eight patients with PDD (4 females; mean age: 76.12) and five healthy controls (3 females; mean age: 63.6), with biological motion-recognition and goal-intention-task. The biological motion-task contained 12-PLD clips depicting human motions (i.e., jumping-jack), and each was displayed three times. The goal-intention-task contained four films; each has a familiarization (participants were to answer which stuffed-animal is grabbed) and a test (with the model’s hand raised in the center and paused). Participants were asked “What will she do next?” and “Which will she grab?” In the biological motion-task, the healthy controls had an average of 10.2 (*SD* = 1.30) correct answers out of 12, while the PDD group performed poorly with only 2.25 (*SD* = 2.25). In the goal-intention-task, both groups showed consistent answers in the familiarization, but the PDD patients responded unpredictably in the test. In sum, our preliminary finding suggests that patients with PDD have difficulty in comprehending movement.

## Keywords

Parkinson's disease with dementia, biological motions, point-light display, goal intentions, human movement

## Acknowledgments

This study was funded to Dr. Chien (MOST 103-2410-H-039-002-MY3, MOST 105-2420-H-039-001-MY3, MOST 105-2632-B-039-003).

## Serial Dependence in Interval Timing

**Huihui Zhang^1^, David Alais^1^ and Xiaolin Zhou^2^**

^1^School of Psychology, The University of Sydney; Australia

^2^School of Psychological and Cognitive Sciences, Peking University, China Email: davida@psych.usyd.edu.au

## 

Visual perception is serially dependent, influenced by immediate past experience. Here, we examined whether time perception was susceptible to the recent history of temporal information. Participants were required to reproduce time intervals (810–1,200 ms) in either a unisensory or a multisensory context. In unisensory tasks, sample intervals were presented in a single visual or auditory modality; in multisensory tasks, half the intervals (those of shorter length) and the other half (intervals of longer length) were presented in different modalities, visual and auditory. We found that reproduced times were biased towards the mean of the distribution of time intervals in both unisensory and multisensory tasks, suggesting that participants’ timing was influenced by the prior distribution of time intervals. In addition to this classic central tendency effect, we also found positive serial dependencies in both unisensory and multisensory tasks. However, for the multisensory tasks, further analysis showed that the positive serial dependencies only appeared if the previous trial and the current trial came from the same modality. Our findings suggest that two types of past experience influence current timing: A long-term prior which is represented in a supramodal manner and a short-term serial dependence bias is exclusively unimodal.

## Keywords

time perception, central tendency, serial dependence, multisensory

## Time Stays Still Under Blue Light: Subjective Time Expansion With Increased Stimulation Level of Intrinsically Photosensitive Retinal Ganglion Cells

**Pei-Ling Yang^1^, Sei-ichi Tsujimura^2^, Akiko Matsumoto^2^, Wakayo Yamashita^2^ and Su-Ling Yeh^1^**

^1^Department of Psychology, National Taiwan University, Taiwan

^2^Faculty of Science and Engineering, Kagoshima University, Japan Email: suling@ntu.edu.tw

## 

Light plays an important role in modern society as it affects both image-forming and non-imaging-forming function. Here, we investigated the effect of blue light on time perception, one of the most important cognitive functions maintaining essential social interactions. Previous studies on this issue rendered inconsistent results; here, we re-examine it using an oddball paradigm which is more sensitive than production and reproduction paradigms with a systematic manipulation of lights. In the oddball paradigm, participants were asked to judge the duration of the target, compared to that of the standard. With the condition of either blue or red background light in Experiment 1, participant’s time perception was lengthened with blue light. Experiment 2 further clarified the contribution of a recently discovered type of retinal ganglion cells that are intrinsically photosensitive (ipRGCs) which is especially sensitive to blue light, with a multi-primary stimulation system that can increase the stimulation of ipRGCs with a metameric background. Results showed that increased stimulation of ipRGC lengthened time perception. These results suggest that blue light expands subjective duration mainly through the contribution of ipRGCs. These results shed lights on further investigations of how ipRGCs affect the timing mechanism and future applications in media and lighting designs.

## Keywords

blue light, time perception, intrinsically photosensitive retinal ganglion cells, oddball paradigm, psychometric functions

## Acknowledgments

This study was funded by Ministry of Science and Technology, R.O.C. (MOST 104-2410-H-002-061-MY3).

## Modeling the Learning Process of Object Locations in Natural Scenes

**Satoshi Shioiri, Zhengxiong Yuan, Kazumichi Matsumiya and Ichiro Kuriki**

Tohoku University, Tohoku, Japan Email: shioiri@riec.tohoku.ac.jp

## 

The visual system constructs representations of the world through repeated observations as suggested by contextual cueing effect (CCE). CCE is the learning effect of spatial layouts revealed by reaction time shortening in visual search due to repeating presentations of the same layouts. We built a model that predicts reaction time shortening by CCE, using reinforcement learning of relationships between the target location and global features of layouts. The relationship is expressed by mapping probability of the target location on each layout. The probability map is used to weigh the saliency map obtained based on visual features in order to predict where to attend for searching a target. With successful learning, the probability map is expected to show the largest probability at the actual target location. The learning speed depends on a parameter of reinforcement and we obtained the parameter for the best prediction of psychophysical experiments. The CCE was measured either with typical letter stimuli or with natural scenes. The model simulation showed that the larger reinforcement effect is required with natural scenes than with letter stimuli. This may suggest that the visual system is designed to learn natural scenes.

## Keywords

visual learning, visual attention, contextual cueing effect, model

## Acknowledgments

This study was supported by Grant-in-Aid for Scientific Research (16H01659).

## Objects and Forms

## The Developmental Trajectory of Susceptibility to Optical Illusions

**Philippe Chouinard^1^, Kayla Royals^1^, Oriane Landry^1^, Sheila Crewther^1^ and Irene Sperandio^2^**

^1^LA Trobe University; Australia

^2^University of East Anglia, UK Email: p.chouinard@latrobe.edu.au

## 

One hundred and three children aged 6 to 15 years judged stimuli on 13 illusion displays. Correlations examined relationships between age, PPVT, and RPM to susceptibility. Five illusions showed developmental change. The Shepard Tabletop showed positive correlations to all three indices; partial correlations could not tease these apart. The Helmholz square showed positive correlations to all three indices; partial correlations showed a correlation with RPM when controlling for age (*r* = .20, *p* = .044). The Poggendorf showed negative correlations to all three indices; partial correlations showed that age was correlated with susceptibility to this illusion above and beyond PPVT (*r* = −.36, *p* < .001) and RPM (*r* = −.36, *p* < .001), whereas neither PPVT nor RPM were correlated with susceptibility when age was controlled. The Jastrow showed negative correlations with both age and PPVT; partial correlations showed that age was correlated with susceptibility when PPVT was controlled (*r* = −.28, *p* = .004), but PPVT was no longer correlated with susceptibility when age was controlled. Finally, the Fick was only correlated with RPM (*r* = .26, *p* = .009). We conclude that illusions follow different developmental trajectories and cannot be regarded as a singular construct in perceptual development.

## Keywords

vision, optical illusions, development, perception

## Effect of Display Density on the Collinear Masking Effect in Visual Search

**Yen-Ting Liu and Li Jingling**

China Medical University, Shenyang, China Email: jlli@mail.cmu.edu.tw

## 

Collinear masking effect (CME) is the prolonged responses to a target overlapping with a collinear structure compare to non-overlapping condition. Since perceptual grouping strength would increase with display density, we assumed that the CME, which associates with grouping strength of collinearity, should also increase with display density. To measure the size of the CME and to keep search display comparable, we designed three search displays with fixed 9 by 9 elements but extended 11.84° × 11.84° (the highest density), 17.76° × 17.76° (the middle density), and 26.64° × 26.64° (the least density) in visual angle. The target and collinear structure could appear randomly at three possible locations in the display, and the overlapping probability of target and collinear structure was at chance level. A prolonged RT to overlapping trials to non-overlapping trials is defined as the CME. The results showed that the size of CME in the highest density condition (50 ms) was significantly higher than that for the middle (29 ms) and the least (26 ms) density conditions. Our study replicated that the CME is larger when the collinear grouping strength is stronger, showing that collinear grouping is the main causes of the CME.

## Keywords

collinear masking effect, display density, perceptual grouping, visual search, collinear grouping

## Acknowledgments

This study was supported by MOST103-2628-H-039-001-MY3.

## Visual Phantoms Induced by Contrast-Modulated Plaids

**Kenzo Sakurai**

Tohoku Gakuin University, Sendai, Japan Email: sakurai@mail.tohoku-gakuin.ac.jp

## 

When luminance-modulated gratings move behind an opaque black occluder, the gratings appear to be continuous on the occluder. This surface completion illusion called visual phantoms disappear as the occluder’s luminance is close to the mean luminance of inducing gratings. Contrast-modulated patterns, however, induce a different type of visual phantoms even when the occluder has a mean luminance of inducing patterns. I investigated whether visual phantoms could be induced by contrast modulation of stationary plaids, and whether the visibility of the phantoms induced by moving contrast modulation would be higher than that of phantoms induced by stationary contrast modulation. Participants viewed plaids oriented 45° with horizontal occluder which luminance was varied, then they rated the visibilities of the visual phantoms with stationary or moving contrast modulation. Results showed that faint illusory plaids were observed on the occluder, and their visibilities were higher when the contrast modulation was moving than when it was stationary. These results suggest contributions of second-order mechanisms to the perception of visual phantoms.

## Keywords

visual phantoms, plaids, contrast modulation

## Acknowledgments

JSPS Grant-in-Aid for Scientific Research (c) Grant Number 17K04498.

## Do Different Patterns of Orientation Change Influence Performance in Texture Segmentation and Detection Tasks?

**David Keeble and Shumetha Sidhu**

University of Nottingham Malaysia Campus, Semenyih, Malaysia Email: David.Keeble@nottingham.edu.my

## 

Orientation gradients are thought to play a fundamental role in orientation-based texture segregation. Studies have shown that segregation can occur when there is an abrupt change in orientation across space, that is, a texture edge, but also in their absence. Here, we investigated the role edges play in the segregation process. We measured participants’ performance to segment and detect rectangular line texture figures of differing mean orientation from the background at five stimulus durations. The orientation change of figure from background was abrupt (Block), or varied spatially according to Cornsweet profile (Cornsweet) or a logistic curve (Blur). Performance at three, values of orientation jitter was also measured. As a function of edge contrast (orientation contrast at the edge), the Blur profile had the lowest threshold, followed by the Block, then Cornsweet. When plotted as a function of center contrast (orientation contrast between background and center of figure), the Blur profile had the highest thresholds. We also found higher thresholds for the segmentation task compared to the detection task (especially for the Blur profile), and higher thresholds with increased orientation jitter and reduced display duration. Therefore, texture properties over regions beyond the edge play a role in the segregation process.

## Keywords

texture, spatial vision, psychophysics

## Size Statistics of the Background Texture Modulates Perceived Target Size

**Chia-Ching Wu^1^ and Chien-Chung Chen^2^**

^1^Fo Guang University; Taiwan

^2^National Taiwan University, Taiwan Email: ccwu@mail.fgu.edu.tw

## 

We investigated the effect of the statistics of background element size distribution on the perceived size of a target. We manipulated the first-, second-, and third-order statistics (or mean, variance, and skewness) of the background element size distribution. We used a two-interval forced-choice paradigm to measure perceived target size at different background size distributions. In each trial, the standard disk, or target, with a texture background texture was presented in one interval while a comparison disk on a blank background, the other. The task of the observers was to determine which interval contained a larger disk. We measured the point of subjective equality (PSE) for the perceived target size with a staircase procedure. The perceived target size decreased with mean background disk size. The variance and the skewness of the background element size did not affect the perceived target size. Our results showed that only the first-order statistics, but not the second-order statistics of the background modulates the perceived target size. We proposed a neural-based model, in which the visual system extracts size information by averaging the responses of different spatial frequency channels whose response is modulated by background element size, to account for our results.

## Keywords

perceived size, Ebbinghaus illusion, context effect, size averaging

## Acknowledgments

This study was supported by MOST104-2410-H-431-007-MY2.

## Psychophysically-Based Enhancement of Features in Medical Images

**Juno Kim and Maria Markoulli**

University of New South Wales, NSW, Australia Email: juno.kim@unsw.edu.au

## 

Textures are changes in surface reflectance that generate edge contours in images independently of the shading attributed to shape and illumination. Recent work has shown that the perception of texture contrast varies with the orientation of reflectance boundaries relative to the direction of shading gradients (Kim, Marlow and Anderson, 2014); texture gradients appear to have greater contrast when they run orthogonally to the underlying shading gradients. The perceived contrast of textures was also found to be predicted by orientation field models that compute the change in direction of gradients adjacent to texture contours. We explored the usefulness of computing the difference in local orientation field responses for analysing textures in medical images—the structure of subbasal corneal nerve fibres imaged using in vivo confocal microscopy. We computed the local difference in orientation field responses across the image and attenuated luminance as a function of these differences. The assumption here was that orientation fields should be similar along nerve contours, but different along an orthogonal axis. We found this structure enhancement technique improved texture segmentation and accounted for perceptual judgments of nerve density, suggesting that orientation fields provide diagnostic support for the automated visual analysis of biological textures in clinical scenarios.

## Keywords

texture, enhancement, perception, nerve density, confocal microscopy

## Acknowledgments

This study was supported by Australian Research Council (ARC) future fellowship to JK.

## Face Perception

## Face Matching Requires Holistic Processing: Evidence From a Gaze-Contingent Task

**Alejandro J. Estudillo**

University of Nottingham Malaysia Campus, Semenyih, Malaysia Email: alejandro.estudillo@nottingham.edu.my

## 

Matching unfamiliar faces is an important task in security and forensic settings, but research has shown that it is error prone. This study uses a gaze-contingent paradigm to explore the efficacy of focusing on isolated features during face matching. A pair of faces was presented at the same time and observers had to indicate whether they depict the same identity or two different identities. In the windows condition, only a single fixated facial feature was available at a time. In the mask condition, the fixated facial feature was masked, while making the rest of the face visible. In the control condition, full faces were presented. Results showed that observers were better in the control condition than in the windows or mask condition. Furthermore, performance in the mask condition was significantly better than in the windows condition. These data showed that focusing on isolated features does not help face matching.

## Keywords

face matching, holistic processing, gaze contingent, featural processing

## Exploring Taiwanese Young Children’s Perception and Categorization of Racially Ambiguous Faces

**Chun-Man Chen^1^, Sarah Gaither^2^ and Sarina Hui-Lin Chien^3^**

^1^Graduate Institute of Biomedical Sciences, China Medical University, Taichung, Taiwan

^2^Department of Psychology & Neuroscience, Duke University, Durham, NC, USA

^3^Graduate Institute of Neural & Cognitive Sciences, China Medical University, Taichung, Taiwan Email: mamy5223@gmail.com

## 

Other-race effect (ORE) refers to that people recognize or memorize own-race faces better than other-race faces. Although ORE is reliably demonstrated across ethnicity, biracial faces are rarely explored. We aimed to explore the effect of essentialist thinking on perceiving and categorizing racially ambiguous faces in Taiwanese young children. Sixty three to six-year-old children and 30 adults performed categorization of biracial individuals’ faces in two conditions: Asian/White (own- and other-race) and Black/White (both other-race) biracial faces. In each condition, children performed three tasks: the online Categorization Task and the Crayon Task, and the Constancy Task (to divide children as essentialist/non-essentialist thinker); adults performed the online Categorization Task only. About one-third of children employed essentialist thinking on race. For the Asian/White condition, adults and the children with essentialist thinking (*N* = 22) tended to categorize the ambiguous faces as White (other-race), whereas the children with non-essentialist thinking (*N* = 38) categorized the ambiguous faces to both races evenly. This observation is consistent with the previous study with White children. For the Black/White condition, all the participants tended to categorize the biracial faces as White, which is a novel finding. The present study provided cross-cultural evidence exploring the effect of essentialist thinking on children’s perception and categorization of racially ambiguous faces.

## Keywords

racial categorization, biracial perception, cognitive development, essentialist thinking

## Acknowledgments

This study was funded to Dr. Chien (MOST 103-2410-H-039-002-MY3, MOST 105-2420-H-039-001-MY3, MOST 105-2632-B-039-003).

## Bilingualism Shapes Face and Music Perception in Developmental Prosopagnosia

**Edwin Burns, Alice H. D. Chan and Hong Xu**

Nanyang Technological University, Singapore, Singapore Email: eburns@ntu.edu.sg

## 

Face memory processes are thought to be largely domain specific. By contrast, music and language perception are driven by processes linked to domain general aspects of face perception, including the own race bias, that is, better discrimination of your own race’s faces over others. We tested these hypotheses in a series of tasks using developmental prosopagnosia cases, who suffer from lifelong impairments in face memory and matched controls. In our controls and DP cases, we found that increasing bilingual proficiency diminished the own race bias, that is, better recognition of your own race faces over others. We also found further links between language, music and face perception that were apparent in our DP cases, but not our controls. By contrast, face memory performance in itself was not related to language or musical ability across all participants. These findings indicate that prosopagnosia cases can be useful in highlighting domain general links between face, music and language perception that are typically obscured in those with intact face recognition abilities. We propose that the superior temporal sulcus is the most likely neural region linking these domains. Our findings have important theoretical implications for current models of face perception and social cognition.

## Keywords

prosopagnosia, language, face, music, bilingualism

## The Magical Number 10 in Face Recognition

**Daisuke Matsuyoshi^1,2,3^ and Katsumi Watanabe^1,2^**

^1^Waseda University, Japan

^2^The University of Tokyo, Japan

^3^Araya Inc., Japan Email: matsuyoshi@aoni.waseda.jp

## 

Despite accumulating evidence pointing a massive long-term memory capacity for objects and places, little is known about the capacity limit for faces. Here, we report behavioral experiments that demonstrate highly limited but efficient memory mechanisms for human faces in a large population (*N* > 900). Participants viewed pictures of various faces and objects one at a time. Afterward, they were shown an image and asked to indicate whether they had seen it. Although the memory capacity for objects drawn from distinct categories was actually massive, that for faces was only 10 and was not affected by the number of items to-be-remembered and race of the face. Nevertheless, face recognition performance was superior to the recognition performance for single-category items with comparable physical characteristics (in terms of isometry, topology and entropy). These results indicate that it is orientation-dependent tuning to typical human-face (geometric) morphology that confers an advantage for the limited but efficient memory storage for faces—an object category with subtle visual differences amongst themselves. Our findings not only pose a major challenge for long-term memory models in which the storage capacity is almost limitless but also necessitate a reevaluation of face recognition models, which have long presumed own-race-biased expertise.

## Keywords

face recognition, capacity limit, geometry, topology, entropy

## Acknowledgments

This study was supported by JST CREST JPMJCR14E4.

## Brain Activities in Face-Selective Regions Predict Performances on Face Recognition and Memory

**Gary C.-W. Shyi, Peter K.-H. Cheng, Varden C.-S. Hung, Becky Y.-C. Chen and S.-T. Tina Huang**

National Chung Cheng University, Chiayi, Taiwan Email: cwshyi@gmail.com

## 

Face recognition and memory entail not only encoding the perceptual input of a face upon its presence but also retrieving a relatively permanent representation in spite of variations in illumination, pose, or expression. A network of face-selective regions has been identified as the core system of face processing, including OFA, FFA, and pSTS. Moreover, recent studies have proposed that ventral route of face processing should end at the anterior temporal lobes (vATLs), which may play an important role in connecting face perception and memory. Here, we examined whether neural activity in the core system and vATLs can predict performance on face recognition and memory. We first identified during the functional scan the core face network by asking participants to perform a one-back task, while viewing either static images or dynamic videos. They then performed a variety of tasks tapping face recognition and face memory. Results revealed that participants with greater BOLD signals in FFA and vATL demonstrated better performance on holistic processing. Furthermore, greater stability in creating face representation in the right vATL exhibited better performance on face memory. These findings suggested individual differences in the generation of invariant face representation can predict behavioral performance on face recognition and memory.

## Keywords

face recognition, face memory, holistic processing, functional brain imaging

## Acknowledgments

This study was supported by Ministry of Science and Technology, Taiwan, ROC.

## Confusion Between Disgust and Anger! The Problem Stems From the Upper Part of a Face!

**Li-Chuan Hsu^1^, Yu-Pei Lin^2^, Yi-Min Tien^2^ and Chia-Yao Lin^3^**

^1^School of Medicine, Graduate Institute of Biomedical Sciences, China Medical University, Shenyang, China

^2^Department of Psychology, Chung-Shan Medical University, Taichung, Taiwan

^3^School of Medicine, China Medical University, Shenyang, China Email: lichuanhsu2001@gmail.com

## 

The degree of distinctiveness between emotions is not uniform. A disgust face is often recognized as an angry face. We aimed to investigate why this confusion occurs. We confirmed previous studies that participants were more likely to confuse disgust with anger while asking them to judge the category of the facial expressions (Experiment 1). We adopted affective priming paradigm in which a 33 ms prime face was presented and found an angry prime would facilitated participants’ performance to judge a disgust target face (Experiment 2). This priming effect was also shown when the prime was an upper-half angry face (Experiment 3A); but however, no such effect was found in lower-half condition (Experiment 3B). When we increased the presented time of a prime 33 ms to 100 ms, the priming effect was eliminated (Experiment 4). Collectively, our findings suggest the confusion stems from the upper part of a face. We suspect that it may be resulted from overlapping of the facial features on upper half faces between anger and disgust.

## Keywords

facial expression, disgust, anger, confusion

## Acknowledgments

This study was supported by MOST105-2410-H-039-002; MOST103-2410-H-039-001-MY2.

## Color and Surface

## Temporal Structure of Blue Colour Processing—A MEG Multifocal Study

**David Crewther and Laila Hugrass**

Swinburne University of Technology, Victoria, Australia Email: dcrewther@swin.edu.au

## 

Nonlinear VEP has been used to separate magno and parvocellular contributions to the evoked potential, on the basis of contrast gain, amplitude saturation and latency. Here, we studied MEG cortical multifocal responses to diffuse blue stimulation as a function of colour saturation. Nine university age students with normal colour vision participated. An m = 14 pseudorandom stimulus sequence was applied to eight segments each fluctuating between blue colour (95%, 75%, 50%, 25%, and 0% saturation) and an achromatic stimulus (grey) of higher luminance. An Elekta TRIUX MEG system recorded magnetic-evoked fields. First- and second-order Wiener kernels were analysed using Brainstorm with data co-registered on 0.75 mm isovoxel MRI images. While sensor cluster first-order kernels showed some increase in amplitude with saturation, the greatest chromatic sensitivity was demonstrated by the N60P90 peak of the first slice of the second-order response, whose amplitudes showed a roughly linear function of saturation across each of the four central stimulus quadrants. Initial minimum norm source estimates show the main contribution to come from striate cortex. While such stimuli would be expected to reflect koniocellular function, further questions are posed by the short latencies recorded—similar to those attributed to magnocellular function.

## Keywords

MEG, blue saturation, nonlinear kernels, cortical evoked fields, multifocal stimulation

## Acknowledgments

This study was funded by Australian Research Council.

## Glossiness Perception not Depending on Specular Highlights—Impacts of Luminance Edges

**Hiroaki Kiyokawa, Tomonori Tashiro, Yuki Kawashima, Yasuki Yamauchi and Takehiro Nagai**

Department of Informatics, Yamagata University, Yamagata, Japan Email: tmk61083@st.yamagata-u.ac.jp

## 

Human observers perceive glossiness on object surfaces from rather low luminance regions without specular highlights (Kim et al., 2012). However, it has been unclear what image information was a cue for perceived glossiness based on low luminance regions. To examine this issue, we performed glossiness rating experiments using a number of computer-graphics images of objects with different shapes, reflectance properties, and light fields. There were two conditions about stimulus luminances: The normal condition in which computer-graphics images were directly used and the no-highlight condition in which luminances of specular highlights were clipped. In the results, glossiness rating scores were higher in the no-highlight condition than the normal condition in some objects, demonstrating that low-luminance regions more strongly contribute to perceived glossiness than specular highlights in certain conditions. Furthermore, a Laplacian filter analysis showed that, on the objects on which the rating scores were higher in no-highlight condition, amounts of luminance edges on object surfaces were strongly correlated with the rating scores, while they were only weakly correlated on the other objects. These results suggest that luminance edge components on object surfaces are an effective cue for perceived glossiness even when high luminance regions do not exist.

## Keywords

material perception, glossiness, luminance edge

## Visual Perception of Pigmentation on Facial Skin-Color Distribution

**Yu Fang, Yoko Mizokami and Hirohisa Yaguchi**

Graduate School of Engineering, Chiba University, Chiba, Japan Email: yfang@chiba-u.jp

## 

Studies have shown that facial skin pigmentation is an important cue on human physical attraction judgment. However, little is known about how skin color information influences the perception of facial skin pigmented spot. We compared the visual perception of the facial skin pigmentation with various color densities between two backgrounds, which are only different in color distribution. In a two-alternative forced choice paradigm, subjects were asked to judge the existence of a skin pigmented spot on a background image. The background image was a cheek picture or a random pixel image created from the same cheek picture. Stimuli were displayed for a period of 300 ms and followed by a mask with a period of 200 ms. Performance was tested with different pigmentation color densities which divided the distance between the facial skin and the pigmentation into equal 20 scales in the CIELAB Color Space. The result indicated that the performance of pigmented spot judgment increased with the increasing of the color density of the skin pigmentation. This result was only found when the pigmented spot appeared on the background of the cheek skin image, suggesting that a facial skin color distribution plays an important role in face perception.

## Keywords

pigmentation perception, facial skin color, face perception, color density

## Acknowledgments

This study was supported by JSPS KAKENHI 16H01663.

## Poster Sessions Face Perception

## The Effect of Self-Construal on Facial Expression Detection

**Po-Shiuan Tsai and Pi-Chun Huang**

Department of Psychology, National Cheng Kung University, Tainan, Taiwan Email: pichun_huang@mail.ncku.edu.tw

## 

Previous studies have shown that the detection and discrimination abilities of a target are modulated when it surrounded by other stimuli. A similar effect was also found during the judgment of the emotional state and showed that people with different self-construal (interdependent or independent) perceive different emotional intensities of the target face when it is surrounded by a crowd of emotional people. In this study, we investigated whether self-construal plays a role in facial expression detection. The observers were divided into two groups via the Independent and Interdependent Self-Scale. They were then asked to categorize the target faces as happy or angry while the target facial expressions morphed to five levels varying between angry and happy faces. The targets were surrounded by four different face conditions (happy, angry, neutral, inverted neutral), and a baseline condition with no surrounding face was presented. The results showed that, when it comes to the same facial expression, independent people tend to detect the face as less happy than interdependent people. The detection of a target facial expression was not affected by the face surrounding it. More researches are needed to clarify the effect of the difference in perceptual level and decision bias.

## Keywords

facial expression, emotion discrimination, emotion categorization, surrounding emotion face

## Acknowledgments

This work was supported by MOST 104-2628-H-006-001-MY3 to PCH.

## Development and Validity Study of the Korean Version of Cambridge Mindreading Face Battery (Yonsei-CAM)

**Donghyun Oh and Eunsun Chung^1^**

Yonsei University, Seoul, South Korea Email: eun930320@gmail.com

## 

Accurately identifying other’s emotional states is crucial for fluent social interaction. The Cambridge Mindreading (CAM) Face Battery is widely used to assess one’s emotional recognition ability with 24 sets of video-taped subtle emotional expressions. However, the video stimulus is limited to Caucasian population and includes emotional expressions uncommon in Asian culture. Thus, the purpose of this study is to develop and validate a Korean version of CAM (Yonsei-CAM). This study incorporated 18 commonly used emotional expressions in Korea which were chosen on the base of 80% agreement from 11 reviewers. The selected expressions were then recorded with 56 female and male Korean actors aged between 20 to 40. After all the clips had been received, two independent observers compliant to the observer reliability selected 5 s from each clip that best represent the corresponding emotional expression. The final clips will be selected upon three criteria after conducting an assessment with 120 college students: the accuracy rate of tasks, the reported degree and the smoothness of the emotions. Y-CAM can contribute in diverse fields of academia as the face stimuli can be utilized in studies with Asian population.

## Keywords

The Cambridge Mindreading Face Battery, video-taped face stimuli, emotional recognition assessment, emotional expressions

## Acknowledgments

This study was supported by Yonsei University and Brain Korea 21.

## The Influence of Head Orientation on Perceived Gaze Direction and Eye Region Information

**Yumiko Otsuka^1^ and Colin Clifford^2^**

^1^Faculty of Law and Letters, Ehime University, Matsuyama, Japan

^2^School of Psychology, UNSW Australia, NSW, Australia Email: otsuka.yumiko.wf@ehime-u.ac.jp

## 

We examined the influence of head orientation on perceived gaze direction by using a categorization task and adjustment of an on-screen pointer. In both tasks, we found that the repulsive influence of head orientation on perceived gaze direction was greater when only the model’s farther eye was visible compared to when only the nearer eye was visible. When both eyes were visible, the two tasks revealed a different pattern of results suggesting a flexible use of information from two eyes depending on the task. We also analyzed the relative position of iris within the eye-opening in the stimulus. The analysis revealed a greater repulsive influence of head orientation for the model’s farther eye compared to nearer eye, which is consistent with the perceptual performance. The image analysis also revealed that the repulsive influence of head orientation on image eye region information was much greater than previously inferred based on gaze judgment performance with eye-only images (Otsuka et al., 2014, 2015). The current results suggest that observers in our previous studies used residual head orientation cues in the shape and/or relative size of the two eyes to counteract the repulsive influence of head orientation, causing an underestimation of the repulsive effect.

## Keywords

eye gaze, head orientation, repulsive effect, attractive effect, dual route model

## Acknowledgments

This study was supported by an Australian Research Council Discovery Grant (DP160102239 to CC) and JSPS KAKENHI (15H06456 to YO).

## Taiwanese Face Database 2.0

**Claire Y.-J. Li^1^, Vicky, Y.-H. Chen^1^ and Gary C.-W. Shyi^2^**

^1^Department of Psychology, National Chung Cheng University, Taiwan

^2^Center for Research in Cognitive Sciences, National Chung Cheng University, Taiwan Email: cwshyi@gmail.com

## 

Building a culturally calibrated face database with variations in identity, expression, pose, and lighting, can meet the demand of a multitude of face research. The present study represents an extension of our previous effort in this regard (Shyi, Huang, & Yeh, 2013), where subsequent studies have exposed its limitations in fulfilling the requirements placed by experimental manipulations. A total of 6,600+ face photos, varying in terms of expression, pose, lighting, and gaze direction were taken from a group of 36 female and male models. About half of them (3,300+) were rated by another group of 120 participants to ensure that each face image was rated by at least 10 different participants. We then categorize each image into one of the seven basic emotions in terms of intensity and polarity. In order to compare the current and the previous databases, we conducted analyses to examine how pose, intensity and direction of lighting may affect the ratings and entropy values associated with each image. The overall results from these analyses show patterns remarkably similar to the previous database. Taken together, we consider the current database and the previous database are empirically equivalent, and can be linked into a larger and more useful source for face research.

## Keywords

facial expressions, face database, pose, illumination, entropy

## The Effect of Context in Facial Emotion Recognition in Children With Autism Spectrum Disorder

**Jiyoung Noh and Kyongmee Chung**

Yonsei University, Seoul, South Korea Email: kmchung@yonsei.ac.kr

## 

Autism spectrum disorders (ASD) are characterized by deficits in social interaction and communication, as well as restricted behaviors and interests. A body of literature shows that ASDs have impairments in recognition of facial emotion which may contribute to social impairment. Most studies use faces presented alone without any context as stimuli, suggesting strong and automatic influence of context on face recognition. Consequently, prior studies for ASDs may be limited in the sense that they lack potentially important contextual cues and may not fully capture the difficulties experienced in everyday life. This study investigated quantitative change in emotional intensity ratings with the addition of contextual information in children with ASDs. Participants in the current study were 19 children with ASD. All participants were asked to assess the emotional intensity of a single emotion (happy/anger) from images presented under two conditions (context-free and context embedded). The results showed there is no significant difference in the addition of contextual cues. To suggests that ASDs have impairment in using contextual cues to moderate their assessment of emotional intensity, additional experiments for control group (typically developing children) will be conducted.

## Keywords

autism spectrum disorders, facial emotion recognition, effect of context

## Acknowledgments

This study was supported by Yonsei University and Brain Korea 21.

## Not all Races are Preferred Equally: Exploring the Development of Race-Based Social Preferences in Taiwanese Children

**Pei-Chun Hsu^1^, En-Yun Hsiung^2^ and Sarina Hui-Lin Chien^3,4^**

^1^Graduate Institute of Neural and Cognitive Sciences, China Medical University, Taichung, Taiwan

^2^Department of Pharmacy, Graduate Institute of Biomedical Sciences, China Medical University, Taichung, Taiwan

^3^Graduate Institute of Neural and Cognitive Sciences, China Medical University, Taichung, Taiwan

^4^Graduate Institute of Biomedical Sciences, China Medical University, Taichung, Taiwan Email: sarinachien@mail.cmu.edu.tw

## 

In developing the fifth core knowledge about social partners, race is an important factor biasing children to form social affiliations. The present study explored the development of the race-based social preferences in 3- to 8-year-old Taiwanese children. In Experiment 1, 21 three to four year olds viewed three simultaneously presented video clips modeled by a Taiwanese (own race, high social status, in-group), a Southeast Asian (near race, low social status, out-group) and a Caucasian (other race, high social status, out-group) young female smiling at them. Children were instructed to give a toy to their most preferred and second preferred individuals, and children preferred the Taiwanese actress the most (50%). In Experiment 2, 22 five to six year olds viewed the same videos and were instructed to choose their most preferred and second preferred persons as friends. They preferred the Taiwanese actress the most (69%). In Experiment 3, 21 seven to eight year olds performed the same task as in Experiment 2 and they preferred the Caucasian actress (57%) the most and the Southeast Asian actress the least. In sum, our findings suggest that a rudimentary race-based social preference (or prejudice) seems to emerge early in childhood. These results provide a cross-cultural exploration about the development of race-based social judgments.

## Keywords

race, core knowledge, social categories, social preference, cognitive development

## Acknowledgments

This study was funded to Dr. Chien (MOST 103-2410-H-039-002-MY3, MOST 105-2420-H-039-001-MY3, MOST 105-2632-B-039-003).

## The Importance of the First Fixation for Recognising Own- and Other-Race Faces: An Eye-Tracking Study

**Hoo Keat Wong^1^, Ian Stephen^2^ and David Keeble^1^**

^1^University of Nottingham Malaysia Campus, Malaysia

^2^Macquarie University, Australia Email: khyx4whe@nottingham.edu.my

## 

Hills and Lewis (2006) reported that the own-race bias (ORB) in face recognition was reduced by cueing Caucasian participants to the lower region of African faces. However, recent empirical studies have failed to replicate this finding. This cross-cultural study investigated whether shifting initial attention to different facial regions affects own- and other-race recognition performance. In a classic yes–no recognition task, Malaysian-Chinese, Australian-Caucasian, and African participants were presented with a series of Chinese, Caucasian, and African faces that had facial regions (i.e., eyes, nose, mouth) cued with fixation crosses. Split analyses by race groups revealed that there was a pronounced ORB only in Caucasian participants. Most importantly, cueing Chinese participants to the nose region and Caucasian participants to the eyes and nose regions enhanced their subsequent recognition performance compared to cueing to the mouth region. These results suggest that immediate fixations directed to the upper facial regions, especially the nose region, somewhat encourage holistic face processing and/or decrease the engagement of featural processing and may enhance the encoding of individuating diagnostic features for recognition.

## Keywords

face recognition, own-race bias, eye movements

## Parts-Based Facial Attractiveness Judgment Is Modulated by Attention to Detail

**Chihiro Saegusa^1^ and Katsumi Watanabe^2,3^**

^1^Kao Corporation, Japan

^2^Waseda University, Japan

^3^The University of Tokyo, Japan Email: saegusa.chihiro@kao.co.jp

## 

Contribution of each facial part to attractiveness judgment of the whole face depends on exposure duration (Saegusa & Watanabe, 2016). In the current research, we examined how evaluator’s attention to detail would interact with the dynamic integration process of facial parts onto facial attractiveness judgment. Ninety-six participants evaluated the attractiveness of 98 whole facial photographs (58 female and 40 male models) and also isolated facial parts. The autism-spectrum quotient (AQ) was obtained from the participants. Multiple regression models explaining whole facial attractiveness with facial parts attractiveness were built for each participant. Then, we examined whether the sub-scores of AQ would show significant correlations with the contributions of facial parts attractiveness to whole face attractiveness. Our results demonstrated that, for participants who showed lower scores in the AQ sub-score of “focus to the detail,” the contributions of the eyes were lower. The finding indicates that the evaluator’s attention to detail interacts with the dynamic integration process of facial parts onto facial attractiveness judgment.

## Keywords

facial attractiveness perception, perceptual organization, AQ

## Representation of Facial Identity and Expression in Persons With Autism Spectrum Disorder: Identity- and Expression—Contingent Aftereffect

**Hyangkyeong Oh and Kyong Mee Chung**

Yonsei University, South Korea Email: kmchung@yonsei.ac.kr

## 

Recent studies revealed that persons with ASD showed reduced aftereffects in both facial identity and expression in adaptation paradigm. However, no previous study has examined whether these two aftereffects are independent or interdependent. The purpose of this study is to investigate ‘expression-contingent’ identity aftereffect (Experiment 1) and ‘identity-contingent’ expression aftereffect (Experiment 2) in ASD using an adaptation paradigm. The two experiments were done with eight children with ASD. In Experiment 1, two male faces with expression were used as adaptors, and 13 identity-morphed images are used as probes. First, participants were shown the adaptor for 5,000 ms. Then, morphed images with either congruent or incongruent expression with the adaptor were presented for 500 ms. Participants were asked to discriminate the ‘identity’ of the probe. In Experiment 2, the procedure was identical to that of Experiment 1, except that ‘expression’ morphed images of either the congruent or incongruent identity with the adaptor were presented as probes, and participants were asked to discriminate the ‘expression’. Paired *t*-test was conducted to compare the accuracy between congruent and incongruent condition. There was no significant congruency effect in both Experiments 1 and 2. These findings suggest that independent, not integrated face coding mechanisms of identity and expression in persons with ASD.

## Keywords

autism spectrum disorder, face perception, norm-based coding model, face adaptation paradigm, aftereffect

## Acknowledgments

This study was funded by Yonsei University and Brain Korea 21.

## Yawning Face Detection Sensitivity and Yawning Contagion

**Hiu-ming Chan^1^ and Chia-huei Tseng^2^**

^1^University of Cambridge, UK

^2^Tohoku University, Japan Email: CH_Tseng@alumni.uci.edu

## 

Contagious yawning—the urge to yawn when thinking about, listening to, or viewing yawning—is a well-documented phenomenon in humans and animals. While clinical studies have suggested the association between empathy and contagious yawning frequency, whether there is a perceptual component is not studied comprehensively yet. In this study, we examined influences from perceptual factors (i.e., individuals’ eye gaze pattern and perceptual detection sensitivity to yawning, happy, and angry faces) on 41 non-clinical adults. We induced contagious yawning with a 5-min video and 20 yawning photo stimuli, and we measured participants’ eye gaze patterns, perceptual detection thresholds to human yawning and facial emotions (happy or angry) and autistic traits (with the Autism-Spectrum Quotient Questionnaire). We found two factors associated with yawning contagion: (a) those more sensitive to detect yawning, but not other emotional expressions, displayed more contagious yawning than those less sensitive to yawning expressions; (b) female participants exhibited significantly more contagious yawning than male participants. We did not find an association between autistic trait and contagious yawning after controlling for gender and yawning sensitivity. Our study offers a working hypothesis for future studies, in that perceptual encoding of yawning interacts with susceptibility to contagious yawning.

## Keywords

face perception, perceptual detection sensitivity, eye gaze pattern, contagious yawning, individual difference

## Motion Perception

## Vertical Size Disparity Processing on Elements Moving in Opposite Directions

**Yuta Miyanishi and Hirohiko Kaneko**

Tokyo Institute of Technology, Japan Email: yuta.miyanishi@k.ip.titech.ac.jp

## 

It has been reported that vertical disparities distributed in a certain spatial and temporal region are pooled to produce stereoscopic perception of the curvature, inclination, and slant of a surface. However, Duke & Howard (2005, 2012) reported that vertical disparities in the same region are not pooled when they are separated in depth defined by horizontal disparity. In other words, vertical disparity is processed in each depth region specified by the horizontal disparity. We investigated the effect of a depth separation defined by relative motion, instead of horizontal disparity, on the vertical disparity pooling. The stimulus consisted of two sets of dots, and they were uniformly intermingled. They had opposite signs of vertical size disparity and opposite directions of motion. Their horizontal disparities relative to the screen were kept at zero. Observers responded perceived slants of one or two surfaces of the stimulus. As a result, when the relative speed of the two set of dots was high, two separated slants were perceived. When the relative speed was adequately low, single surface was perceived. The transition of slant perception seemed to be dependent on the relative speed itself, regardless of the magnitude of depth separation produced by the relative motion.

## Keywords

stereopsis, vertical disparity, slant perception, depth from motion

## Acknowledgments

This study was supported by JSPS KAKENHI (Grant Number 15H02725).

## Measurement of Visual Attraction Strength to Object Motion by Gaze-State and Method of Paired Comparison

**Sae Nakanishi^1^ and Keizo Shinomori^2^**

^1^Graduate School of Engineering, Kochi University of Technology, Japan

^2^School of Information, Research Institute, Kochi University of Technology, Japan Email: 205082e@gs.kochi-tech.ac.jp

## 

In this study, we measured how motion of the object is related to the visual attraction. We focused effects of the characteristics of the object in motion to the visual attraction in different conditions in changing one of the parameters; number of elements in the object, element size, motion direction and a speed of the motion. We presented a pair of two different stimuli side by side and measured subject's eye movement in the observation to the stimuli. From the eye movement, we obtained the looking time to the left- and right-side stimuli. We simultaneously performed paired comparison between stimuli in the same presentation way and estimated the attraction intensity by z-scores of each stimulus calculated from selection rates. From these values, we firstly found that the highest attraction is obtained by the maximum number of elements (9 in 1 to 9), largest size (2.2 deg in 0.44 to 2.2 deg), upper-left direction in 8 directions, and fastest speed (7.3 deg/s in 3.1 to 7.3 deg/s). We are now measuring the attraction in the new condition in which the size and number of elements are in trade-off relationship keeping the same area size.

## Keywords

attractiveness, attention, gaze-state (eye movement), paired-comparison

## Acknowledgments

This study was supported by Priority Research Laboratory of Kochi University of Technology.

## Surface Properties and the Perception Self-Motion

**Andrew-Charbel Salloum^1^, Stephen Palmisano^2^ and Juno Kim^1^**

^1^University of New South Wales, Australia

^2^University of Wollongong, Australia Email: juno.kim@unsw.edu.au

## 

Optic flow is generated whenever observers move relative to stationary objects in their environment. However, this optic flow is inherently ambiguous because the motion of environmental objects relative to a stationary observer can often produce similar patterns of visual motion. Many previous studies have shown that illusory experiences of self-motion (vection) can be generated when stationary observers view optic flow presented on a digital display. Most of these studies only considered random-dot displays where elements mimic the viewpoint-independent surface optics of diffuse reflectance. However, real-world surfaces also generate specular reflections that are viewpoint dependent. Very different visual motion velocities can be attributed to these specular and diffuse optic flows. In this study, we identified visual motion constraints in diffuse and specular optic flows that could be used to disambiguate object-motion from self-motion. We used the Oculus Rift to test whether vection varies differentially across conditions with and without specular reflections. The results support the view that the brain processes information about surface properties before visual motion is attributed to object-motion or self-motion.

## Keywords

vection, materials, surface properties, reflectance, 3D shape

## Acknowledgments

Australian Research Council (ARC) grant was awarded to Juno Kim.

## Randomly Updating Images on Coherence Global Motion

**Xirui Yang^1^, Chien-Chung Chen^2^ and Hiroshi Ashida^1^**

^1^Graduate School of Letters, Kyoto University, Japan

^2^Department of Psychology, National Taiwan University, Taiwan Email: yangxirui93@yahoo.co.jp

## 

Glass (1969) patterns are formed by pairs of random dots (dipoles) which could carry a powerful percept of global structure. The perceived motion direction of a dynamic Glass pattern is influenced by orientation of the dipoles (Krekelverg et al., 2003). Here, we investigated whether just updating random dots would form a specific global motion. Five types of moving stimuli, including linear, random, concentric, radial and updating, were shown through either a circle or square aperture. The task of the participants was to judge the perceive strength of concentric, linear, radial movement a seven-point Likert scale for each stimulus. In all viewing conditions, participants indeed showed a response bias toward concentric motion. The bias was particular strong in the circle aperture. Such effect of aperture shape was the most pronounced for the linear motion stimuli. These results show that just updating the position of dots in a random dot image is sufficient to generate a percept of concentric global motion. The effect of dipole orientation in the previous dynamic Glass pattern studies may be a secondary effect.

## Keywords

motion perception, random dot, global motion

## Objects

## The Effect of Training Paradigm in Greeble Expertise Acquisition: A Multi-Voxel Pattern Analysis Approach

**Han-Shin Jo^1^, Kuo Liu^1^, Chiu-Yueh Chen^1^ and Chun-Chia Kung^2^**

^1^National Cheng Kung University (NCKU), Taiwan

^2^Mind Research and Imaging (MRI) Center, National Cheng Kung University (NCKU), Taiwan Email: gockgange@gmail.com

## 

The fusiform face area (FFA) has often been speculated as a brain region that is specialized for face perception and recognition. While it is generally believed that the FFA responds selectively more to facial stimuli than other objects, the expertise hypothesis proposes that the FFA may participate in the processing of any object class that is trained to be processed at the subordinate or individual level. Next poster gives the FFA evidence of how two different training regimes (Gauthier et al., 1997, Vision Res., 37 (12), pp. 1673–1682; vs. Gauthier et al., 1998, ibid., 38/15, pp. 2401–2028) yield different FFA responses. The multi-voxel pattern analysis is used to distinguish the patterns of FFA activity between Greebles and other stimuli (Faces and Objects), and we demonstrate that activity patterns of localized FFA perform better at distinction of “Faces vs. Greebles” in before-than after-training does, and in “Greebles vs. Objects” better in after- than before-training does. In both cases, the Gautheir 97 paradigm has shown more prominent distinction results than the Gauthier 98 paradigm. In addition, searchlight information mapping is employed to identify other brain regions that can provide information concerning the neural representation of distinct object classes.

## Keywords

perceptual expertise, greeble training, functional MRI, multi-voxel pattern analysis, searchlight information mapping

## Perceptual Expertise Predicts Both Gray Matter Thickness and Density in the Human Fusiform Gyrus: A Cross-Country MRI Study on Bird Experts

**Yi Lin, Chun-Chia Kung and Nian-Ting Yang**

NCKU, Taiwan Email: a10230709@gmail.com

## 

In one recent study, cortical thickness (CT) of car experts' Fusiform Face Area (aka FFA) were correlated with their face and object (car) performance (McGugin, et al. (2016) JoCN 28, pp. 282–294). To both extend this finding from car experts to experts of other domain, and also expand the CT and cortical volume, in Study 1, we reanalyzed our previously acquired birder MRI data (*N* = 27 Caucasians), with both audiovisual and visual dprimes as their expertise measure. The results showed that significant correlations were found in both voxel density and CT between both audio and visual d', especially in bilateral fusiform gyrus, dorsal anterior and posterior cingulate gyrus, etc. After partialling out the age confound, controlling for the high correlation between expertise and age, these results still hold. In Study 2, we corroborated the similar results MRI data (*N* = 20, with only visual dprimes) acquired in Taiwan. Lastly, the joint analyses combining both America and Taiwan data (*N* = 47) showed that the left fusiform gyrus remained highly correlated, further strengthening the role of FG in expertise. Despite slight disparities, the brain regions are overall highly similar across VBM- and CT-expertise correlations not only extending the previous CT-expertise in car to bird experts but also expanding the CT-expertise to VBM-expertise correlations, deepening the interconnection between experience and brain structure.

## Keywords

fusiform gyrus, cross-country, experts, gray matter thickness, gray matter density

## Spatial Vision

## Recognition Thresholds for One-Letter Versus Two-Letter Stimuli in the Periphery

**Pei-Shan Sung, Wei-Ming Huang, Chun-I Yeh and Lothar Spillman**

Department of Psychology, National Taiwan University, Taiwan Email: sungpeishan@gmail.com

## 

Is visual acuity for a two-letter stimulus the same or higher than for a one-letter stimulus? Previous experiments in central vision have shown that two characters if presented next to each other are more difficult to recognize than each character by itself. This is called crowding. We tested this finding with one-letter and two-letter stimuli presented in the periphery. Ten black-on-white letters ranging from 0.4 to 5.1 deg in height and presented at 10, 30, and 50 deg in the temporal visual field were used. Observation was with the right eye. The method of ascending limits was used. At 30 deg and 50 deg eccentricity, the threshold for recognition of two letters was markedly higher than for one letter, confirming the above hypothesis. At 10 deg, results were obscured by a ceiling effect. The difference between one and two letters increased with retinal eccentricity. We attribute this difference to increased spatial interference. Results are consistent with Bouma’s (1972) critical distance rule, showing that two characters need to be spaced by a certain amount to prevent crowding. We kept the distance between pairs of letters constant, but increased their size relative to a single letter in order to make them recognizable.

## Keywords

crowding, visual eccentricity, visual acuity

## Acknowledgments

This study was supported by 104C9364-2.

## Tilt Illusion From Interocular Grouping: Can Conscious Grating Induce the Tilt Illusion?

**Young Hun Sun and Woo Hyun Jung**

Chungbuk National University, South Korea Email: com4man@gmail.com

## 

The interocular grouping can occur when different parts of a visual stimulus are presented to each eye during binocular rivalry. The main purpose of this study is to test whether the dominant visual object from interocular grouping can induce the tilt illusion. The stimuli were three overlapped circles which had different sizes but same center. Each circle had square wave gratings which had different orientations. Two surrounding circles designed to be perceived as single grating had a same orientation when the interocular grouping occurred. The integrated grating could induce repulsive or attractive effect of the tilt illusion. In the Experiment 1a, the participants subjectively responded for the dominant duration and alternations of the interocular grouping. The orientations and sizes of two surround gratings varied. The interocular grouping was induced irrelevant of orientations and sizes. In the Experiment 1b, the participants chose which direction the orientation of the grating in the center was tilted to. The result showed that the integrated grating induced the repulsive effect of the tilt illusion, but not attractive effect. These findings suggest that the attractive and repulsive effects of the tilt illusions during interocular grouping can be processed separately between conscious and visual processing levels.

## Keywords

interocular grouping, tilt illusion, binocular rivalry, consciousness

## The Competition Between Gestalt Similarity and Closure Laws

**Ya-Ching Su and Chien-Chung Chen**

Department of Psychology, National Taiwan University, Taiwan Email: c3chen@ntu.edu.tw

## 

Similarity and closure are two important cues for perceptual grouping. We investigated how these two cues interact with each other when they coexisted in an image. We used tripole Glass patterns which consisted of randomly distributed sets of three elements, including one seed and two context elements. Each local element had three bars arranged in U-shape. One context element was the same as the seed (similarity) while the other one was a 180°-rotated version of the seed (closure). The tripoles were arranged in a way that linking the seed with the one context would produce a clockwise (CW) spiral percept while the other one, counter-clockwise (CCW) spiral. The contrast of the context elements ranged from −20 to 0 dB, while the seed contrast was fixed at −10 dB. Observers’ task was to indicate whether the test image was a CW or CCW spiral. We found that the observers were more likely (10%) to perceive a global pattern based on grouping by similarity than by closure. Such orientation similarity effect is consistent with a token matching theory of Glass pattern perception.

## Keywords

Gestalt laws, perceptual grouping, tripole Glass patterns

## Acknowledgments

This study was funded by Ministry of Science and Technology, R.O.C. (105-2420-H-002-006-MY3).

## The Spatial Frequency Effect on Blackshot Mechanisms for Texture Perception

**Chien-Chung Da Li, and Chen**

National Taiwan University, Taiwan Email: c3chen@ntu.edu.tw

## 

It is suggested that textures discrimination is mediated by a mechanism that is very sensitive to the lowest luminance in the texture. We investigated the role of spatial frequency on the function of such blackshot mechanisms in texture discrimination. We used two types of spatial frequency filtered random patterns. The first had 1/f-like spectrum with slope ranging from 0 to 1. The second was the bandpassed noise with 1 octave bandwidth and a peak frequency ranged from 2 to 32 cyc/deg. The white noise was constructed by a linear combination of the second-order Legendre polynomial and one of the other order polynomials. The observers were instructed to discriminate between textures based on the difference in the second-order polynomials. We estimated the contrast-sensitivity profile by a maximum likelihood method. The contrast sensitivity profiles for all patterns peaked at the lowest contrast, consistent with the blackshot mechanisms. The blackshot sensitivity increased and then decreased with spectral slope, with peak at slope of 1. For bandpass patterns, the blackshot sensitivity decreased with the central frequency. Thus, the blackshot mechanisms are low passed and optimal for textures with a spectral slope similar to that of natural scenes.

## Keywords

texture perception, blackshot mechanism, spatial frequency, 1/f spectral slope

## Oblique Effects Measured Using the Method of Adjustment in Young Adults and Children

**Hiroko Sumida and Goro Maehara**

Kanagawa University, Japan Email: sumidahiroko.ku@gmail.com

## 

Measurements of threshold and reaction time are time consuming in practice and difficult for children with problems paying attention and difficulty controlling behavior. We measured the oblique effects using a rotatable disk for adjustment of line orientation. The observers were 15 young adults and 18 normal children. They matched the orientation of a line segment on the rotatable disk with that of a standard line segment. The orientation of the standard stimuli ranged from 0 to 157.5 deg (8 levels). Young adults and children, respectively, conducted 8 or 4 trials for each standard orientation, 64 or 32 trials in total. The mean errors were smaller for 0 and 90 deg than for 67.5 and 112.5 deg. SDs of the mean errors were also smaller for 0 and 90 deg. These results indicated that, compared with oblique line segments, observers’ judgments were generally precise concerning horizontal and vertical line segments. The mean errors and SDs were larger for children than for young adults. We consistently observed the oblique effects using the rotatable disk stimulus. It takes only about 10 min for children to complete 32 trials. Our apparatus could be useful for testing inattentive children.

## Keywords

oblique effect, development, adjustment

## Examining the Relative Strength of Proximity and Similarity Laws Using Tripole Glass Patterns

**Lee Lin and Chien-Chung Chen**

Department of Psychology, National Taiwan University, Taipei, Taiwan Email: c3chen@ntu.edu.tw

## 

Similarity and proximity are important grouping principles. A quantitative estimation of the relative strength of these principles is sparse in the literature. To address this issue, we manipulated these principles within a tripole Glass Pattern (tGPs), which consisted of random-distributed groups of dots. Each group contained one anchor dot and two context dots. An observer would perceive a clockwise (CW) spiral by grouping the anchor to one context dots and counterclockwise (CCW) spiral, the other. The luminance contrast of the context dots varied between −30 and 0 dB. The anchor-context dot distance varied between 2.5 and 20 min. Participants were to report whether they perceived CW or CCW in the tGP in each trial. In all conditions, the probability of seeing a CW spiral first increased then decreased with the increase of CW dot contrast. The peak of such inverted-U shape functions shifted rightward as CCW dot contrast increased. Manipulation of anchor-context distance showed a trade-off between similarity and proximity that peaked when the anchor-context distance was 5 min, which required about 6 dB contrast difference to cancel the proximity advantage. These results could be explained by a “divisive inhibition model” rather than the traditional energy or token-matching theory.

## Keywords

Glass pattern, proximity, perceptual grouping, luminance contrast, contrast gain control

## Acknowledgments

This study was funded by MOST (Taiwan) 103-2410-H-002-076-MY3.

## Using the Oculus Rift to Understand the Perception of Shape From Material Flow

**Masakazu Ohara^1^, Juno Kim^2^ and Kowa Koida^3^**

^1^Department of Computer Science and Engineering, Toyohashi University of Technology, Japan

^2^School of Optometry and Vision Science, University of New South Wales, Australia

^3^Electronics-Inspired Interdisciplinary Research Institute, Toyohashi University of Technology, Japan Email: juno.kim@unsw.edu.au

## 

How do we effortlessly untangle complex mashes of image structure to visually infer the shape of 3D objects? We explored whether visual motion cues from different surface optics improve global shape perception. We created virtual objects varying in surface optics (matte, specular, refractive), relief (smooth, bumpy), and convexity along the viewing axis. Observers viewed stereoscopic simulations on the oculus rift of these objects oscillating horizontally for 5 s. The seated observers were asked to report whether the shape of the 3D object appeared elongated (like an Aussie football) or flat (like a pancake). We found that global surface convexity was perceptually overestimated for “flat” specular surfaces compared with diffusely-shaded surfaces (similar to Mooney and Anderson, 2014). However, we further found that “flat” refractive objects generated percepts of shape that were closer to veridical. We also found that elongated surfaces were underestimated in global convexity overall, but oscillating smooth elongated specular surfaces were perceived closer to veridical. The amplitude of oscillation improved shape discrimination for both smooth and bumpy surfaces. These results reveal a form of seesaw effect, whereby errors in perceived shape of objects differentially depends on motion cues from multiple forms of surface optics as curvature increases.

## Keywords

oculus rift, material perception, visual perception

## Acknowledgments

This study was supported by Leading Graduate School Program R03 of MEXT to MO, ARC Future Fellowship to JK, KAKENHI (15H05917) to KK.

## Binocular Vision and Space Perception

## Interactive Processing of 2D and 3D Cues in Stereopsis Vision

**Jy-Chyi Yuan**

Department of Psychology, Fu-Jen Catholic University, Taiwan Email: yuanjc2000@gmail.com

## 

How binocular matching happens on stereoscopic vision with and without 2D-boundary-cues are our basic interesting. In this study we compare two kinds of presentation ways (goggle-glasses & two-mirror-screens) and three kinds of images which have different richness of 2D-boundary-cues (Random-Dots-Stereogram, dot-pattern, real scenes). Two images of left-right eyes superimposed in goggle-glasses presentation, so produces new binocular-disparity boundary cues or ghost. Two-mirror-screen presentation doesn't have superimposed binocular-disparity generated 2D-boundary-cues, so RDS fusion supposed to be more difficult in this presentation way. Result shows reaction time of 3D perception of RDS will be shorter by goggle-glasses presentation, but not by two-mirror-screen presentation. Dot-pattern and real scenes don't have this difference. We think it's because of RDS need to do local matching of zero-crossing first then perceived 3D. With contour pictures (dot-pattern and real scenes) might direct match 2D-boundary-cues globally. So we conclude that stereoscopic fusions are dynamic interaction of global and local matching.

## Keywords

stereopsis, 2D global matching, 3D local matching

## Walking on Sunshine—Anisotropy of Egocentric Distances Perceived by Walking

**Oliver Toskovic**

Laboratory for Experimental Psychology, Faculty of Philosophy, University of Belgrade, Serbia Email: otoskovi@gmail.com

## 

We hypothesized that vertical distances are perceived as larger than horizontal because motion on vertical direction acquires more effort because of the gravity. Based on that we asked what would happen if observer would estimate distances by walking toward stimuli. Two experiments were done in a gym, with 31 (14 + 17) participants matching distances of two stimuli on horizontal and vertical direction. Participants would look at the stimuli (on 1 m, 1.5 m or 2 m), then put a blindfold on eyes, count backwards by three, and walk or climb on leathers for the same distance. In first experiment we measured anisotropy effects and participants walked/climbed on direction opposite to stimuli, while in the second, we controlled for the effects of fear from climbing and they walked/climbed on the same direction. If the participants walked/climbed on opposite direction, there were significant effects of direction and distance and their interaction. Participants were matching shorter vertical distances to longer horizontal ones, which means that vertical distances were perceived as longer. If they walked on same direction, only effect of stimuli distance was significant, but no effects of direction. These findings are consistent with the hypothesis on gravity integration into perception action schemes.

## Keywords

anisotropy, perceived distance, viewing direction, perception action schemes, proprioception

## Acknowledgments

This work is supported by Ministry of education and science Republic of Serbia, project number 179033.

## Color and Lightness

## Evaluation of Shape-Level Depth Adaptation by Using Disparity-Specified Structures and Noise-Shape Stimuli

**Shufang He^1^ and Hiroaki Shigemasu^2^**

^1^Graduate school of Engineering, Kochi University of Technology, Japan

^2^School of Information, Kochi University of Technology, Japan Email: shigemasu.hiroaki@kochi-tech.ac.jp

## 

Although previous studies reported depth adaptation of disparity-defined corrugation involved both phase-dependent and independent processing (Graham and Rogers, 1983; He and Shigemasu, APCV, 2016), whether there is any shape-level depth adaptation is still unclear. Using dynamic random-dot stereograms in phase randomly-changing adapting condition, we compared the depth aftereffects with the horizontally orientated sinusoidal corrugations, plaids and noise-shape as adaptation stimuli separately and horizontally orientated corrugations as test stimuli. Three adaptors had the same amount of disparity but different distributions. The plaids were composed of horizontally and vertically orientated corrugations, which had the same peak-to-trough amplitudes. The dots of noise-shape adaptor were distributed in random positions without exact shape structure. In each condition, after adapting to two horizontally- positioned adaptors with larger-smaller or middle-middle amplitudes separately, participants were asked to judge which side of the test stimuli had larger amplitude, and PSE was calculated. Results showed significant differences among three conditions. The horizontally orientated adaptor had the same shape as test stimuli and caused the largest aftereffects. The plaid adaptor caused the middle amount of aftereffects. The noise-shape adaptor had totally different shape and caused the smallest amount of aftereffects. These results might suggest the shape-level depth adaptation, which was independent of disparity-specified depth adaptation.

## Keywords

binocular disparity, stereopsis, shape perception, depth adaptation

## Can “Mean Luminance Deprivation” Modulate Ocular Dominance Plasticity?

**Jiawei Zhou^1^, Zhimo Yao^1^, Yonghua Wang^1^, Jia Qu^1^ and Robert Hess^2^**

^1^Wenzhou Medical University, China

^2^McGill University, Canada Email: zhoujw@mail.eye.ac.cn

## 

If one eye is patched for a period of 2.5 hr, transient changes in ocular dominance result with the previously patched eye’s contribution being strengthened. Similar changes result from opaque and translucent occlusion suggesting that it is the form information not the luminance information that drives these neuroplastic changes. However, this does not rule out the possibility that interocular luminance imbalances per se cannot produce changes in ocular dominance, indeed based on what we know of the physiology, where the contrast gain of visual neurons is luminance dependent, one would expect it can. We show that if the mean luminance of one eye is reduced 1,000-fold for a period of 2.5 hr, there are subsequent changes in ocular dominance and that this critically depends on the absolute luminance of the deprived eye rather than the relative interocular imbalance. We argue that this suggests that the site of action is subcortical, before binocular combination.

## Keywords

ocular dominance, luminance, monocular deprivation

## Acknowledgments

This study was supported by NSFC 81500754 and QTJ16005 to JZ and CIHR grants MOP-53346, CCI-125686 and MT-10818 and an ERA-NET NEURON (JTC 2015) to RFH.

## Individual Differences in Lower and Upper Limits of Disparity Detection for Depth Perception

**Hirohiko Kaneko^1^, Atsumi Momose^1^, Masayuki Sato^2^ and Kei Kanari^1^**

^1^Tokyo Institute of Technology, Japan

^2^The University of Kitakyusyu, Japan Email: kaneko.h.ab@m.titech.ac.jp

## 

Horizontal disparity, that is, horizontal difference between images seen by two eyes, is a cue for depth perception. There are individual differences in the processing of binocular disparity for depth perception even for people who have stereoscopic vision. To investigate the causes for individual differences in stereopsis, we measured stereo acuity with appropriate optical corrections, eye refractive state with no optical corrections and personal refractive state over the years to assess the effects of the latter two factors on stereo acuity. We also measured upper limit to detect disparity for depth perception. Results revealed that stereo acuity for myopic participants was significantly higher than that for emmetropic or hyperopic participants. Participants with a large horizontal astigmatic power tended to have low stereo acuity. Further, upper limit to detect disparity was correlated to stereo acuity. Participants to have high upper limit tended to have low lower limit to detect disparity (high stereo acuity). We discuss these results in terms of individual differences in the quality of retinal image and personal experience of space environment.

## Keywords

stereopsis, disparity, individual difference, refractive state, binocular vision

## Acknowledgments

This study was supported by JSPS KAKENHI (grant Number 15H02725).

## Effect of Inter-Ocular Contrast Ratio on Perceived Depth From Disparity

**Pei-Yin Chen and Chien-Chung Chen**

Department of Psychology, National Taiwan University, Taipei, Taiwan Email: c3chen@ntu.edu.tw

## 

The perceived depth from disparity in random dot stereogram can be affected by luminance contrast (Chen, Chen & Tyler, 2016). Here, we further investigated whether interocular contrast difference can influence the perceived depth from disparity. The test stimuli were rectangular random dot stereograms (1.27 × 3.44 deg) whose binocular disparities were modulated horizontally to generate the percept of a single cosine surface (0.29 cycle/deg). The maximum test disparity ranged from 0 to 20 arc min, while the luminance contrasts of the left- and right-eye image were assigned independently from 5% to 80%. The observers’ task was to adjust the length of a horizontal bar to match the perceived depth difference in the test stimuli. When the interocular contrast ratio (C (High)/C (Low)) was small, the perceived depth increased with the luminance contrast in either eye. However, when the interocular contrast ratio was larger than 4, the perceived depth decreased with the interocular contrast ratio. The effect was the same regardless where the higher contrast was placed in the dominant eye or not. Thus, the perceived depth from disparity depends on not only luminance contrast but also interocular contrast ratio in the stimuli.

## Keywords

depth perception, binocular disparity, interocular interaction, binocular vision

## Acknowledgments

This study was supported by 103-2410-H-002-076-MY3

## Evaluation Consistency and Image Statistical Analysis on Skin Transparency

**Yuna Nakanishi^1^, Takanori Igarashi^2^ and Katsunori Okajima^1^**

^1^Yokohama National University, Japan

^2^Kao Co. Ltd., Thailand Email: okajima@ynu.ac.jp

## 

“Skin transparency” has been used as a popular evaluation term for human facial skin in Japan. However, there is no common definition on “transparency” of skin because it relates to individual personal impressions. In this study, we confirmed the consistency between numerical values of the skin transparency obtained by using a magnitude estimation and a paired comparison methods. In addition, we investigated the relationships between the skin transparency and the image statistical values of the skin. We conducted a pairwise comparison experiment and a subjective evaluation experiment on skin transparency. In the two experiments, participants and stimuli were identical. Eight young students participated and 14 skin patches of women with different ages were used as visual stimuli. As a result, strong correlations between results of the two experiments were shown, suggesting that there is a common and consistent scale of skin transparency and that the subjective evaluation of the skin transparency is an effective evaluation method of the skin appearance. Furthermore, we analyzed the correlations between the skin-transparency and image statistical values. As a result, it was found that the mean luminance value of skin is strongly correlated to the skin transparency.

## Keywords

skin transparency, image statistics, magnitude estimation, paired comparison

## Effect of Spatial Structure Defined by Disparity With Uniform Luminance on Lightness

**Kei Kanari and Hirohiko Kaneko**

Tokyo Institute of Technology, Japan Email: kei.kanari@ip.titech.ac.jp

## 

It has been shown that spatial structure of the scene affects lightness of an object. However, most of the previous studies focus on the context of the luminance distribution surrounding the object. Our visual system could estimate illumination of a scene from a surrounding spatial structure based on a correlation between the scene and illumination. For example, an object does not receive light from above when there is something to shield above the object. This study investigated whether lightness is influenced by the spatial structure with no explicit information of luminance. The stimulus consisted of random dots with 3D structure defined by binocular disparity which was used to eliminate the influence of luminance and texture. The magnitude of recognized illumination was manipulated by changing the spatial structure of the scene. Observers responded to the lightness of the test patch presented in the stimulus space by adjusting the luminance of the comparison patch placed outside the scene. Results showed that the responded luminance increased when the test patch was interpreted to receive weak illumination. This result suggests that the visual system can infer the illumination of a scene from spatial structure with no information of luminance distribution.

## Keywords

lightness, brightness, binocular disparity, spatial recognition

## Would the Phenomenon of 'The Dress' Exist in Simple-Patterned Picture?

**WanYu Chen and Shojiro Sakurai**

Kaohsiung Medical University, Taiwan Email: sakurai@kmu.edu.tw

## 

The dress photograph, which is posted on the Tumblr in Feb 2015, had produced an interesting phenomenon of visual perception. Some people claim that’s the combination of blue and black color, some people see that’s the combination of white and gold color, and some people’s perception change between the two combinations. The purpose of this study is to create a picture, which have the same effect as the dress, can divide people into three types of color combinations. In this study, we use two online questionnaires. The first one contains two dress pictures: one is partial and the other is whole; the second one contains 22 blue-black stripped pictures. In the first questionnaire, the result shows that 55 of 268 participants perceive whole dress picture as white and gold color, the rest perceive blue and black. In the white-and-gold groups, 24 of 55 participants perceive white and gold in the partial picture, 22 of 55 participants perceive blue and black. In the second questionnaire, the result shows that 6 of 30 participants only see blue and black in stripped pictures. Moreover, the top five pictures, that are ranked by different groups separated by the perception of partial picture, are the same.

## Keywords

individual difference, color perception, The Dress

## Consideration of Relationship Between Word Impression and Color Impression Using Color-Paired Comparison Method

**Honami Komatsu^1^ and Keizo Shinomori^2^**

^1^Graduate School of Engineering, Kochi University of Technology, Japan

^2^School of Information, Research Institute, Kochi University of Technology, Japan Email: honami.komatsu@gmail.com

## 

We investigated the relationship between word impression and color impression by selecting hue for the word in color-paired comparison method, in which two colors were simultaneously presented from 132 combinations and an observer selected the one color closer for the impression of an evaluating word. In the experiment, six trials for each combination were performed and 10 subjects participated. We selected the evaluating words by referring the results of the factor analysis to the data in the previous literature. Twelve surface colors from vivid tone in the PCCS were represented under D65 on a monitor with a luminance of 77.2 cd/m^2^. The results of the selection were converted to z-score, and the principal component analysis on the averaged z-score of each color for the words was performed. Consequently, the first and second principal components had 95% cumulative contribution rate and more than 75% of model predicted values by the linear combination of the two components were within the 95% confidence interval. In addition, plots of each color distributed elliptically in the scatter diagrams with axes of the two principal components. Therefore, those 12 colors are handled according to hue impression in expressing word concepts.

## Keywords

word impression, color impression, paired comparison

## Acknowledgments

This study was supported by Priority Research Laboratory of Kochi University of Technology.

## Which Regions in the Human Brain are Involved in Lightness Perception?

**Yuichi Sakano, Yoshiaki Tsushima, Atsushi Wada and Hiroshi Ando**

NICT & Osaka University, Japan Email: yuichi@nict.go.jp

## 

We conducted an fMRI experiment to clarify which regions in the human brain are involved in lightness perception. The stimulus was a flat surface placed on a checkerboard. The surface was light or dark gray and was or was not shadowed by a cylinder placed behind the viewpoint. The surface also had variations with two levels in form and orientation. The stimuli were presented successively in pair, where the paired stimuli were different in terms of whether the surface was shadowed or not in the half of the pairs. The subjects were asked to report whether the paired surfaces were the same or different in terms of lightness, form, or orientation while the stimuli used in those three tasks were identical. If certain regions are more activated during the lightness discrimination task than another task, the regions would be involved in lightness perception. We found that a region in the vicinity of the calcarine sulci was more activated in the lightness task than in the form task. This result indicates that the low-level visual region such as the primary visual cortex is involved in lightness perception.

## Keywords

lightness perception, lightness constancy, fMRI

## Acknowledgments

This study was supported by JSPS KAKENHI (grant Number 16H02826) and Keihanna Research Complex (JST).

## Enhanced Saturation Contrast Caused by Saturation Gradients

**Yuki Kobayashi^1^, Soyogu Matsushita^2^ and Kazunori Morikawa^1^**

^1^Osaka University, Japan

^2^Osaka Shoin Women's University, Japan Email: y-kobayashi@hus.osaka-u.ac.jp

## 

A gray patch surrounded by a white area appears darker than that surrounded by a black area. This “simultaneous lightness contrast” effect is enhanced when luminance gradients from black (outer part) to white (inner part) are added in the outer region of the surrounding white area (Agostini & Galmonte, 2002). This fact shows the effect of luminance gradients on lightness perception. Then, how about chromatic saturation gradients? It is known that simultaneous saturation contrast occurs in addition to lightness contrast. In this study, we examined if saturation gradients enhance saturation contrast effect. We employed stimuli containing isoluminant saturation gradients and asked participants to perform a chromatic saturation matching task using the method of adjustment. The results demonstrated that saturation gradients enhance simultaneous saturation contrast in the same way that luminance gradients do lightness contrast. Although the relationship between the effects of saturation gradients and luminance gradients needs to be clarified, we assume that the results of this study can be attributed to the albedo hypothesis that Agostini and Galmonte (2002) adopted.

## Keywords

color, psychophysics, contrast, gradient, albedo

## Retinal Mechanism

## Encoding the Light Intensity in Retina’s Firing

**Jo-Fan Chien, Kevin Sean Chen, Yu-Ting Huang, Chun-Chung Chen and Chi Keung Chan**

Academia Sinica, Taiwan Email: jofan8067@gmail.com

## 

A retina receives light stimulation and transforms the detected signal into spikes, which are then transmitted to the brain. However, how light stimulation information is conveyed by spike trains is not fully understood. Here, we utilize a multi-electrode array to record firings of the bullfrog’s retinal ganglion cells under a whole field stochastic light stimulation. Our goal is to understand the mutual information shared between stimulus light intensity and the firing rate of RGCs. The result from the stimulation with different correlation times shows that if the correlation time is longer, the retina takes shorter processing time. Furthermore, the retina can even detect the hidden variable in the stochastic stimulation to make prediction. We utilize the time-shifted mutual information to quantify the information encoded in the firing rate and used it as an interpretation of prediction.

## Keywords

retina, multi-electrode array, mutual information, prediction

## A Temporal Difference Between Cone- and Melanopsin-Mediated Signals in Pupillary Pathway

**Wakayo Yamashita and Sei-ichi Tsujimura**

Kagoshima University, Japan Email: tsujimura@ibe.kagoshima-u.ac.jp

## 

There is a third class of photoreceptors in addition to cones and rods in primates. The photoreceptive retinal ganglion cells (pRGCs) containing photopigment melanopsin are known to support various non-image forming functions including circadian rhythm and pupillary light reflexes. In physiology, several studies have shown that pRGCs have a long latency to light stimuli, much slower than that for cones. The slow response seems ecologically advantageous in terms of irradiance encoding of environmental light. Although the pRGCs in the retina are slow, signals originated from pRGC in entire pupillary pathway are unclear. The aim of this study is to clarify a difference in time between cone-mediated fast signals and pRGC-mediated slow signals in the pupillary pathway. We used test stimuli that consist of cone and pRGC stimuli. The cone stimulus modulated cones alone, while pRGC stimulus modulated pRGC alone. These two stimuli were summed with various temporal phases in order to estimate an intrinsic temporal difference between cone- and pRGC-mediated signals. The results showed that an amplitude of the pupillary response was largest to the test stimuli in which the phase shift of the pRGC stimulus was advanced by about 1.7 s from the cone stimuli.

## Keywords

melanopsin, cone, phase shift, silent substitution, pupillary response

## Acknowledgments

This study was supported by JSPS KAKENHI (grant Numbers JP26280103, JP26540146).

## Pathway Analysis Implicates Altered Mitochondrial Metabolism, and Neurotransmission and Complement Cascade in Retina/RPE/Choroid in Form-Deprivation Myopia

**Sheila Crewther, Loretta Guimmarra, Nina Riddell and Melanie Murphy**

La Trobe University, Australia Email: s.crewther@latrobe.edu.au

## 

Recent RNAseq analysis has demonstrated bidirectional changes in neurotransmission and metabolism, structural and immune pathways during induction of optically induced myopia. Thus, the aim of this study was to investigate whether similar gene pathways are also related to the excessive axial growth during the induction and recovery from form-deprivation myopia (FDM) in chick. Gene set enrichment analysis software was used to determine enriched pathways in both archived (accession # GSE6543) genomic transcriptome data and new FD recovery data correlated with biometric data. Significant changes in mitochondrial energy metabolism, neurotransmission, G protein-coupled receptor signalling and complement cascades were identified during the10 days of induction of profound myopia and found to correlate well with change in axial dimensions. Bile acid and bile salt metabolism pathways (cholesterol/lipid metabolism and sodium channel activation) were significantly upregulated during recovery. The pathways altered during induction of FDM are established indicators of oxidative stress and are consistent with the choroidal thinning, axial elongation and hyperosmotic ion distribution patterns across the retina and choroid previously reported in FDM.

## Keywords

Form deprivation myopia, pathway analysis, retina/RPE/choroid

## Acknowledgments

This study was supported by National Health and Medical Research Council Development (ID448606) to DPC and SGC and a further Australian Research Council (DP110103784).

## Spatiotemporal Integration of Visual Stimuli in the Divisional Power Supply Scheme of the Retinal Prosthesis

**Yueh-Chun Tsai^1^, Bo-Jyun Lin^2^, Pin-Shiou Wang^2^, Ching-Hsiang Liu^2^ and Huan-Chin Chiao^1^**

^1^Institute of Systems Neuroscience, National Tsing Hua University, Hsinchu, Taiwan

^2^National Experimental High School at Hsinchu Science Park, Hsinchu, Taiwan Email: remhom@gmail.com

## 

The power-free photovoltaic retinal prosthesis is currently under studied aiming to restore vision of patients with retinitis pigmentosa and age-related macular degeneration. The major challenge of the photovoltaic device is the limitation of power efficiency. In collaboration with Prof. Chung-Yu Wu at the National Chiao Tung University, our photovoltaic prosthesis implements a unique divisional power supply scheme (DPSS) system, which provides electricity to only a subset of electrodes at any moment in time with the power generated by all solar cells. This design significantly increases the power efficiency for each electrode, but could potentially reduce the spatiotemporal resolution of retinal prosthesis in human patients. The present study was to systematically characterize the performance of spatiotemporal integration in various DPSS conditions for human subjects using a psychophysical approach. A 16 × 16 pixels LED array controlled by Arduino was used to simulate the output signal of the DPSS design, and human performance corresponding to different visual stimulations at various update frequencies was used to assess the spatiotemporal resolution of retinal prosthesis. The results showed that the visual angle, pattern complexity and familiarity, division configuration, and contrast polarity significantly influence the optimal update frequency of the DPSS system, while the division number and stimulation order apparently do not affect the performance. These findings provide an insight into the optimization of the photovoltaic retinal prosthesis with the DPSS design, which could be developed into a power-free device able to restore vision in the future.

## Mild Stress Promotes Neurite Outgrowth of Retinal Explants

**Grace Chen^1^ and Chuan-Chin Chiao^2^**

^1^Department of Life Science, National Tsing Hua University, Hsinchu, Taiwan

^2^Institute of Systems Neuroscience, National Tsing Hua University, Hsinchu, Taiwan Email: ccchiao@life.nthu.edu.tw

## 

In our previous experiment, we discovered that the slightly increased culture medium concentration as a result of the elevated evaporation rate can significantly promote neurite outgrowth of ganglion cells in mouse retinal explants. To verify this observation, the overall concentration of the culture medium was increased 1.25 and 1.5 folds. The results showed that the 1.25×, but not 1.5×, increased concentration can significantly enhance neurite outgrowth. However, the elevated concentration of culture medium not only increases the relative amount of forskolin but also changes the osmolarity of the medium. This elevated osmotic concentration in the culture medium may stimulate the neurite outgrowth by turning on the stress compensation mechanism. To verify this hypothesis, overall temperature increase from 35 to 38°C for 1 hr each day (mild stress) and constant exposure at 38°C for five days (strong stress) were used to assess its effect on neurite outgrowth. The results showed that the neurite outgrowth under the mild stress condition is much better than under the control and strong stress conditions. These findings provide an insight into the cellular mechanism of retinal axon regeneration under the stress condition, which could potentially develop into a new method in neural regeneration.

## Keywords

retina, neurite, mild stress

## Visual Cognition

## A New Paradigm for Studying Inter-Ocular Competition With Amplitude Modulated Flicker

**Victor Lee, Kien Nguyen, Wen-Sheng Chang, Wei-Kuang Liang and Chi-Hung Juan**

National Central University, Taiwan Email: chihungjuan@gmail.com

## 

Dichoptic stimulation using frequency-tagged flicker has been used as a tool to observe the change of response at the cortical level. Nevertheless, studies of the flickering effect in the inter-ocular competition of the human visual system are limited. This is because even though correlations between the stimulus in one eye affecting the other eye’s network are observed in SSVEP responses, it has been challenging to confirm any causality. The observed amplitude-coupled SSVEP response might in fact be a coincidental event due to the lack of time control. Therefore, we designed a new paradigm for detecting if indeed there are inter-ocular inhibitions by simultaneously combining amplitude modulation (AM) with a sine wave stimuli from the other eye. The amplitude fluctuations from the AM signal provides the time control manipulation for verification of any interaction between monocular neurons. We found a modulation from AM stimuli inhibiting the 25 Hz sine wave in a phase-dependent manner. Moreover, AM stimulation originating from the dominant or non-dominant eye did not interfere in the modulation of 25 Hz suggesting that AM signal stimuli is eye dominance independent. This is the first time that a tool has been developed for revealing causality in inter-ocular competition studies.

## Keywords

SSVEP, amplitude modulation, dichoptic stimulation, inter-ocular competition

## Acknowledgments

This study was supported by Ministry of Science and Technology (MOST).

## Visual Model Shows That Activity Retrieved From Memory Could Resemble Sensory Responses Despite Decay

**Thomy Nilsson**

University of Prince Edward Island, Canada Email: nilsson@upei.ca

## 

After viewing a hue, matches made from memory produce difference thresholds as a function of wavelength which closely resembled hue difference thresholds obtained by simultaneous matching. This resemblance persisted despite a negative exponential decay of matching accuracy over time. These results were later replicated, and similar results found to occur with memory difference thresholds for orientation of single lines and gratings. Since sensory difference thresholds reflect sensory responses, this similarity suggests that what is retrieved from memory resembles sensory responses. If sensory responses are stored in memory and subject to random decay, can their characteristics endure sufficiently to produce memory difference-thresholds that resemble sensory functions?

This question was tested with a neural network model of memory matching using stored sensory patterns subject to increasing noise being correlated with stimulus patterns that varied from and included the original stimulus. The model demonstrates that difference-threshold functions obtained with noise increasing over time could continue to resemble functions obtained without noise. This suggests that when multiple matches are made from memory to measure difference thresholds, their averaging reveals sensory activity stored within accumulating noise.

## Keywords

vision, memory, hue, grating orientation, difference thresholds

## Acknowledgments

This study was supported by National Science Engineering Research Council—Canada.

## A New Method to Quantify Visual Response Latency With Steady-State Visually Evoked Potentials in Human

**Kien Nguyen, Victor Lee, Wen-Sheng Chang, Wei-Kuang Liang and Chi-Hung Juan**

National Central University, Taiwan Email: chihungjuan@gmail.com

## 

In human visual research, measuring the visual response latency by using steady state visually evoked potentials (SSVEPs) to identify which peak of response corresponds to which peak from the stimuli signal is still challenging and inaccurate. To overcome the drawback, this study developed a new method using amplitude-modulated visual stimulation generated by an LED flickering to induce the SSVEP response. To assure that the amplitude-modulated waveform is correctly generated and to measure the onset time of the LED, a photodiode was attached with the LED. The advantage of this method is that the envelope of the SSVEP signal can be extracted to then compare its peaks with the corresponding peaks of the envelope of the photodiode signal for gauging the response latency. The results demonstrated that the SSVEP power strongly distributed in the occipital lobe showing a response latency (signal from Oz channel) following single-eye and dual-eye stimulation of approximately 100 ms (range from 90 to 120 ms). Furthermore, SSVEP responses in anterior channels (e.g., Cz and Fz channels) had longer latencies (130–160 ms) than those in the Oz channel. In addition, no significant difference was observed between the latency from the dominant and non-dominant eye.

## Keywords

SSVEP, amplitude modulation, LED flicker, visual response latency

## Impact of Putamen and Thalamus Lesions on Oddball P300 Generation

**Yi-Min Tien^1^, Li-Chuan Hsu^2^, Sui-Foon Lo^3^ and Chia-Yao Lin^2^**

^1^Department of Psychology, Chung Shan Medical University, Taiwan

^2^School of Medicine, China Medical University, China

^3^Department of Physical Medicine and Rehabilitation, China Medical University Hospital, China Email: tien@csmu.edu.tw

## 

The oddball paradigm combined with recording of event-related potential (ERP) can elicit the P300 waves. Researchers suggested that P300 reflects attention and memory functions. Although the generator sites are still unclear, the subcortical structures, such as the locus coeruleus and the thalamus, play an important role. The aim of the present study was to investigate the effects of different brain lesions on the P300 component. We recorded the P300 component by using visual and auditory oddball paradigms. Patients with Putaman or thalamic stroke were recruited as participants. Healthy young and age-matched participants were also included as control groups. Patients underwent full clinical examination and MRI scan. All participants accepted the Mini-Mental State Examination (MMSE) for evaluation of general cognitive functions. Decreased visual P300 peak amplitude was also found in patient groups as well as in age-matched group suggesting an aging effect. Compared to young and age-matched healthy controls, patients with Putamen or thalamus stroke showed delayed auditory P300 peak latency. It suggests the impairment of the auditory P300 may stem from lesions at various locations and different lesion types. Our study illustrates the important role of corticolimbic structures in the generation of the P300 potentials.

## Keywords

oddball paradigm, putamen, thalamus, P300

## Acknowledgments

This study was supported by MOST105-2410-H-039-002; MOST103-2410-H-039-001-MY2.

## Transfer of Multi-Attribute Stimulus-Response Mappings

**Yumiko Fujii, Masahiko Morita and Hiromi Morita**

University of Tsukuba, Japan Email: yfujii@slis.tsukuba.ac.jp

## 

The human visual system processes different attributes of an object separately and then integrates them to elicit a specific response. Based on the paired-attribute model in which bound feature pairs are units of multi-attribute stimulus-response association, our previous study suggested that learning the association of a color–location pair or a shape–location pair with a response is more difficult than that of a color–shape pair. Present study investigated on which pairs the association of a stimulus comprising color, shape, and location with a response depended upon. Participants learned mapping of eight items comprising color (red/green), shape (circle/triangle), and location (left/right) to four responses. Thereafter, they were tested with transfer blocks with a pattern comprising a colored shape and a colored switch indicating left or right (condition of two pairs), a pattern comprising a colored shape and a gray switch (condition of a color–shape pair and a location singleton), and a pattern comprising a gray shape and a colored switch (condition of a color–location pair and a shape singleton). The result revealed that correct response rate dropped only for color–location pair and shape singleton conditions, suggesting that color–shape pair is more important in stimulus–response association than the color–location pair.

## Keywords

visuomotor mapping, object vision, feature binding

## Acknowledgments

This study was supported by JSPS KAKENHI (grant Number 26590173).

## Individual Difference in Statistical Learning of Dependency Between Nonadjacent Visual Shapes in Sequence Correlates With Sentence Reading

**Kunyu Xu, YuHuei Lian and Denise Wu**

National Central University, Taipei Email: kunyu.xu@gmail.com

## 

Increasing evidence has shown that the ability of statistical learning (SL) contributes to the acquisition and processing of individual words in people’s native and foreign languages. However, whether such ability contributes to sentence reading is less explored. The present study examined whether individual difference in SL, as indexed by participants’ sensitivity to nonadjacent visual shapes in sequence, correlates with their ability in reading sentences with relative clauses (RCs). Previous research on Chinese sentence processing has established that sentences with subjective RCs (SRCs) are more difficult to read and to comprehend than those with objective RCs (ORCs). The present results replicated the SRC disadvantage in a self-paced reading task, and further showed that individual difference in the magnitude of such disadvantage, as measured by online reading time especially at the head noun position, significantly correlated with participants’ performance in the SL task. On the other hand, individual difference in the magnitude of SRC disadvantage did not correlate with participants’ IQ or working memory. These findings suggest that SL of dependency between nonadjacent visual shapes might serve as the underlying mechanism to support acquisition and processing of linguistic structures in written sentences.

## Keywords

visual statistical learning, working memory, sentence processing

## Acknowledgments

This study was supported by MOST 105-2420-H-008-001-MY3.

## How Scene Changes Influence Eye Movements

**Esther X. W. Wu, Shih-Cheng Yen and Fook-Kee Chua**

National University of Singapore, Singapore Email: estherwu@nus.edu.sg

## 

According to the film editor, Walter Murch, the viewer is compelled to re-evaluate the novel scene context following a scene cut when the scene change is large. However, when the scene change is small, viewers may notice the scene change, but the novel scene may not be different enough to compel viewers to re-evaluate the scene context. Yet, the failure to re-evaluate the scene context could also be due to the viewer not being aware of a scene change. In this study, we conducted two experiments to (a) examine how the magnitude of scene change influences the re-evaluation of the novel scene, indicated by the initial centering response (ICR) and (b) examine how the ICR relates to viewers’ conscious awareness of a scene change. In Experiment 1, pairs of images were presented for several seconds, one after the other, separated by a brief mask (15 ms), as image difference was varied. We found that the ICR increased with the magnitude of scene change in the image pairs. In Experiment 2, we further asked participants to respond to the scene change. Our results suggest that viewers were aware of a small scene change even if they did not re-evaluate the novel scene.

## Keywords

scene changes, eye movements, centering

## Attention

## Visual Attention Differences in the Broader Autism Phenotype

**Alana Cross^1^, Robin Layckock^2^ and Sheila Crewther^1^**

^1^La Trobe University, Australia

^2^RMIT University, Australia Email: 15762187@students.latrobe.edu.au

## 

Visual attention is known to vary across the Broader Autism Phenotype (BAP), although this relationship has not yet been adequately explored. Attentional deficits are associated with the emergence of autism characteristics, including communication and social problems. Fifty children aged between 5 and 12 years completed The Attention Network Task (ANT) to examine Posner’s attentional networks—alerting, orienting and executive control. Their performance was compared on an inspection time (IT) task and a parent-rated Autism Spectrum Quotient (AQ-Child) questionnaire. An overall correlational analysis showed that IT performance was associated with reaction time (RT) for all congruent cued conditions but not incongruent. Attention to detail, measured on the AQ-Child, was associated with RT on ANT incongruent cued conditions only. Orienting and Executive Control networks were correlated on the ANT. When split into high and low AQ groups, there was a group difference in mean accuracy on incongruent cued conditions of the ANT. No group differences were found in RT or congruent conditions. High AQ traits appear to be associated with less flexibility in changing attention. In line with autism research, Posner’s attentional networks may not be independent of each other in the BAP. Further, parent-reported behavioural traits can relate to phenotypic visual processing characteristics.

## Keywords

attention, autism, perception

## The Influence of Invisible Local Information on the Integration of Global Form and Motion Coherence

**Charles Chung and Sieu Khuu**

The University of New South Wales, Australia Email: charles.eric.chung@gmail.com

## 

In this study, we examined whether the integration and detection of global form and motion relies on local information that is made progressively made invisible using continuous flash suppression (CFS). Global motion and form coherence thresholds were measured using variants of the global dot motion (GDM) and Glass pattern stimulus in which the signal to noise ratio was varied (using a staircase procedure targeting the 79 % correct performance level) until the pattern can be just detected. The stimulus was presented using a two-interval forced choice design in which the task was to indicate the interval containing the global pattern (defined by motion or form). Across conditions, spatial sectors of the stimulus (0, 25, 50, 75%) were suppressed using CFS, and we determined whether this affected form and motion coherence thresholds. We found that increasing the areas of suppression in the stimulus did not affect coherence thresholds with performance similar to when the entire stimulus was visible to the observer. These findings suggest that visual awareness is not a requirement for form and motion integration, and importantly that unconscious information continue to influence conscious perception.

## Keywords

Continuous flash suppression, awareness, global dot motion, Glass pattern, motion

## The Aging Effect on Time Perception: An ERP Study

**Hsing-Hao Lee and Shulan Hsieh**

Department of Psychology, National Cheng Kung University, Taiwan Email: joe26272001@gmail.com

## 

Time perception function is indispensable in our daily lives. Time perception is influenced by many cognitive functions which are also affected by aging. Current study collected event-related potential (ERP) data, while participants were performing a temporal bisection task to investigate the aging effect on time perception. We used contingent negative variation (CNV), as well as the behavior response as the indices of time perception ability. We found that elders’ CNV peak latency was significantly shorter than that of younger adults. Besides, CNV peak amplitude was also smaller for elders than younger adults. Both results indicated that there exists aging effect on time perception function: Elders’ pacemaker operates at the different rate as younger adults’ does. This is the first study using ERP approach to probe into the issue of time perception and aging, which has verified the differences of timing function in younger and older adults through both behavioral and electrophysiological measurements.

## Keywords

time perception, contingent negative variation, aging

## Acknowledgments

This study was supported by the Ministry of Science Technology (MOST) of Taiwan, R.O.C.

## Visual Perception in Peripheral Visual Field Is Modulated by Eccentric Gaze

**Ryoichi Nakashima**

The University of Tokyo, Japan Email: rnaka@l.u-tokyo.ac.jp

## 

Visual perception can be altered by head direction even when gazing at the same location (Nakashima & Shioiri, 2015), such that perception is facilitated during eccentric gaze when a visual stimulus appears anterior to the direction of the head. The effect of eccentric gaze, where the gaze direction is not aligned from the head direction, on visual perception at central and peripheral vision was investigated. Participants were asked to judge the orientation of “T” presented in central vision (central task) and simultaneously detect a dot appearing in peripheral vision (peripheral task). The main manipulation was gaze direction; frontal vs. eccentric. Results indicated that the performance of the central task did not differ based on gaze condition. Moreover, the peripheral task performance did not differ when the dot appeared near the fixation. In contrast, the effect of gaze was recognized when the dot appeared far from fixation, such that dot detection was superior when it appeared to the left (right) of fixation during a right (left) eccentric gaze. This finding suggests that visual perception is facilitated approximately in a direction anterior to the head. We concluded that eccentric gaze influences visual perception, particularly in peripheral rather than in central vision.

## Keywords

head direction, eccentric gaze, visual perception, visual attention, peripheral vision

## Shared and Distinct Information Processing Limitations Across Attentional Forms and Modalities

**Gwenisha J. Liaw, Takashi Obana, Tiffany T. Y. Chia and Christopher L. Asplund**

Singapore Institute for Neurotechnology, Singapore Email: chris.asplund@yale-nus.edu.sg

## 

Selective attention allows us to prioritize which sensory information reaches awareness. In both the visual and auditory domains, attention controlled either voluntarily (goal-directed) or by external events (stimulus-driven) has a dark side: Unattended items are frequently missed. The extent to which these attentional limitations are due to common cognitive mechanisms, however, is not fully understood. In this study, we adopted an individual differences approach to investigate the relationships amongst temporal attentional capacity limitations. The Attentional Blink (AB) indexed goal-directed attentional limitations, whereas Surprise-induced Blindness (SiB) and its auditory analogue surprise-induced deafness (SiD; Obana & Asplund, in prep) indexed stimulus-driven ones. Each participant (*n* = 75) was tested twice on each paradigm in each sensory modality, thereby allowing us to calculate cross-task correlations and test–retest reliability. Despite finding strong test–retest reliability and weaker, yet significant, correlations between blink and surprise deficits within modalities, only SiB and SiD were related across modalities. In contrast, visual and auditory blink magnitudes were uncorrelated. We conclude that goal-directed and stimulus-driven attention may be contingent on partially shared capacity limits within modalities. In addition, shared stimulus-driven deficits across modalities may be due to a central cross-modal-alerting mechanism.

## Keywords

attentional blink, surprise-induced blindness, capacity limitations, visual attention, auditory attention

## Measuring Attentional Facilitation Related to Preparation of Hand Movements

**Takumi Miura, Kazumichi Matsumiya, Ichiro Kuriki and Satoshi Shioiri**

Tohoku University, Japan Email: shioiri@riec.tohoku.ac.jp

## 

Visual processing is enhanced at the goal of hand movement, suggesting existence of visual attention related to action. We used steady-state visual evoked potential (SSVEP) to measure temporal profile of the attentional modulation related to hand movements. Two disks flickering independently were presented on the right and left of the fixation, and participants attended to either of the two disks to perform an RSVP task. While attending to one disk, he/she was asked to move their hand to the disk indicated by a cue (either the attended or not attended disk) from the initial location right below the fixation. The time course of SSVEP revealed that the amplitude increased at about 500 ms before the hand movement and decreased after the start of the movement at the attended disk. No such effect was found at the unattended disk. These results can be interpreted as follows. There is additional attentional modulation at the position to which attention has already been allocated when the position is defined as the goal of hand movement. The attentional effect is shown before the hand movement and disappears during hand movement, perhaps because attention is on the hand in movement rather than the goal of the movement.

## Keywords

attention, motor program, electroencephalogram

## Acknowledgments

Grant-in-Aid for Scientific Research (16K12441).

## Spatial Compression at Peripheral Vision Without Saccades and Visual Masks

**Masahiko Terao and Fuminori Ono**

Yamaguchi University, Japan Email: masahiko_terao@mac.com

## 

It is known that when a stimulus flashed just before saccades, its location appeared to be closer to the saccade target than it actually was. Similarly, it also has been shown that a flashed stimulus was shifted towards a reference when followed by a visual mask. Here, we report a novel spatial compression phenomenon in the absence of saccades and visual masks. In our experiment, two discs horizontally separated by 12 deg center-to-center were flashed simultaneously. Each of them was presented for 50 ms at 5 deg above the fixation point. Observers estimated the position of each disk in relation to a subsequently presented probe stimulus. When two disks were presented in the center region, that is, 0 deg and 12 deg horizontally apart from the fixation point, the perceived position of each disk was close to its actual location. On the other hand, when two disks were presented further from the center, that is, 12 deg and 24 deg horizontally apart from fixation point, the perceived position of each disk was shifted toward each other. This attraction was not observed when two disks were presented individually. This phenomenon might be explained by the positional averaging at peripheral vision.

## Keywords

localization, peripheral vision, position shift, spatial perception

## Perceived Depth and Accommodation

**Harold Hill and Trent Koessler**

University of Wollongong, Australia Email: harry@uow.edu.au

## 

Focusing of the eye at close distances, ocular accommodation, potentially provides information about depth. However, there is little evidence that this source of information is used by the human visual system. We report three experiments using an illusion of three-dimensional depth reversal, the hollow-face illusion, to investigate the relationship between perceived depth and accommodation. In the first two experiments, we found, using laser speckle optometry, that observers accommodate to perceived rather than actual depth when experiencing the illusion despite the availability of closed loop feedback. In the third experiment, we found that a sharp pattern of dots expected to facilitate accommodation was more effective in resolving depth than the equivalent pattern of blurred dots. This is consistent with accommodation influencing, as well as being influenced by, perceived depth. Both observations held for monocular as well as binocular viewing, ruling out the possibility that they were driven by binocular vergence. We interpret the findings as evidence that accommodation is closely linked to perceived depth in a situation where multiple other sources of information about depth are available even if it does not provide reliable information about depth in isolation.

## Keywords

accommodation, depth perception, hollow-face illusion, laser speckle optometry

## A Linear Mathematical Model of Attentional Modulation in Visual System

**Akihiro Masaoka^1^ and Takeshi Kohama^2^**

^1^Graduate School of Biology-Oriented Science and Technology, Kindai University, Japan

^2^The Faculty of Biology-Oriented Science and Technology, Kindai University, Japan Email: kohama@waka.kindai.ac.jp

## 

The visual system obtains information from surrounding environment by moving eyes. Some meaningful information among them are preferentially processed to understand the situation and to decide the next action. This higher order brain function is called the visual attention. Lanyon & Denham (2004) proposed a mathematical model of functional network of the visual area V4, the inferior temporal cortex (IT), the lateral intraparietal cortex (LIP), and the prefrontal cortex (PF). This model can replicate the modulation of visual attention on the responses of network and to perform visual search. However, it is difficult to extend and control this model because of its strong non-linearity. In this study, we propose a mathematical model which is basically described by linear connection of the visual area V1, V4, IT, LIP and PF. The strengths of connection among cortexes are restricted in certain range to avoid the infinitely increasing the level of neural activity. The simulation results show that the proposed model is able to replicate the responses of typical V1 neurons, and the behavior of the model is quite stable because of reduction of non-linearity. This implies that our model is more valid for elucidating the neural mechanism of the visual attention system.

## Keywords

visual attention, mathematical model, attention network, higher order brain function, visual search

## The Neural Activity for Reloading versus Uploading Conscious Representations During Motion-Induced Blindness

**Li-Ting Tsai^1^, Hsin-Mei Sun^2^, Rufin VanRullen^3,4^ and Chien-Te Wu^5,6^**

^1^Taiwan Association for Visual Rehabilitation, Taipei, Taiwan

^2^Department of Cognitive, Linguistic, and Psychological Sciences, Brown University, Providence, RI, USA

^3^Université de Toulouse, CerCo, Université Paul Sabatier, Toulouse, France

^4^CNRS, UMR 5549, Faculté de Médecine de Purpan, Toulouse, France

^5^School of Occupational Therapy, College of Medicine, National Taiwan University, Taiwan

^6^Department of Psychiatry, National Taiwan University Hospital, College of Medicine, National Taiwan University, Taiwan Email: chientevincewu@gmail.com

## 

We previously reported an intriguing illusory temporal reversal, whereby a new stimulus onset (e.g., a dot flash) presented during motion-induced blindness (MIB) triggers an early reappearance of the “perceptually disappeared” target, yet is systematically perceived as occurring after the target reappearance. This illusion implies that the unconscious target representation can be quickly reactivated, with a temporal advantage for its conscious reloading as compared to the conscious uploading of a newly presented visual stimulus. However, the neural correlates behind perceptually reloading an unconscious representation during MIB remain unclear. To address this question, we recorded EEGs, while participants (*N* = 23) performed a modified MIB task in which a probe was presented during a typical MIB condition or during a no-MIB condition. We compared the event-related potentials (ERP) and event-related spectrum perturbation (ERSP) time locked to the probe onset during the typical MIB condition (perceptual reloading) and the no-MIB condition (no perceptual uploading). Our results showed that perceptual reloading was accompanied by a significant increases of high alpha and low beta powers beforehand and a significant increase in ERP amplitudes at 150–250 ms over the parietal–occipital sites afterward.

## Keywords

motion-induced blindness, visual consciousness, electrophysiology

## Acknowledgments

This study was supported by MOST-102-2923-H-002-002-MY3.

## Attentional Capture Is Affected by Upright or Inverted V-Shape

**Po-Pin Lin^1^ and Yang-Ming Huang^2^**

^1^NCKU, Taiwan

^2^FJU, Taiwan Email: yangming.huang@gmail.com

## 

Previous studies indicated V-shape is perceived as threatening, which captures attention in a short time. In this study, the effect of attentional capture elicited by V-shape in the background of emotional facial expression was examined. The mouth of face background is devised of V-shape: the upright V-shape is happy and the inverted V-shape mouth is sad. The target (upright or inverted V-shape) is placed on the mouth. In Experiments 1 to 3, subjects were asked to determine whether the target is upright V-shape or inverted V-shape; the background is upright facial expression in Experiment 1, the background of inverted face was used in Experiment 2, and both faces used in Experiment 3. In Experiment 4, subjects were asked to determine whether the target and the V-shape of background are of congruent orientation or not. The results revealed that background influences task performance, whether the backgrounds are relevant to task; in Experiments 1 to 3, the effect is caused by V-shape of background, and the effect is caused by emotional feature in Experiment 4. To sum up, subjects usually are more attracted by backgrounds than by targets, and it is a complex attentional mechanism when a few visual V-shapes are presented simultaneously.

## Keywords

V-shape, emotion, attention, attentional capture

## Attention-Modulated Interactions Between Statistical Summary Perception and Statistical Learning

**Wen Tai and Tsung-Ren Huang**

Department of Psychology, National Taiwan University, Taiwan Email: tren.huang@gmail.com

## 

To efficiently process overwhelming information from viewing, human visual system can not only compute summary statistics of a scene (e.g., mean size of objects) but also learn statistical regularities in that scene. However, these two automatic, statistical processes have been reported to interfere each other (Zhao, Ngo, McKendrick, & Turk-Browne, 2011, Psychological Science) and the cause of such interference is not entirely clear yet. Here, we propose that the observed interference resulted from a conflict between relatively distributed spatial attention demanded by statistical summary perception and relatively localized spatial attention demanded by statistical learning. We implemented a computational model to illustrate that distributed attention for statistical summary perception could impair statistical learning of local regularities, which, once learned, could capture attention and thus bias estimates of global summary statistics incorrectly toward local statistics. Our computer simulations successfully replicated findings in the statistical learning literature and various mutual interference phenomena reported by Zhao et al. (2011). The proposed model offers insight into how attention may mediate both statistical processes and its prediction—no interference between statistical summary perception and statistical learning of global scene regularities—has been confirmed by our experiment.

## Keywords

attention, ensemble perception, statistical learning, computational model

## Unconscious Perceptual Grouping Modulated by Top-Down Attention

**Shih-Yu Lo**

National Chiao Tung University, Taiwan Email: shihyulo@nctu.edu.tw

## 

Some researchers suggested that perceptual grouping could be modulated by attention, whereas other researchers suggested that perceptual grouping is an automatic process that does not require consciousness. In this presentation, I integrate the two lines of research and demonstrate that perceptual grouping can be modulated by attention, but this modulation effect takes place unconsciously. The participants were presented with a display that contained two central horizontal lines, while a railway-shaped grouping pattern defined by color similarity was presented in the background that normally induced a Ponzo illusion. The task was to judge the relative lengths of two centrally presented horizontal bars. Although the participants were unaware of the railway-shaped grouping pattern in the background, their line-length judgment was nonetheless biased by it. More importantly, this unconscious biasing effect was more pronounced when the railway-shaped grouping pattern was formed by the attended color than an unattended color, indicating an attentional modulation effect on perceptual grouping without consciousness. Also, the attentional modulation effect was dynamic, being significant with a short presentation time but not with a longer one. A model that dissociates the effects of attention and consciousness will be proposed to integrate the results in this presentation.

## Keywords

awareness, perceptual grouping, unconscious perception, feature-based attention, inattentional blindness

## Acknowledgments

This study was funded by Ministry of Science and Technology, Taiwan (grant number: MOST 104-2410-H-009-001).

## Predicting Direction of Motion in Depth by a Model With Lateral Motion Detectors

**Wei Wu, Kazumichi Matsumiya, Ichiro Kuriki and Satoshi Shioiri**

Tohoku University, Japan Email: wuwei57@riec.tohoku.ac.jp

## 

Perception and estimation of motion direction in depth is precise and human can easily catch or avoid a ball approaching the head. Inter-ocular velocity difference (IOVD) is a cue to motion in depth (MID), and it has been shown psychophysically that there is a visual mechanism to analyze IOVD for motion in-depth perception (e.g., Shioiri et al., 2000). A couple of physiological studies revealed that there exist neurons tuned to IOVD signals in monkey MT (Czuba et al., 2014; Sanada & DeAngelis, 2014). We proposed a model of IOVD motion in depth, considering psychophysical and physiological studies in the literature. In order to realize direction selectivity motion in depth, we assume several MID detectors with different tunings in directions in depth. The direction selectivity of motion in depth is required to catch/avoid an approaching ball and there are psychophysical and physiological supports (Beverly & Regan, 1975; Czuba et al., 2014). Each MID detector has inputs from lateral motion detectors (LMDs) of two eyes, and the direction tuning is built based on speed selectivity of LMD. Simulation showed that the model can predict psychological results about motion in depth to some extent.

## Keywords

motion in depth, inter-ocular velocity difference, lateral motion detector, speed selectivity

## The Effect of Attentional Focus on Motor Learning in a Mirror Drawing Task

**Shi-Sheng Chen and Li Jingling**

China Medical University, China Email: jili@mail.cmu.edu.tw

## 

Previous studies showed that external focus, for example, focus of attention on the consequence of actions, can enhance motor learning. Nevertheless, whether the same locus of attention works on novice is still under debates. This study aims to introduce the mirror drawing task to test the effect of attentional focus. The mirror-drawing task is unfamiliar to most people in daily life and heavily relies on eye–hand coordination, which is a perfect task to ensure novice experience. Two groups of participants (*n* = 20), one for external focus and the other for internal focus, are recruited. They are all right-handed (according to Edinburgh Handedness Inventory) males, aged between 20 and 25 years old. The instruction was delivered to ensure the participants use external or internal focus of attention during mirror drawing. Results showed that the internal group have longer first trial (140.06 s) than that of the external group (100.74 s), while both groups showed significantly improvement at the second trial (120.40 vs. 71.26 s). Our data suggested that the external focus improved motor learning better than internal focus even when the participants are novice to the task.

## Keywords

external focus, internal focus, Edinburgh Handedness Inventory, mirror drawing, novice

## Acknowledgments

This study was supported by MOST103-2628-H-039-001-MY3.

## Multisensory Perception

## Shift of Visual Attention to the Illusory Hand Location

**Moe Nonomura, Chia-Huei Tseng, Kazumichi Matsumiya, Ichiro Kuriki and Satoshi Shioiri**

Tohoku University, Japan Email: shioiri@riec.tohoku.ac.jp

## 

Recent studies have suggested the enhancement of visual processing in peri-personal space. We examined whether attention directs to the illusionary hand location in the condition where subjects perceived their hand illusory at a location different from real hand, using a disappearing hand trick (DHT, Newport et al., 2011). We used flash-lag effect (FLE) to measure attentional effect. FLE is the effect where a flash stimulus aligned with a moving stimulus is perceived as displayed behind; the FLE is affected by attention (Shioiri et al., 2010). The disappearing hand trick is a virtual reality technique to produce perceptual misregistration between visual and proprioceptive information of one’s hand. If the misregistration influences visual attention, attentional modulation on FLE would be found around the perceived hand rather than real hand. In addition to illusory condition, there was a control condition, where no illusion was used and subjects perceived their hand at the actual hand location. We found that difference of the attentional peak between the two conditions is correlated with the shift of hand location by DHT. That is, visual attention is shifted to the location of illusory hand, suggesting that visual attention around peri-personal space is multimodal phenomenon.

## Keywords

flash-lag, visual attention, peripersonal space

## Acknowledgments

This study was supported by PRESTO, JST to K.M. and JSPS KAKENHI (JP16K12441) to S.S.

## Neural Correlates of Sound-Induced Visual Experience in Acquired Auditor-Visual Synesthesia

**Zixin Yong^1^, Po-Jang Hsieh^1^ and Dan Milea^2^**

^1^Duke-NUS Medical School, Singapore

^2^Singapore National Eye Center, Singapore Email: y.z@u.duke.nus.edu

## 

Auditory-visual synesthesia (AVS) could be acquired by a small number of patients suffered from visual impairments. The visual experiences evoked by auditory stimulations are often simple but pronounced (e.g., phosphenes). Despite some attempts to discern the neural correlates of the sound-induced visual experiences in acquired AVS, the exact brain regions that are involved remained elusive. In this study, we used fMRI to investigate this problem in an acquired AVS patient. During fMRI scan, pure tones of various pitches were presented to the patient, who was required to report the appearance of phosphenes by pressing one of two buttons for yes/no response. Besides response-related motor area activations, bilateral primary and secondary visual cortex activations were observed by contrasting phosphene trials with non-phosphene trials. In a control fMRI experiment, one blindfolded healthy participant was asked to signal the presence of light being flashed toward his face together with some tones. He was told that the light would be flashed in some trials, but in reality, no light was flashed. In this case, only motor area activation was observed by contrasting yes/no trials. Our results demonstrate that sound-induced visual experience in acquired AVS was correlated with bilateral primary and secondary visual cortex activation.

## Keywords

auditory visual synesthesia, acquired synesthesia, fMRI

## The Different Effects of Visual Perceptual Grouping on the Fission and Fusion Illusions

**Riku Asaoka^1^ and Yasuhiro Takeshima^2^**

^1^Tohoku University, Japan

^2^Doshisha University, Japan Email: R.Asaoka@dc.tohoku.ac.jp

## 

A single flash paired with two auditory beeps is often perceived as a double flash, while a double flash paired with one auditory beep is often perceived as a single flash. The former and latter phenomena are called the fission and fusion illusions, respectively. Previous studies showed that intramodal perceptual grouping interfered with intermodal perceptual grouping, resulting in diminished or reduced crossmodal effects. It remains unclear how visual perceptual grouping affects the fission and fusion illusions. The present study examined this issue using audiovisual inducer stimuli as incongruent cues. The participants were presented with the target flashes, visual inducers, and auditory inducers, and the number of these stimuli varied across trials. The task was to report their perceived number of the target flashes while ignoring the visual and auditory inducers. The results indicated that the fission illusion did not occur when a single target flash was accompanied by one visual and two auditory inducers. However, the fusion illusion occurred even when double target flashes were presented along with one auditory and two visual inducers. These results indicated that visual perceptual grouping reduced the occurrence rates of the fission illusion, but not the fusion illusion.

## Keywords

audio-visual integration, fission and fusion illusions, perceptual grouping

## Sensation Transference From Plateware to Food: The Sounds and Tastes of Plates

**Yi-Chuan Chen, Andy Woods and Charles Spence**

University of Oxford, UK Email: yi-chuan.chen@psy.ox.ac.uk

## 

Two experiments were designed to extend the well-known Bouba/Kiki effect to the case of an unusual set of commercially-produced plateware, and further to assess the influences of these plates on the expected taste of a dessert based on the theory of crossmodal correspondences. The results show that plates having a smoother circumference are more likely to be matched to “Bouba,” while those with a pointier circumference are more likely to be matched to “Kiki” instead, thus demonstrating the typical Bouba/Kiki effect. Both the shape and color of the plates modulated people’s ratings of the expected taste and liking of the dessert displayed on them. Specifically, the color of the plate induced a general effect on taste expectations that was consistent with the white-sweet and black-bitter associations. The shape of the plate modulated the rating of expected liking for the chocolate ice-cream, and the expected sweetness of the lemon sorbet, respectively. Finally, color and shape conjointly modulated the expected sourness of the lemon sorbet. The insights from such results are relevant to optimizing the visual appearance of specific dishes in restaurants and on product packaging.

## Keywords

Crossmodal correspondences, sound symbolism, Bouba-Kiki effect, color contrast, shape

## Acknowledgments

This study was supported by Arts and Humanities Research Council (AHRC), Rethinking the Senses grant (AH/L007053/1).

## Self-Motion Perception Induced by Visual Motion Without Luminance Modulation

**Shinji Nakamura**

Nihon Fukushi University, Japan Email: shinji@n-fukushi.ac.jp

## 

Uniform motion of large visual display which mostly occupies observer’s field of view can induce illusory self-motion perception toward the opposite direction (visually induced self-motion perception, also known as vection). Vection researches have indicated that effective vection induction might require luminance modulation in motion display, and visual motion without luminance modulation (so-called second order motion, such as contrast-defined or motion-defined motions) could not induce vection at all or could induce only a weak vection. The present study investigated possibilities of vection induction using non-luminance-based motion, using yet another type of motion perception, that is, orientation defined motion (fractal rotation; Benton et al., 2007). Psychophysical experiment in which 13 undergraduate observers were participated indicated that fractal rotation can induce illusory self-rotation (roll vection) with considerable strength, although it was a bit weaker than in a case of luminance-defined visual rotation. The results suggest that luminance modulation in visual motion is not essential for effective induction of self-motion perception.

## Keywords

self-motion perception, vection, fractal rotation

## Dissociating the Roles of Background Color and ipRGCs on Audiovisual Integration

**I-tan Weng^1^, Yi-Chuan Chen^2^, Li Chu^3^, Akiko Matsumoto^4^, Wakayo Yamashita^4^, Sei-ichi Tsujimura^4^ and Su-Ling Yeh^5,6,7^**

^1^Department of Psychology, National Taiwan University, Taipei, Taiwan

^2^Department of Experimental Psychology, University of Oxford, Oxford, UK

^3^Department of Psychology, The Chinese University of Hong Kong, Hong Kong, China

^4^Department of Information Science and Biomedical Engineering, Kagoshima University, Kagoshima, Japan

^5^Department of Psychology, National Taiwan University, Taipei, Taiwan

^6^Department of Psychology, National Taiwan University, Taipei, Taiwan

^7^Neurobiology and Cognitive Neuroscience Center, National Taiwan University, Taipei, Taiwan Email: suling@ntu.edu.tw

## 

Superior colliculus has been demonstrated as a site of early multisensory integration in animal studies; it also receives inputs from intrinsically photosensitive retinal ganglion cells (ipRGCs) that is most sensitive to blue-light, peaking at 480 nm. We examine whether signals from ipRGCs modulate human multisensory integration by comparing behavioral performance under different visual backgrounds while keeping the luminance and the color constant. In the simultaneity judgement task, a flash and a beep were presented at various SOAs and participants had to report whether the two stimuli were presented simultaneously. Participants were better in discriminating audiovisual simultaneity at 100 nm in the visual-leading condition when the background was blue (higher ipRGC stimulations) than orange (lower ipRGC stimulations) (Experiment 1). Nevertheless, when the levels of ipRGC stimulation were manipulated by either presenting the backgrounds through filter lens to reduce the ipRGC stimulation (Experiment 2) or a multi-primary projector system (Experiment 3) to increase the ipRGC stimulation while the background colors were controlled constant using metamers, there was no modulatory effect on audiovisual simultaneity judgments. The modulation of blue light on the precision of human audiovisual simultaneity perception is likely associated with higher levels of visual processing rather than the direct inputs from ipRGCs.

## Keywords

audiovisual integration, blue light, ipRGC, multisensory, metamers

## Acknowledgments

This study was supported by Taiwan’s Ministry of Science and Technology under grant no. 104-2420-H-002-003-MY3 to Dr. Su-Ling Yeh. We thank Prof. Homer Chen and Mr. Kuang-Tsu Shih for their assistance on the filter setup of Experiment 2.

## The Influence of Sound on Visual Global Motion Directional Discrimination: An Equivalent Noise Approach

**Ang-Ke Ku and Pi-Chun Huang**

National Cheng Kung University, Tainan, Taiwan Email: pichun_huang@mail.ncku.edu.tw

## 

Information from different sensory modalities are processed simultaneously and influenced by each other to help people interpret the environment. In this study, we focused on the audiovisual interactions in motion-integration processing. We used the equivalent noise paradigm to investigate how sound influences the global motion discrimination thresholds and detangled whether sound influences the precision of detecting the local motion direction (internal noise), the ability to pool these local motion signals across space (sampling efficiency) or both. The visual stimuli consisted of 100 dots, and we sampled the moving directions from a normal distribution with five levels of standard deviation (external noise). The observers discriminated the direction of the global motion under four conditions (absent, stationary, congruent, and incongruent sound). The psychometric function showed the directional sound bias in the observers’ responses by changing the guess rate. It showed that the thresholds increased with the levels of standard deviation whereas they were the same under the four sound conditions, which indicates that uninformative or informative sound did not influence the observers’ motion discrimination abilities. In conclusion, sound influenced neither the internal noise nor the sampling efficiency, but it influenced the directional-sound response bias on the decision level.

## Keywords

multisensory, audiovisual interactions, equivalent noise paradigm, global motion integration

## Acknowledgments

This study was supported by 104-2628-H-006-001-MY3 to PCH.

## Approaching Auditory Trees Make Wooden Sticks Feel Shorter

**Maiko Uesaki^1^, Hiroshi Ashida^2^, Akiyoshi Kitaoka^1^ and Achille Pasqualotto^3^**

^1^Ritsumeikan University, Japan

^2^Kyoto University, Japan

^3^Sabanci University, Turkey Email: uesaki@gst.ritsumei.ac.jp

## 

Increase in the retinal size of stationary objects in the environment is one of the cues to the observer’s forward motion. Here, a series of six images, each comprising a pair of dark pine-tree figures against a light background, were translated into the auditory modality using the vOICe software developed to assist the blind by converting visual scenes to sounds. Resulting auditory stimuli were either presented in a sequence (i.e., increasing in intensity and bandwidth conveying a pair of pine trees becoming larger in the visual field) or in a scrambled order. During the presentation of the auditory stimuli, blindfolded participants held one of the three wooden sticks of varying lengths at a time in their hands to estimate its length by free haptic exploration. Results showed that participants who listened to the auditory stimuli in a sequence, indicative of the listener’s motion towards the objects, underestimated the lengths of the sticks. The consistent underestimation observed in this study may be due to a mechanism similar to that underlying moving size-contrast illusions where an object surrounded by others increasing in size is perceived to be smaller than that surrounded by others of a constant size.

## Keywords

multisensory, sensory substitution, size contrast, illusion

## Acknowledgments

This study was supported by Japan Society for the Promotion of Science.

## The Validity of Facial and Vocal Cues: Testing the Backup Signal Hypothesis

**Zhi-Yun Liu and Wei-Lun Chou**

Department of Psychology, Fo Guang University, Taiwan Email: basilpudding@gmail.com

## 

Faces and voices may offer backup signals or multiple messages. We examined this debate by correlating perceived facial and vocal attractiveness in men and women. We also investigated whether facial and vocal cues are valid for raters to judge physical data of the models who provided photos as well as voices. We photographed and recorded 25 women and 25 men speaking five vowels. Standardized facial pictures and vocal samples were rated for attractiveness, height, body size, masculinity/femininity, and health by 64 participants. We found that the participants can accurately determine the height of the owner of a face and a voice. However, only the facial information but not the vocal information can be used to judge body size accurately. More importantly, the results showed that participants make similar judgments from photos and voices, with particularly strong correlations for height, body size, and masculinity/femininity. Moreover, visual and vocal attractiveness were found to positively correlate when men rated women. These results are interpreted as being consistent with the backup signal hypothesis.

## Keywords

backup signal hypothesis, face, voice, attractiveness

## Acknowledgments

This study was supported by MOST 104-2410-H-431-004.

## Approaching Sounds Dilate Perceived Time

**Achille Pasqualotto**

Faculty of Arts and Social Sciences, Sabanci University, Turkey Email: achille@sabanciuniv.edu

## 

Literature reports numerous examples of the effect of moving visual stimuli on time estimation. Here, we investigated the effect of auditory stimuli. Auditory stimuli were rendered by using the vOICe, a sensory substitution software converting visual images into the equivalent auditory ‘images’. We rendered: the sound of an approaching object, the sound of an object moving away from the listener and a ‘scrambled’ version of the previous two stimuli (baseline condition). These auditory stimuli were repeatedly played to blindfolded participants and represented the ‘background’ of the task; the ‘main task’ consisted of estimating the duration of target sounds. Target sounds were 300 Hz pure sounds, thus clearly distinguishable from the background. We found that, when participants were listening to the sound of approaching objects, they overestimated the duration of the target sounds. In other words, the sound of approaching objects dilated the perceived time. This bias can be interpreted as an evolutionary advantage, because overestimating time reduces the perceived distance between the listener and an approaching object, thus prompting faster behavioural responses.

## Keywords

time estimation, audition, looming, sensory substitution

## Transcranial Direct Current Stimulation Over the Medial Prefrontal Cortex Affects the Subjective Experience of Beauty

**Koyo Nakamura^1^ and Hideaki Kawabata^2^**

^1^Waseda University, Japan

^2^Keio University, Japan Email: kawabata@flet.keio.ac.jp

## 

Neuroaesthetics is concerned with the biological bases of the subjective experience of beauty. Neuroimaging studies have revealed that neural activity in the medial prefrontal cortex (mPFC) correlates with the subjective experience of visual beauty. However, correlational studies are poorly suited for demonstrating the causal relationship between subjective beauty and its neural underpinnings. To investigate the causal role of the mPFC on aesthetic appreciation, we applied transcranial direct current stimulation (tDCS) and examined whether non-invasive brain stimulation modulates aesthetic appreciation of abstract artworks. In the experiment, participants rated the subjective beauty and ugliness of abstract paintings on a nine-point rating scale before and after the application of tDCS on the mPFC. The application of cathodal tDCS over the mPFC, which induced temporal inhibition of neural excitability in the region, led to a decrease in beauty ratings but not ugliness ratings, while the application of sham stimulation over the mPFC did not impact beauty and ugliness ratings. The results of our experiment indicate that the mPFC has a causal role in generating the subjective experience of beauty.

## Keywords

neuroaesthetics, visual beauty, transcranial direct current stimulation, medial prefrontal cortex

## Acknowledgments

This study was supported by JSPS KAKENHI (26119525) to Hideaki Kawabata and Grant-in-Aid for JSPS Fellows (15J08281) to Koyo Nakamura.

## Neuro-Behavioral Assessment of Visual Performance and Discomfort in High Luminance Displays

**Shun-nan Yang^1^, Ju Liu^1^ and Manho Jang^2^**

^1^Vision Performance Institute, Pacific University College of Optometry, Forest Grove, OR, USA

^2^DON Silicon Valley R/D Center, Santa Clara, CA, USA Email: shunnan.yang@pacificu.edu

## 

Excessive luminance bleaches photoreceptors and overstimulates the primary visual cortex, which can lead to reduced visual sensitivity and increased discomfort. Images rendered with high dynamic range (HDR) methods can expand the luminance distribution shown on digital displays. Temporally, the dynamic luminance change in images can be exacerbated by image flickering. The present study investigated how temporally modulated luminance change impedes visual processing and affects viewing comfort. Participants with normal vision viewed blocks of trials alternating between a homogenous luminance circle and a suprathreshold grating pattern. They were asked to identify the direction of grating as soon as possible, while their EEG signals were recorded. The circle luminance was identical within each block of trials and randomized across blocks. Results show a positive correlation between luminance level and viewing symptoms, and a negative one between luminance and event-related VEP amplitude and discrimination accuracy. Chromatic luminance stimuli revealed that such sensitivity was specific to particular color opponency pathways and varied individually. These findings suggest a luminance threshold at around 650 nits in displaying dynamic image with which the visual processing is not significant impeded where the visual abilities are preserved. Such a threshold varies dependently on individual differences in luminance and chromatic sensitivity.

## Keywords

VEP, visual perception, luminance, visual flicker, visual discomfort

## Acknowledgments

This study was supported by LG Display.

## Landscape Preference In Taiwanese School-Aged Children

**Chien Kai Chang^1^, Shu-Fei Yang^2^, Li-Chih Ho^3^ and Hui-Lin Chien^4,5^**

^1^Graduate Institute of Biomedical Sciences, China Medical University, Taichung, Taiwan

^2^Graduate Institute of Neural and Cognitive Sciences, China Medical University, Taichung, Taiwan

^3^Department of Environmental and Hazards-Resistant Design Huafan University, New Taipei City, Taiwan

^4^Graduate Institute of Biomedical Sciences, China Medical University, Taichung, Taiwan

^5^Graduate Institute of Neural and Cognitive Sciences, China Medical University, Taichung, Taiwan Email: sarinachien@mail.cmu.edu.tw

## 

We are fond of beautiful scenery, but not all types of scenery are equally fascinating. A recent study using visual signal computational model to predict landscape preference discovered that Taiwanese young adults showed a higher preference for natural scenes than urban scenes. The present study aimed to explore the landscape preference in Taiwanese school-aged children (5- to 12 year old) using the same image database. In this study, each participant received 80 pictures containing four natural scenes (coasts, forests, countrysides, mountains views) and four urban scene (highways, tall buildings, streets, inner cities) types, 10 for each type. There were six different sets of 80 pictures from the 480-picture image database (Ho et al., 2015). The child participants were asked to rate their preference for each picture from one (strongly disliked) to five (strongly liked). We found that Taiwanese children showed a significantly higher preference for natural scenes than urban scenes, and their preference of the coast scenes was the highest among all types. The present study revealed that, like adults, Taiwanese children exhibited a stronger preference for natural scenes than urban scenes, which supports the prospect-refuge theory that natural scenes simultaneously provide abundance and a sense of security to meet human needs.

## Keywords

environmental cognition, aesthetic preference, aesthetic perception, landscape perception, cognitive development

## Acknowledgments

This study was supported by MOST 105-2420-H-039-001-MY3, MOST 105-2632-B-039-003- to Dr. Chien.

## The Salient Partner: Identity-Referential Saliency Evoked by Physical Presence

**Miao Cheng^1^ and Chia-huei Tseng^2^**

^1^University of Hong Kong, Hong Kong

^2^Research Institute of Electrical Communication, Tohoku University, Japan Email: chengmiao@hku.hk

## 

Neutral information enjoys prioritized processing when associated with self or significant others. However, it remains unclear what contributes to identity referential saliency. We examined whether familiarity was necessary to create identify-related advantage by introducing a stranger as partner. Participants associated three geometric shapes with own, partner’s and stranger’s names, and reported whether name–shape parings correctly matched. We misguided participants to believe that after individual condition, they would perform the task together with their partner, while in reality all participants only performed the task individually. In Experiment 1, each participant met his/her assigned partner briefly without further communication; while in Experiment 2, the partner never appeared physically. Consistent with previous studies, self-related trials received processing advantage (higher accuracy, shorter response time) than partner- and stranger-related trials in both experiments. More importantly, trials related to a partner’s name also receive similar advantage than those related to a stranger’s name in Experiment 1, but this partner advantage disappeared in Experiment 2. This novel discovery suggested that identity referential saliency can be quickly built up towards a stranger without prior familiarity, and physical presence is a substantial contributor. Our study has theoretical implication for understanding the nature of identity referential saliency and disassociating self- and other advantages.

## Keywords

identity referential saliency, partner advantage, self-advantage, perceptual association
